# A potent pan-sarbecovirus neutralizing antibody resilient to epitope diversification

**DOI:** 10.1016/j.cell.2024.09.026

**Published:** 2024-10-08

**Authors:** Laura E. Rosen, M. Alejandra Tortorici, Anna De Marco, Dora Pinto, William B. Foreman, Ashley L. Taylor, Young-Jun Park, Dana Bohan, Tyson Rietz, John M. Errico, Kevin Hauser, Ha V. Dang, Justin W. Chartron, Martina Giurdanella, Giuseppe Cusumano, Christian Saliba, Fabrizia Zatta, Kaitlin R. Sprouse, Amin Addetia, Samantha K. Zepeda, Jack Brown, Jimin Lee, Exequiel Dellota, Anushka Rajesh, Julia Noack, Qiqing Tao, Yvonne DaCosta, Brian Tsu, Rima Acosta, Sambhavi Subramanian, Guilherme Dias de Melo, Lauriane Kergoat, Ivy Zhang, Zhuoming Liu, Barbara Guarino, Michael A. Schmid, Gretja Schnell, Jessica L. Miller, Florian A. Lempp, Nadine Czudnochowski, Elisabetta Cameroni, Sean P. J. Whelan, Hervé Bourhy, Lisa A. Purcell, Fabio Benigni, Julia di Iulio, Matteo Samuele Pizzuto, Antonio Lanzavecchia, Amalio Telenti, Gyorgy Snell, Davide Corti, David Veesler, Tyler N. Starr

**Affiliations:** 1 Vir Biotechnology, San Francisco, CA 94158, USA.; 2 Department of Biochemistry, University of Washington, Seattle, WA 98195, USA.; 3 Humabs BioMed SA, a subsidiary of Vir Biotechnology, 6500 Bellinzona, Switzerland.; 4 Department of Biochemistry, University of Utah School of Medicine, Salt Lake City, UT 84112, USA; 5 Howard Hughes Medical Institute, University of Washington, Seattle, WA 98195, USA.; 6 ProtaBody, Pasadena, CA 91105, USA.; 7 Institut Pasteur, Université Paris Cité, Lyssavirus Epidemiology and Neuropathology Unit, Paris, France.; 8 Computational and Systems Biology Program, Sloan Kettering Institute, Memorial Sloan Kettering Cancer Center, New York, NY 10065, USA.; 9 Tri-Institutional PhD Program in Computational Biology and Medicine, Weill Cornell Graduate School of Medical Sciences, New York, NY 10065, USA.; 10 Department of Molecular Microbiology, Washington University School of Medicine, St. Louis, MO 63110, USA.

## Abstract

SARS-CoV-2 evolution has resulted in viral escape from clinically authorized monoclonal antibodies (mAbs), creating a need for mAbs that are resilient to epitope diversification. Broadly neutralizing coronavirus mAbs that are sufficiently potent for clinical development and retain activity despite viral evolution remain elusive. We identified a human mAb, designated VIR-7229, which targets the viral receptor-binding motif (RBM) with unprecedented cross-reactivity to all sarbecovirus clades, including non-ACE2-utilizing bat sarbecoviruses, while potently neutralizing SARS-CoV-2 variants since 2019, including the recent EG.5, BA.2.86, and JN.1. VIR-7229 tolerates extraordinary epitope variability, partly attributed to its high binding affinity, receptor molecular mimicry, and interactions with RBM backbone atoms. Consequently, VIR-7229 features a high barrier for selection of escape mutants, which are rare and associated with reduced viral fitness, underscoring its potential to be resilient to future viral evolution. VIR-7229 is a strong candidate to become a next-generation COVID-19 medicine.

## eToC blurb

A monoclonal antibody VIR-7229 uniquely combines potent SARS-CoV-2 inhibition via ACE2 competition, with pan-sarbecovirus and pan-variant cross-reactivity and a high barrier to viral escape. This antibody may be resilient to SARS-CoV-2 evolution, with potential to be a next-generation COVID-19 medicine and a key component of pandemic preparedness in the event of a novel emerging sarbecovirus.

## Graphical Abstract

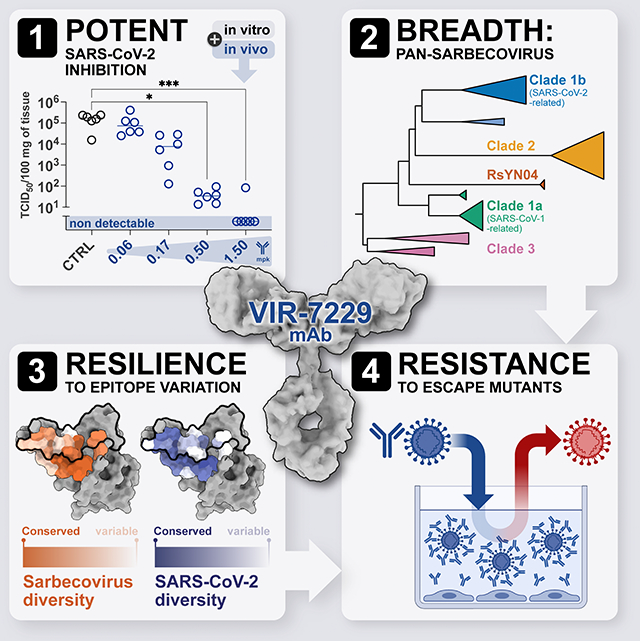

## Introduction

Four and a half years after the emergence of SARS-CoV-2, the disease burden from COVID-19 remains high particularly for immunocompromised individuals and those at risk of severe disease.^1,2^ Monoclonal antibodies (mAbs) are an important tool in the prevention and treatment of COVID-19 for these patient populations,^3–7^ with millions of doses administered during the pandemic.^8^ However, mutations arising from continued SARS-CoV-2 evolution have abolished the activity of most approved therapeutic mAbs.^9–12^ Therefore, there remains a need for potent COVID-19 mAbs with durable activity in the face of continuous antigenic change.

One strategy for identifying mAbs resilient to viral evolution is to evaluate epitope conservation in distantly related viruses. The only therapeutic or prophylactic mAbs with activity against the majority of SARS-CoV-2 variants to-date are sotrovimab,^13,14^ derived from the S309 mAb,^15^ and pemivibart (VYD222),^16,17^ which was affinity-matured from the ADI-55688 mAb.^4,18,19^ S309 and ADI-55688 were selected from the memory B cells of 2003 SARS-CoV-1 survivors based on their ability to recognize epitopes conserved between SARS-CoV-2 and SARS-CoV-1.^15,18^ Following the identification of S309 and ADI-55688, additional conserved SARS-CoV-2 spike (S) epitopes have been identified as targets of mAbs that are broadly reactive with all sarbecoviruses,^20,21^ beta-coronaviruses,^22,23^ or even with multiple coronavirus genera.^24,25^ However, these highly-conserved epitopes are distinct from those targeted by the most potent mAbs, which usually compete directly with the ACE2 host receptor for binding to the SARS-CoV-2 S receptor-binding motif (RBM).^26,27^ The RBM is one of the most variable S regions (Figure S1A) due to the strong immune pressure at this site^28,29^ and the plasticity of the binding interface between the receptor-binding domain (RBD) and ACE2.^9,30–32^ As a result, RBM-targeting mAbs are generally SARS-CoV-2-specific^33^ and their neutralizing activity is frequently abolished due to viral evolution.^34^ This apparent trade-off between neutralization breadth and potency has posed a major challenge for the clinical development of mAbs with broad neutralizing activity as their low neutralization potency would require using high doses, leading to increased manufacturing costs, more frequent administration, and/or lengthy intravenous infusions.

Here, we describe a highly potent SARS-CoV-2 RBM-targeting mAb, designated VIR-7229, which has unprecedented activity across the entire family of sarbecoviruses. VIR-7229 tolerates a remarkable sequence diversity in its epitope, and the rare escape mutations discovered in vitro are associated with reduced viral fitness, resulting in a high barrier for the emergence of resistance. Our findings indicate VIR-7229 has a high probability to be resilient to future SARS-CoV-2 evolution, positioning it as a promising investigational COVID-19 medicine.

## Isolation of a potent pan-sarbecovirus neutralizing mAb

To identify broadly reactive mAbs, we interrogated with a high-throughput method the memory B cells of individuals who had received two to three doses of a Wuhan-Hu-1 S SARS-CoV-2 vaccine and who were subsequently infected by Omicron variants in 2022. A candidate mAb (S2V29) was selected based on its potent neutralization of pre- and post-Omicron SARS-CoV-2 variants, as well as for its cross-reactivity with a panel of sarbecovirus RBDs, including SARS-CoV-1. To further improve its cross-reactivity and neutralization potency, S2V29 was affinity-matured using both SARS-CoV-1 and SARS-CoV-2 BQ.1.1 RBDs as target antigens. We utilized a yeast display system combined with a machine learning (ML)-guided approach for library design and analysis.^35–37^ This approach relies on sequencing every mAb variant in a training library after sorting based on binding affinity to the target antigens. These data enable ML-based predictions of the properties of mAb variants not present in the library, allowing investigation of a larger number of antibody variants than traditional affinity maturation.^38^ After two rounds of library screening and ML model training, a set of 56 mAb sequences were selected for recombinant production. These mAb variants were screened for binding and neutralization of a wide panel of SARS-CoV-2 variants and other sarbecoviruses to select the mAbs with the highest breadth and potency.

**Figure 1.**
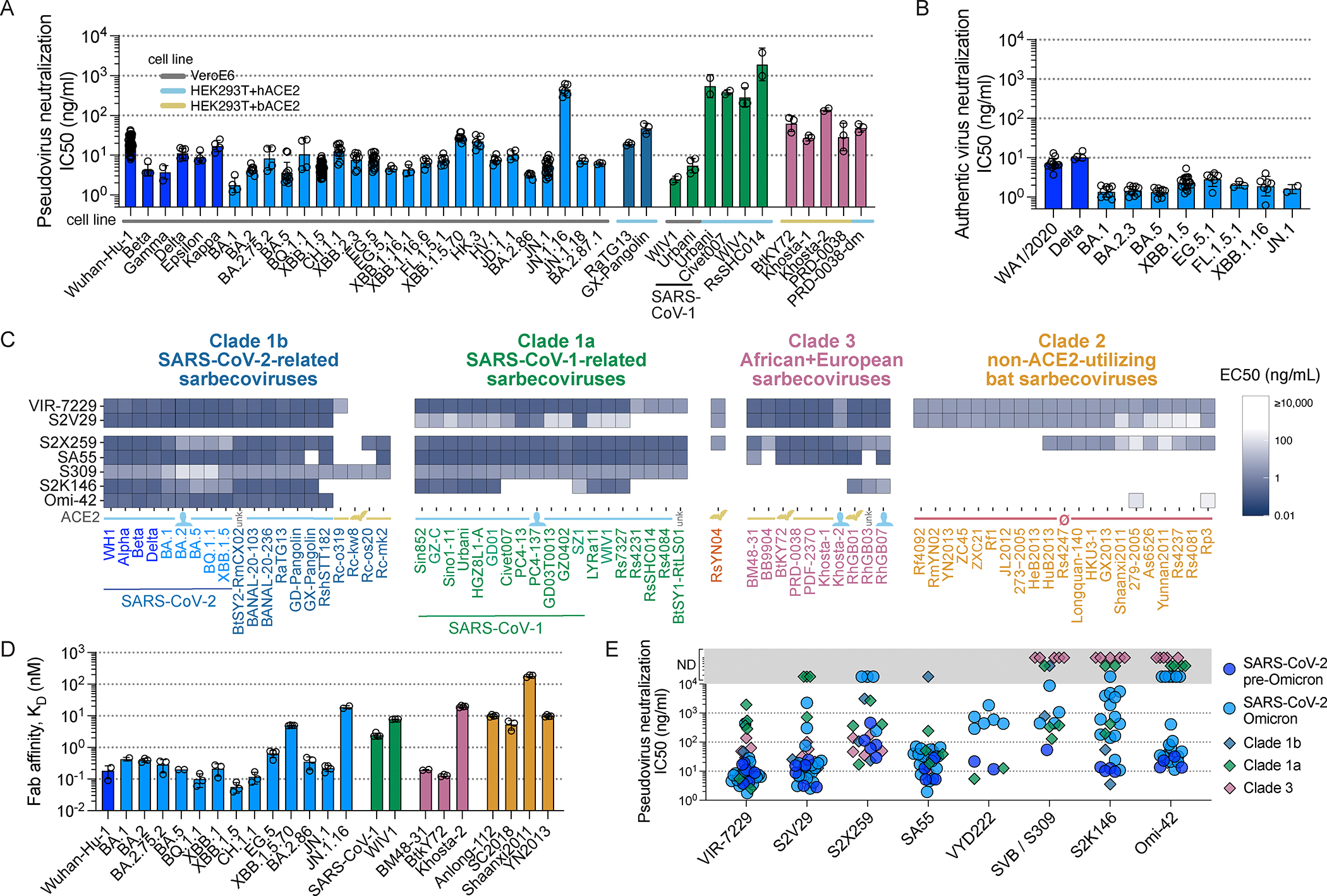
VIR-7229 is a potent, pan-sarbecovirus neutralizing mAb. (A) VIR-7229-mediated neutralization of pseudoviruses. Bar color denotes sarbecovirus clade, as in panel C. Horizontal lines denote cell line employed in neutralization assay: VeroE6 (gray), HEK-293T+human ACE2 (cyan), HEK-293T+bat (*R. alcyone*) ACE2 (yellow). PRD0038-dm refers to PRD0038 harboring the K482Y/T487W RBD mutations (SARS-CoV-2 numbering 493Y/498W), which allow for entry using human ACE2.^39,40^ SARS-CoV-1 Urbani and WIV1 experiments were run in two assay conditions. See also Data S1. See Figure S2 for neutralization mechanisms of action. (B) VIR-7229-mediated neutralization of authentic SARS-CoV-2 virus, performed with VeroE6 cells. The WA1/2020 isolate has the same S haplotype as Wuhan-Hu-1. See also Data S1. (C) Breadth of VIR-7229 and comparator mAbs binding to a yeast-displayed panel of sarbecovirus RBDs spanning the known phylogenetic diversity. Line below the graph, denoted by “ACE2,” indicates whether a sarbecovirus binds or enters cells via human ACE2 (blue), bat but not human ACE2 (yellow), no ACE2 (pink), or unknown (unk.). See Figure 4A for phylogenetic relationships and clade definitions. See Data S1 for full sequences, phylogeny, and alignment. (D) VIR-7229 Fab fragment binding affinity measured by SPR. Bar color denotes sarbecovirus clade. SARS-CoV-1 is Urbani. See also Data S1. (E) Overview of pseudovirus neutralization by comparator mAbs, colored by sarbecovirus clade. Data points within the gray bar represent neutralization not detected (ND), i.e. IC50 >10,000 ng/ml. See Figure S1 and Data S1 for data separated by strain.

Although the affinity maturation approach yielded candidate mAb sequences containing up to 11 mutations relative to the S2V29 mAb, one of the top neutralizers contained only two mutations (heavy chain V50Y and N57D) and was selected as the lead candidate (designated VIR-7229). Remarkably, VIR-7229 neutralized SARS-CoV-1 two orders of magnitude more potently than S2V29 (Figures 1A, S1B–C). VIR-7229 competed with ACE2 for binding to the SARS-CoV-2 RBD (Figure S2A) and neutralized a large panel of pre- and post-Omicron vesicular stomatitis virus (VSV)-based SARS-CoV-2 S pseudotyped viruses (pseudoviruses) with high potency (28-strain panel: IC_50_ 1.8–435 ng/ml, median 7.3 ng/ml; Figure 1A; Data S1), as well as representative VSV-based pseudoviruses selected from all ACE2-utilizing sarbecovirus clades (Figure 1A; Data S1). Neutralization of authentic SARS-CoV-2 viral isolates was consistent with the high potency observed with pseudoviruses (10-strain panel: IC_50_ 1.3–10.3 ng/ml, median 2.0 ng/ml; Figure 1B).

To further investigate the breadth of this unique RBM-specific mAb, we evaluated its binding to a yeast-displayed library of RBDs encompassing 71 strains spanning the known sarbecovirus phylogenetic diversity (Figure 1C; Data S1). Strikingly, VIR-7229 recognized every sarbecovirus RBD tested known to bind or enter cells using the human ACE2 receptor, as well as divergent bat-ACE2-utilizing sarbecoviruses (e.g. RsYN04, PRD-0038, and BtKY72), some of which can evolve to bind human ACE2 via single amino acid changes.^39,40^ Furthermore, VIR-7229 bound to all the divergent, non-ACE2-utilizing clade 2 sarbecovirus RBDs tested, which is an unprecedented result due to the presence of two large deletions in clade 2 RBMs which typically disrupt recognition by RBM-targeting mAbs^20^ (Data S1). The only three RBDs in our panel not recognized by VIR-7229 are viruses from Japan related to Rc-o319, which have a narrow host specificity for the geographically-isolated *Rhinolophus cornutus* bat species.^41^ Concurring with the yeast-display data, surface plasmon resonance (SPR) experiments showed that the VIR-7229 Fab fragment binds with sub-nanomolar affinity to most clade 1b (14-strain SARS-CoV-2-variant panel: K_D_ 0.05–19 nM, median 0.29 nM) and clade 3 RBDs and with nanomolar affinity to the other sarbecovirus RBDs (Figure 1D; Data S1). To ensure that the extensive cross-reactivity of VIR-7229 was not due to binding promiscuity, we performed assessments of polyreactivity (Hep2 cells) and tissue cross-reactivity (immunohistochemical screening of 39 human tissues) with both S2V29 and VIR-7229 and observed no off-target binding for either mAb (Data S2).

To benchmark the breadth and potency of VIR-7229, we compared pseudovirus neutralization and RBD binding side-by-side with a panel of previously-described broadly reactive mAbs^15,21,27,33,42^ (Figures 1C, 1E, S1D–J). Similar to VIR-7229, S2X259 (antigenic site IIa) cross-reacted with members of all four sarbecovirus RBD clades although it recognized fewer clade 2 RBDs. Moreover, S2X259 had lower neutralization potency against SARS-CoV-2 Wuhan-Hu-1, relative to VIR-7229, and lost activity against recent Omicron variants. S309 (sotrovimab parent, antigenic site IV) cross-reacted with clade 1b and 1a RBDs, including the divergent bat ACE2-utilizing Rc-o319-related viruses, but not with clade 2 and 3 RBDs. The moderately-potent VYD222 mAb^16,17^ (antigenic site Ia; renamed pemivibart and which received an emergency use authorization in the United States during the revision of this manuscript) cross-reacted with clade 1a RBDs, as well as two clade 3 RBDs tested, but did not cross-react with all clade 3 or with any clade 2 RBDs tested (Figures S1I–J). Potent RBM-targeting (antigenic site Ia) mAbs S2K146, Omi-42, and SA55 revealed distinct patterns of cross-reactivity. S2K146 is a unique example of an RBM-specific mAb isolated in the pre-Omicron era that retains activity, albeit reduced, against the majority of Omicron variants to date,^43^ possibly due to its receptor molecular mimicry.^33^ S2K146 cross-reacted with SARS-CoV-1 and some related clade 1a RBDs, as well as select clade 3 RBDs. Omi-42, which is in clinical development in the United States,^44^ bound to human ACE2-utilizing clade 1b RBDs, neutralized the recently circulating JN.1 variant with moderate potency, and did not neutralize F456L-harboring SARS-CoV-2 XBB-lineage^45^ or JN.1-lineage variants. Finally, SA55, in clinical development in China,^46^ broadly reacted with most ACE2-utilizing RBDs but did not cross-react with any of the clade 2 RBDs in our panel. Overall, the broad sarbecovirus reactivity of VIR-7229 is unique among potently neutralizing RBM-directed antibodies, establishing it as a best-in-class neutralizing mAb.

We next characterized the ability of VIR-7229 to promote S_1_ shedding and Fc-mediated effector functions. As observed for other RBM-targeting mAbs,^28,47^ VIR-7229 efficiently triggered S_1_ shedding (Figure S2B), a mechanism that can contribute to viral neutralization.^47^ Possibly due to S_1_ shedding kinetics, VIR-7229 weakly activated FcγRIIa and FcγRIIIa in vitro using a Jurkat cell line-based reporter assay (Figures S2C, S2D). Using primary human effector cells and highly sensitive HiBiT target cells, we observed only moderate antibody-dependent cell cytotoxicity (ADCC) (Figure S2E). These findings suggest that the main mechanisms of VIR-7229 antiviral activity are ACE2 competition and possibly S_1_ shedding, with only a minor potential contribution of effector function.

**Figure 2.**
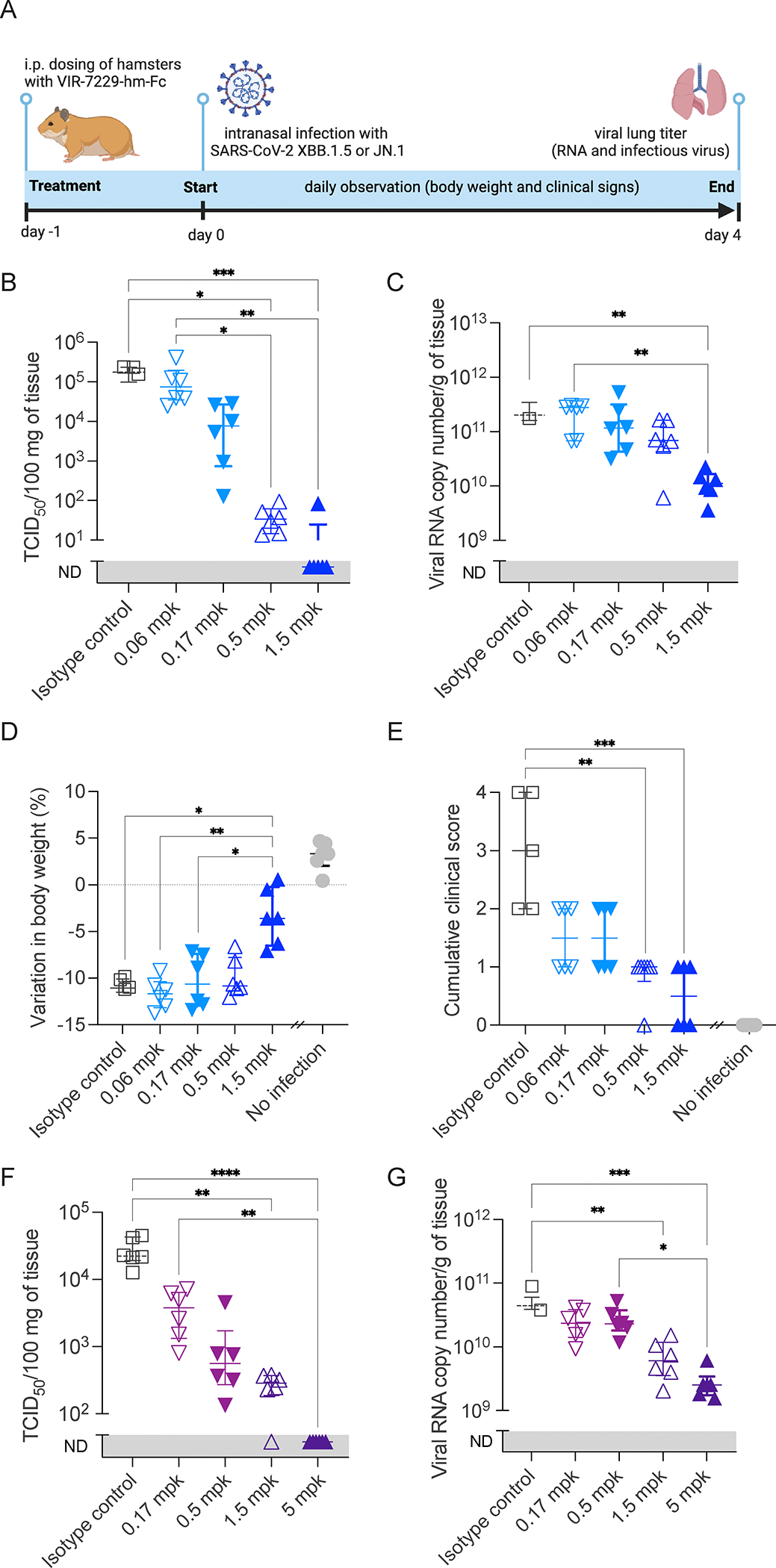
In vivo efficacy of VIR-7229. Virology and clinical endpoints on day 4 after SARS-CoV-2 XBB.1.5 or JN.1 infection of Syrian hamsters prophylactically administered with VIR-7229 (hamster Fc) or 1.5 mg/kg (mpk) isotype-matched control antibody. See also Data S3. (A) Experiment outline. (B) Infectious viral lung titers for XBB.1.5 infection. ND: not detected. (C) Lung viral RNA load for XBB.1.5 infection. ND: not detected. (D) Variation in body weight relative to day 0 for XBB.1.5 infection. No-infection control from the JN.1 experiment is provided for qualitative comparison. (E) Cumulative clinical score for XBB.1.5 infection (0–4): ruffled fur, slow movements, apathy, absence of exploratory activity. No-infection control from the JN.1 experiment is provided for qualitative comparison. (F) Infectious viral lung titers for JN.1 infection. (G) Lung viral RNA load for JN.1 infection. ND: not detected. (B-G) X-axis indicates dose of VIR-7229-hmFc or 1.5 mpk isotype control. Median ± interquartile range is shown; significance is based on ANOVA non-parametric Kruskal-Wallis test followed by Dunn’s multiple comparison test, *p<0.05, **p< 0.01, *** p < 0.001.

To investigate whether the potent VIR-7229-mediated in vitro neutralization translates into effective in vivo protection, we evaluated mAb prophylactic activity using a Syrian hamster model of infection. Animals were administered intraperitoneally with VIR-7229-hm-Fc (VIR-7229 with a species-matched Fc) at various doses one day before challenge with SARS-CoV-2 XBB.1.5 or JN.1. VIR-7229 reduced viral RNA and infectious viral titers in the lungs of challenged animals in a dose-dependent manner, with the highest dose (1.5 mg/kg and 5 mg/kg, for XBB.1.5 and JN.1 challenge, respectively) resulting in infectious virus titers below the limit of detection (Figure 2; Data S3). Furthermore, VIR-7229 administration protected the XBB.1.5-challenged animals from weight loss (Figure 2; Data S3; JN.1 infection induced mild clinical symptoms in hamsters and none of the JN.1-challenged animals experienced weight loss at day 4). Overall, VIR-7229 is endowed with broad sarbecovirus cross-reactivity, potent neutralizing activity and protective prophylactic efficacy that are collectively unparalleled among previously characterized COVID-19 mAbs.

## Structural basis of VIR-7229 breadth and potency

**Figure 3.**
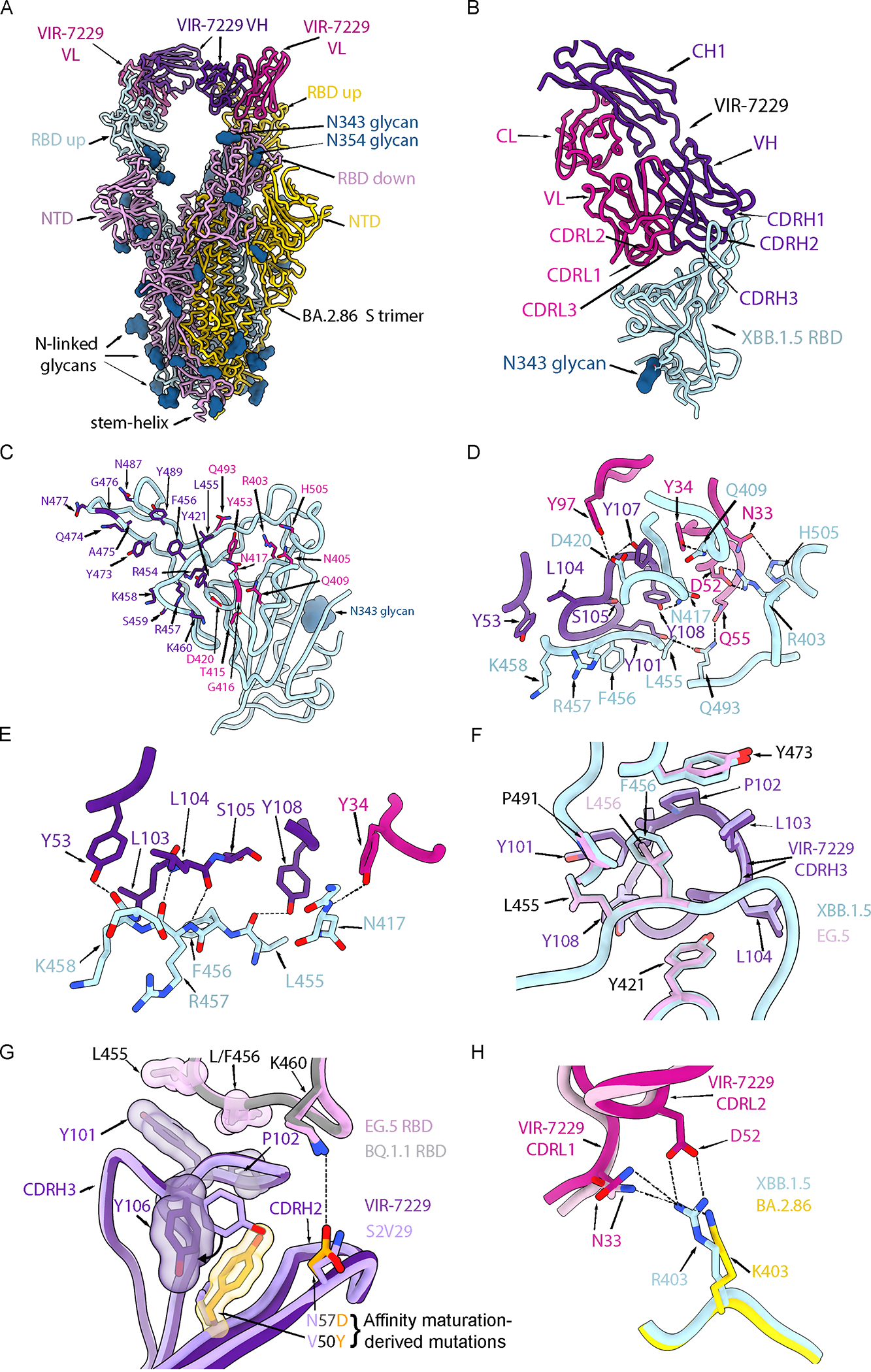
Structural basis for VIR-7229 pan-sarbecovirus neutralization. (A) Ribbon diagram of the cryoEM structure of the BA.2.86 S ectodomain trimer (cyan, pink and gold) in complex with two VIR-7229 Fabs (purple and magenta) with N-linked glycans rendered as blue surfaces. See also Figure S3. (B) Ribbon diagram of the VIR-7229-bound XBB.1.5 RBD crystal structure. The bound S309 Fab is omitted for clarity. The N343 glycan is rendered as a blue surface. See also Figure S4. (C) XBB.1.5 RBD (cyan) with VIR-7229 epitope residues shown as sticks and colored according to the (dominant) Fab interacting chain. RBD residues 420, 453, 455, 460 and 493 interact with the VIR-7229 heavy and light chains and were colored based on the chain with which they bury the greatest surface area. (D) Zoomed-in view of select interactions formed between VIR-7229 and the XBB.1.5 RBD. Hydrogen bonds and salt bridges are indicated with black dash lines. (E) Zoomed-in view of hydrogen-bonds (black dash lines) formed between VIR-7229 and the RBD backbone. (F) Superposition of the VIR-7229-bound XBB.1.5 RBD (cyan RBD, dark purple mAb) and VIR-7229-bound EG.5 RBD (pink RBD, light purple mAb) showing accommodation of the F456L residue mutation. (G) Superposition of the VIR-7229-bound EG.5 RBD (pink RBD, dark purple mAb) and S2V29-bound BQ.1.1 RBD (gray RBD, light purple mAb). The two CDRH3 residues differing between S2V29 and VIR-7229 (V50Y and N57D) are highlighted in orange. Select residues from the VIR-7229:EG.5 RBD structure are also shown as semi-transparent surfaces colored according to the sticks. (H) Superposition of the VIR-7229-bound XBB.1.5 RBD (cyan RBD, dark pink mAb) and VIR-7229-bound BA.2.86 S (gold RBD, bright pink mAb) structures highlighting the conservation of electrostatic interactions (dashed lines) at the epitope/paratope interface due to the BA.2.86 R403K mutation. The D52 side chain is weakly resolved in the BA.2.86 S cryoEM density and was therefore not modeled.

To understand the molecular basis of the remarkable VIR-7229 breadth, we determined a cryoEM structure of the VIR-7229 Fab fragment bound to the BA.2.86 S ectodomain trimer at an overall resolution of 3.1 Å. Local refinement of the VIR-7229 Fab variable domains and the BA.2.86 RBD yielded a reconstruction at 3.3 Å resolution (Figures 3A, S3; Table S1). Moreover, we determined crystal structures of the VIR-7229 Fab fragment bound to XBB.1.5 and to EG.5 (XBB.1.5 + F456L) RBDs at 2.4 and 1.95 Å resolution, respectively, as well as of the parent S2V29 Fab bound to BQ.1.1 RBD at 1.67 Å resolution (Figure 3B–G; Table S2). VIR-7229 recognizes an epitope in the RBD antigenic site Ia,^28^ which overlaps with the ACE2-binding site (i.e. the RBM), burying an average of 950 Å^2^ at the interface between the epitope and the paratope (Figure 3A–B). VIR-7229 interacts with the RBD via polar interactions and shape complementarity mediated by all six CDR loops, with the heavy chain CDR3 (H3) dominating the paratope. The epitope comprises amino acid residues 403, 405, 409, 415–417, 420–421, 453–460, 473–477, 487, 489, 493 and 505 (Figure 3C). Thirteen out of these 25 residues participate in binding to human ACE2, explaining the competition observed for receptor engagement (Figures S2A, S4A). Strikingly, VIR-7229 binding induces a rearrangement of RBD residues 473–489 (Figure S4B), which are shifted approximately 5.5 Å relative to structures of apo S or of the RBD in complex with ACE2.^13,27,48,49^

**Figure 4.**
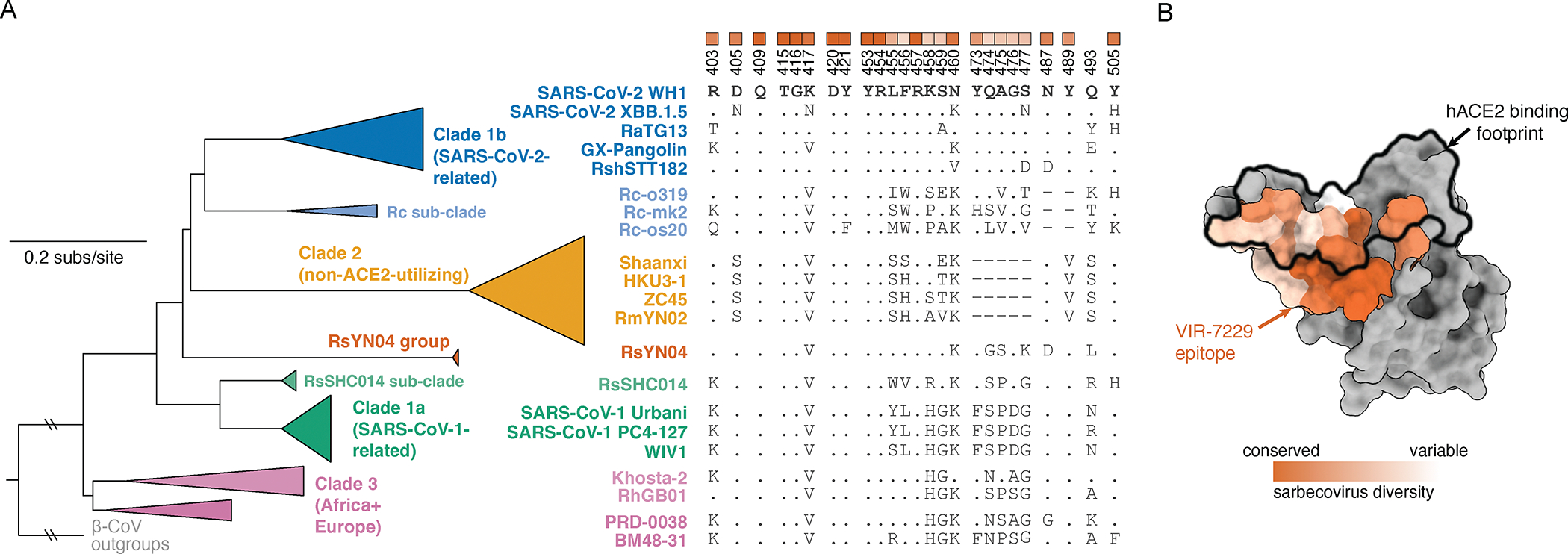
VIR-7229 epitope encompasses sarbecovirus diversity. (A) Collapsed sarbecovirus phylogeny (left) with multiple sequence alignment of select sarbecoviruses (right) illustrating variation at VIR-7229 epitope positions. RBD numbering is relative to SARS-CoV-2. Dots indicate the SARS-CoV-2 Wuhan-Hu-1 identity. Heatmap at top of alignment illustrates extent of variation (white) or conservation (orange) across the entire sarbecovirus alignment, matched to the structural mapping in panel B. See Data S1 for full phylogeny and alignment. (B) Sarbecovirus conservation of the VIR-7229 epitope mapped to SARS-CoV-2 Wuhan-Hu-1 RBD structure (PDB 6M0J). ACE2 binding footprint is illustrated as a black outline.

VIR-7229 CDR H3 forms extensive contacts with the RBD, burying ~470 Å^2^ of its surface at the interface with SARS-CoV-2 RBD residues 415–417, 420–421, 454–460, 473, 489 and 493 (Figure 3D–E). Five out of 16 hydrogen bonds between VIR-7229 and the RBD involve RBD backbone rather than amino acid side chains (RBD residues N417, L455, R457, K458), which may contribute to the resilience of VIR-7229 to epitope diversification (Figure 3E). Examples include hydrogen bonds formed by the RBD-L455 backbone carbonyl oxygen with H3-Y108 side chain hydroxyl and by the RBD-R457 backbone amide and carbonyl oxygen with H3-L104 backbone carbonyl oxygen and amide. Furthermore, comparison of the VIR-7229-bound XBB.1.5 and EG.5 structures shows that the VIR-7229 binding mode enables H3 to accommodate equally effectively F456 or L456 in these variant backgrounds (Figure 3F), the latter mutation mediating immune evasion of many mAbs targeting antigenic site Ia.^50^ Molecular dynamics (MD) simulations performed on both VIR-7229:XBB.1.5 RBD and VIR-7229:EG.5 RBD structures (total simulation time 4.0 μs for each) indicated that residue 456 is one of the RBD positions (along with 415) with which VIR-7229 makes the largest number of persistent contacts for both F456 (XBB.1.5) and L456 (EG.5) (Figure S4C; Data S4). The affinity maturation of S2V29 to VIR-7229 selected for two amino acid changes in CDR H2 (V50Y and N57D) and resulted in marked improvement of neutralization potency against SARS-CoV-1 (Figure S1B), which harbors a leucine at the position equivalent to SARS-CoV-2 residue 456 (Figure 4A). Comparison of the S2V29-bound and VIR-7229-bound RBD structures suggests that the N57D substitution allows formation of a salt bridge with RBD K460 (clearly resolved in the EG.5 RBD structure), most likely strengthening binding (Figure 3G). Although CDR H2 residue 50 does not directly contact the RBD, the V50Y substitution leads to reorientation of the neighboring H3-Y106 side chain and formation of T-shaped pi stacking interactions between the two aromatic rings, possibly preconfiguring CDR H3 for binding (Figure 3G).

The above structural findings likely explain the overall resilience of VIR-7229 to mutations observed in circulating strains at RBD residues 455 and 456 (Figures 1A, 1B, 1D), which individually or jointly appeared in descendant lineages of XBB.1.5 (e.g. HK.3) and BA.2.86 (e.g. JN.1) and have dampened the neutralizing activity of polyclonal plasma antibodies in individuals exposed to XBB.1.5.^50–52^ Furthermore, our structural data explain the potent VIR-7229-mediated neutralization of BA.2.86 and JN.1; the latter variant is the parental lineage for the dominant currently-circulating strains. Out of the 11 mutated residues in the BA.2.86 RBD relative to XBB.1.5, only R403K is found in the VIR-7229 epitope, a substitution which would preserve electrostatic interactions with the VIR-7229 light chain N33 and D52 amide and carboxylate side chains, respectively, as observed in our cryoEM structure for N33 (D52 is not resolved in the map, Figure 3H). The JN.1 variant harbors the immune-evasive L455S mutation relative to BA.2.86 that is also compatible with the VIR-7229 paratope interface due to the small size of the introduced serine side chain, concurring with preserved binding and neutralization of BA.2.86 and JN.1 variants by VIR-7229 (Figures 1A, 1B, 1D).

The footprint of Omi-42 largely overlaps with that of VIR-7229 (24 residues are shared between VIR-7229 and Omi-42 out of 25 and 26 epitope residues, respectively, Figure S4D)^27^ and both mAbs bury a comparable surface area at the interface with the RBD. The more extensive hydrogen-bonding network of VIR-7229 with the RBD, relative to Omi-42, might explain its increased cross-reactivity and neutralization breadth (Figure 1C; S1H). VIR-7229 forms four hydrogen bonds with the backbone of residues 455, 457, and 458, whereas Omi-42 forms just one with backbone atoms in this RBD region. These results possibly explain the reduced neutralizing activity of Omi-42 for the JN.1 strain harboring the L455S mutation, and the markedly reduced neutralizing activity for XBB-descendant and JN.1-descendant variants harboring F456L^45^ (Figure S1H). Conversely, VIR-7229 neutralizes XBB-descendant F456L variants and JN.1 with high potency, and the F456L-harboring JN.1.16 variant with moderate potency (Figure 1A). Furthermore, RBD residue K458 is hydrogen-bonded via its backbone carbonyl to the VIR-7229 heavy chain Y53 side chain whereas it is the side chain of K458 that interacts with the Omi-42 heavy chain D31 and W53 side chains. Therefore, K458 mutations (observed in clades 1a and 3 sarbecoviruses) could impair interactions with Omi-42 but not with VIR-7229 and may explain the limited sarbecovirus cross-reactivity of Omi-42.

## Structural basis of VIR-7229 breadth across animal sarbecoviruses

Our structural data explain the broad VIR-7229-cross-reactivity with phylogenetically distinct sarbecovirus RBDs spanning all four clades (Figure 4). One part of the VIR-7229 epitope is highly conserved across sarbecoviruses and maps mainly outside of the RBM (Figure 4B, dark orange). However, the VIR-7229 epitope also comprises residues with considerable variation among sarbecovirus RBDs: some substitutions introduce residues of similar size and properties compared to those found in SARS-CoV-2 whereas other mutations introduce distinct residues that are nevertheless accommodated by VIR-7229 (Figures 1A, 1C–D, 4). RsSHC014, the most weakly neutralized bat sarbecovirus in our panel, harbors W455 which is expected to disrupt the interface with VIR-7229, as none of the energetically favored side chain rotamers at this position can be accommodated without steric hindrance with surrounding residues (Figure S4E). Furthermore, deletion of residues 473–477 in the RBD of the non-ACE2-utilizing bat clade 2 sarbecoviruses would reduce interactions with the heavy chain CDR1 and CDR3, leading to a reduction of epitope buried surface area of ~180 Å^2^, in line with the experimentally-observed dampened VIR-7229 binding (Figures 1C–D).

Collectively, our data show that although some VIR-7229 epitope residues are mutational hotspots for SARS-CoV-2 or are positions substituted in distinct sarbecoviruses, these substitutions are accommodated by VIR-7229, illustrating a high mutational tolerance of this RBM-targeting mAb.

## VIR-7229 has an unusually high barrier to viral escape

**Figure 5.**
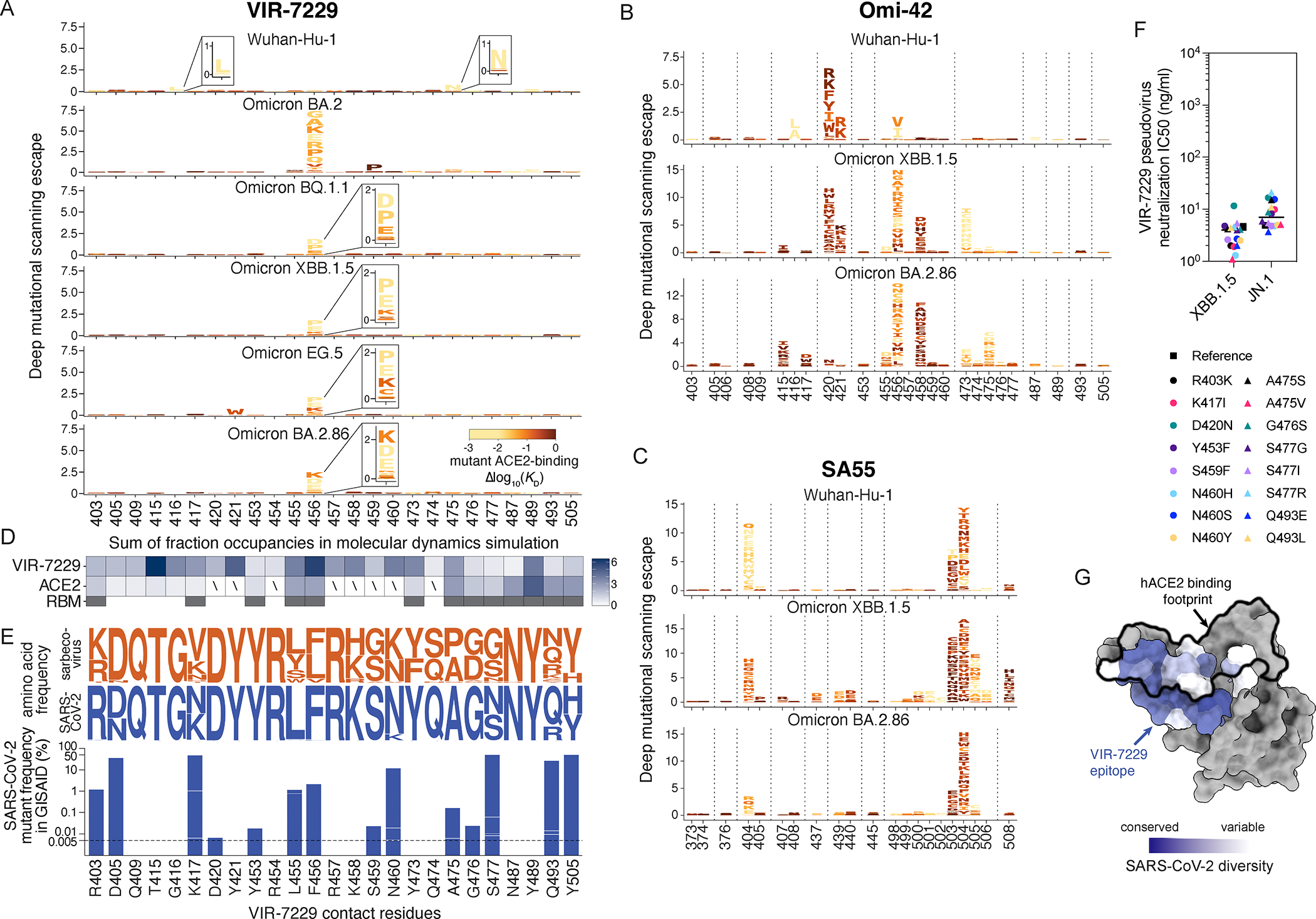
VIR-7229 has high tolerance for SARS-CoV-2 epitope variation. (A-C) Complete elucidation of mutations in the Wuhan-Hu-1, BA.2, BQ.1.1, XBB.1.5, EG.5, and BA.2.86 RBDs that enable escape from VIR-7229 (A), Omi-42 (B), or SA55 (C) binding using a yeast-display deep mutational scanning method. Letter height is proportional to mutant escape. Mutations are colored by their measured impacts on ACE2-binding affinity, where lighter yellow indicates increasingly deleterious effects on receptor binding (scale bar, bottom-right). See also Figures S5 and S6. (D) Summary of ≥4.0-μs MD simulations of XBB.1.5 RBD bound to VIR-7229 or ACE2. Boxes are the number of persistent contacts at each RBD position in the VIR-7229 epitope, expressed as the fraction occupancy for each VIR-7229 or ACE2 contact across the MD simulation, added together for each RBD position. See panels A or E for RBD position annotations. Slash indicates no contact, i.e. sum of fraction occupancy <0.1. Full glycans were modeled into the RBD:ACE2 X-ray structure; some ACE2 contacts are glycan-mediated, see Figure S4F. The third row indicates RBM residues (gray boxes), defined as RBD:ACE2 protein:protein contacts within 5 Å in the X-ray structure. See also Figure S4 and Data S4. (E) Top, logoplots illustrating the frequency of amino acid variation in VIR-7229 epitope residues across human-ACE2-utilizing sarbecovirus sequences (orange) and SARS-CoV-2 sequences available on GISAID from May 8, 2024 (blue). Bottom, barplot illustrating SARS-CoV-2 mutant frequencies (log scale; residues present in the ancestral Wuhan-Hu-1 sequence are not plotted) for all mutants with >0.005% occurrence in GISAID (up to May 8, 2024). VIR-7229 neutralization of each of these mutations was validated via neutralization of single mutants introduced into XBB.1.5 and JN.1 pseudovirus (panel F) or presence of a mutation in a circulating variant that is neutralized (Figure 1A), with the latter mutations labeled with asterisk. (F) VIR-7229-mediated neutralization of SARS-CoV-2 epitope variants with >0.005% frequency in GISAID (panel E), tested on the XBB.1.5 and JN.1 backgrounds. “Reference” refers to XBB.1.5 or JN.1 with no additional amino acid substitutions. Substitutions are annotated relative to the Wuhan-Hu-1 sequence. R403K is part of the JN.1 reference sequence. See also Data S1. (G) SARS-CoV-2 conservation of the VIR-7229 epitope mapped to SARS-CoV-2 Wuhan-Hu-1 RBD structure (PDB 6M0J). ACE2 binding footprint is illustrated as a black outline.

To investigate the potential for viral resistance to VIR-7229, we exhaustively mapped its escape profile using deep mutational scanning (DMS) of the Wuhan-Hu-1, BA.2, BQ.1.1, XBB.1.5, EG.5, and BA.2.86 yeast-displayed RBDs. VIR-7229 featured a remarkably narrow escape profile (Figures 5A, S5A–C) as compared to its parent mAb S2V29 (Figure S5C) and even more so relative to Omi-42, SA55, and S2K146 (Figures 5B–C, S5D) or other published SARS-CoV-2 mAb DMS profiles^20,21,29,53^. These results align with the sub-nanomolar binding affinity of VIR-7229 to most SARS-CoV-2 variant RBDs (Figure 1D) and our previous observation that binding affinity inversely correlates with escape profile width.^20^ Concurring with our structural analysis, VIR-7229 is largely unaffected by mutations at position K458, whereas Omi-42 binding is abrogated by several substitutions at this position in multiple backgrounds (Figure 5B). Many of these mutations correspond to residue changes found in sarbecoviruses, such as K458H found in clade 1a and clade 3 RBDs or K458S/A which is present in some clade 2 RBDs, consistent with the limited sarbecovirus breadth of Omi-42 relative to VIR-7229 despite overlapping epitopes.

Most VIR-7229 DMS escape mutations mapped to RBD position 456, which was the only position of escape for the BQ.1.1, XBB.1.5, EG.5, and BA.2.86 RBDs, with the exception of Y421W observed in the EG.5 background. All VIR-7229 DMS escape mutants reduced ACE2-binding affinity (yellow/orange letters in Figure 5A; see also Figure S6A) and those observed in the most recent variant backgrounds (BQ.1.1, XBB.1.5, EG.5, BA.2.86) require 2–3 nucleotide mutations from the wildtype codon, with the exception of P456 which is only 1 nucleotide mutation away from the recently-circulating L456 (Figure S6A). These mutations are ultra rare in SARS-CoV-2 sequenced genomes, with at most two occurrences in the GISAID database for each, likely due to the high barrier to sampling as well as to reduced fitness (Figure S6A). The importance of residue 456 for viral fitness was confirmed by a >4 μs MD simulation of the XBB.1.5 RBD:ACE2 complex, revealing that residue 456 is one of the RBM positions with which ACE2 makes multiple persistent contacts (Figures 5D, S4F; Data S4). This observation explains the large reduction of RBD:ACE2 binding affinity resulting from non-conservative substitutions at position 456 (Figures 5A, S6A). Residue 456 is also a key Omi-42 DMS escape position (Figure 5B) with many more amino acid substitutions at that position impacting binding relative to VIR-7229, including several mutations, such as F456L, which do not have a significant impact on RBD:ACE2 binding affinity (dark red letters in Figure 5B).

**Figure 6.**
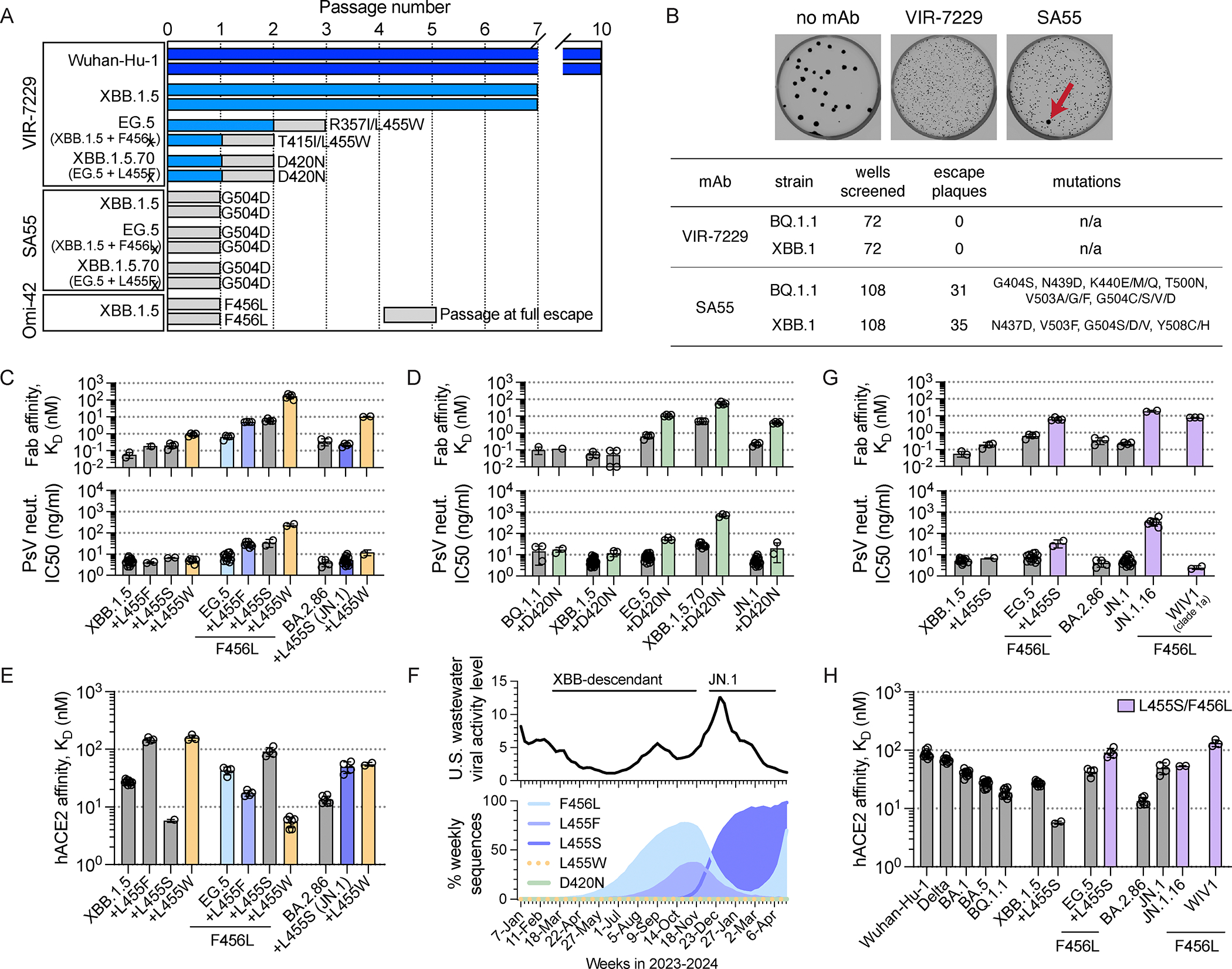
VIR-7229 exhibits a high barrier to viral escape. (A) Serial passaging of Wuhan-Hu-1 and XBB.1.5 rVSV in the presence of mAb did not result in escape from VIR-7229, as defined by ≥20% cytopathic effect in the presence of 20 μg/mL mAb (experiment terminated after 10 and 7 passages, respectively) whereas XBB.1.5 rVSV escaped from comparator mAbs (SA55 and Omi-42) after a single passage. EG.5 rVSV escaped from VIR-7229 after two to three passages, and from a comparator mAb (SA55) after a single passage. XBB.1.5.70 rVSV escaped from VIR-7229 after two passages and from a comparator mAb (SA55) after a single passage. Two independent replicates were performed for each experiment. Figure shows RBD mutations observed after sequencing; the T941K mutation was also observed in one replicate of the XBB.1.5.70 serial passaging with VIR-7229. See also Figure S6 and Data S5. (B) Plaque-based selection of BQ.1.1 and XBB.1 rVSV escapes was performed with VIR-7229 and comparator mAb SA55. Zero escape plaques were observed in 72 independent selections for VIR-7229 whereas 31 and 35 escape plaques, respectively, were observed in 108 independent selections for SA55. Representative images from BQ.1.1 selection are shown, red arrow indicates escape plaque. See also Data S5. (C) Impact of mutations at RBD position 455 on VIR-7229 Fab fragment binding affinity measured by SPR (top) and on VIR-7229-mediated pseudovirus neutralization (bottom). EG.5+L455F is XBB.1.5.70. Colored bars correspond to mutations plotted in panel F. See also Data S1. (D) Impact of the D420N mutation on VIR-7229 Fab fragment binding affinity measured by SPR (top) and on VIR-7229-mediated pseudovirus neutralization (bottom). See also Data S1. (E) Impact of mutations at RBD position 455 on ACE2 affinity measured by SPR. Colored bars as in panel C. See also Data S1. (F) Top – SARS-CoV-2 viral activity level in U.S. wastewater, January 2023 – April 2024 (cdc.gov). Bottom – Frequency of SARS-CoV-2 S mutations as percentage of weekly sequences deposited in GISAID, January 2023 – April 2024. L455W and D420N frequencies are too low to be visible. As of May 8, 2024, >96% of L455F and >86% of L455W mutations co-occur with F456L, primarily in EG.5 and derivative strains; approximately 94% of L455S mutations are in a BA.2.86/JN.1 background. (G) Impact of L455S +/− F456L on VIR-7229 Fab fragment binding affinity measured by SPR (top) and on VIR-7229-mediated pseudovirus neutralization (bottom). See also Data S1. (H) Impact of L455S +/− F456L mutations on ACE2 affinity measured by SPR. See also Data S1.

To directly evaluate viral escape from VIR-7229, we used replicating VSV (rVSV) chimeras harboring SARS-CoV-2 variant S glycoproteins instead of endogenous VSV G (Figure 6A; Data S5). These experiments were performed with VIR-7229 alongside SA55 and Omi-42 mAbs as benchmarks. A single round of passaging was sufficient to select viral escapes for the SA55 mAb (G504D in the XBB.1.5, EG.5, and XBB.1.5.70 S backgrounds) and Omi-42 (F456L in the XBB.1.5 S background) (Figure 6A); a previous study showed similar ease of escape from S2K146 via the Y489H mutation in Wuhan-Hu-1 S.^33^ In all cases, these escape mutations concur with our DMS data (Figures 5B–C, S5D). In contrast, we did not observe any VIR-7229 escape for Wuhan-Hu-1 and XBB.1.5 S backgrounds after ten and seven rounds of serial passaging, respectively. These results were consistent with orthogonal plaque-based selection assays with BQ.1.1 S and XBB.1 S rVSV, with which we selected several escape mutants for the SA55 mAb but none for VIR-7229 (Figure 6B; Data S5). We observed escape from VIR-7229 only with EG.5 S or XBB.1.5.70 S rVSV after two or three rounds of serial passaging, leading to the emergence of the L455W mutation combined with R357I or T415I (EG.5 S) or of the D420N mutation (XBB.1.5.70) (Figure 6A). The results obtained with EG.5 S concur with the reduced binding and neutralization of the RsSHC014 S pseudovirus (Figures 1A, 1C), which harbors W455 at the equivalent RBD residue position.

To validate the DMS and serial passaging results, we evaluated VIR-7229-mediated neutralization of a large panel of SARS-CoV-2 S pseudovirus mutants (Figures 5E–F, S6A–C; Data S1). All epitope substitutions which appear in the GISAID database with >0.005% frequency (as of May 8, 2024) were potently neutralized by VIR-7229 when introduced as single mutants in the XBB.1.5 and JN.1 backgrounds or when tested in the context of a circulating variant harboring that mutation (Figures 1A, 5F; Data S1), underscoring the resilience of this mAb to epitope mutations found in circulating variants (Figures 5E, 5G). We observed a complete or near-complete loss of neutralization with the G416L, F456D, F456E, F456P, F456K and F456R mutations in all S backgrounds tested (Figure S6A), all of which severely reduce ACE2 binding affinity and have a notable defect in pseudovirus infectivity (Figure S6A; Data S5; each mutation has a maximum of 2 occurrences in GISAID). The effect of several other mutations on neutralizing activity was dependent on the S background in which they were evaluated. For instance, A475N promoted full neutralization escape in the Wuhan-Hu-1 background where it creates a new glycosylation site (due to the presence of S477), but not in an Omicron background (given the S477N mutation abrogating the glycosylation sequon). S459P promoted full or partial escape from VIR-7229-mediated neutralization in early-Omicron (BA.2 and BA.5) but not in later-Omicron (BQ.1.1 and XBB.1.5) S backgrounds (Figure S6A; Data S1). These results might be explained by remodeling of the putative BA.2/BA.5 RBD-N460/VL-Y97 hydrogen bond to an RBD-K460/VH-D57 salt bridge (the latter interaction being stronger and observed in the VIR-7229-bound EG.5 structure). L455W did not promote neutralization escape in BQ.1.1, XBB.1.5, or BA.2.86/JN.1 S backgrounds, but led to reduced neutralization in BA.5 and EG.5 S backgrounds, likely due to the presence of N460 or of the F456L mutation, respectively (Figure S6B; Data S1). Consistent with the serial passaging results, either the R357I or T415I mutations in combination with L455W were required to promote complete escape from VIR-7229-mediated neutralization or binding in the EG.5 S background (neutralization IC_50_: EG.5-L455W 236 ng/ml, EG.5-L455W/R357I >1250 ng/ml, EG.5-L455W/T415I >1250 ng/ml; binding affinity: EG.5-L455W K_D_ 160 nM; Figures 6C, S6B). Although D420N modestly attenuated VIR-7229 potency in BQ.1.1, XBB.1.5, EG.5, and JN.1 S backgrounds (~2–7-fold reduction), it had a larger impact in the XBB.1.5.70 S background (neutralization IC_50_: 708 ng/ml, ~25-fold reduction; binding affinity: 50 nM; Figure 6D). This result concurs with the resistance selection experiments and is potentially explained by the additional contributions of the L455F/F456L mutations present in XBB.1.5.70 S.

The above findings point to D420N and L455W as key mutations promoting viral escape from VIR-7229 in a subset of SARS-CoV-2 S variant backgrounds. RBD residue D420 is >99.99% conserved among circulating SARS-CoV-2 isolates (based on the GISAID database as of May 8, 2024) and 100% conserved among sarbecoviruses, including divergent bat ACE2- and non-ACE2-utilizing sarbecoviruses (Figure 4; Data S1), suggesting a likely constraint for viral fitness. D420 is hydrogen-bonded to the Y369 side chain from a neighboring RBD in the closed S trimer and this interaction is conserved in SARS-CoV-1 S (clade 1a) and PRD-0038 S (clade 3) (Figure S4G). Though the D420N substitution would be compatible with this interaction, it would form a weaker hydrogen bond, possibly altering RBD opening propensity within the S trimer and modulating both ACE2 binding and exposure of the VIR-7229 epitope.

In contrast to position 420, residue 455 has mutated in recently circulating SARS-CoV-2 variants and is key for ACE2 binding (Figure 5D). We therefore assessed the impact of the L455W substitution on markers of viral fitness. Whereas the L455W substitution reduced ACE2 binding affinity (5.9-fold) of the XBB.1.5 RBD, it enhanced ACE2 binding of the EG.5 RBD (XBB.1.5 + F456L) (Figure 6E). Additionally, L455W is anticipated to be equivalently or more immune evasive than L455F or L455S;^54^ the latter two mutations have recently been associated with epidemic spread, likely driven by convergent immune pressure at the RBD positions 455 and 456 (Figure 6F). Given that these observations are at odds with the very low L455W frequency (<0.004% in all backgrounds, <0.15% in F456L background; Figure S6B), we performed a bioinformatic analysis of intra-individual SARS-CoV-2 genomic variability to determine if this could be explained by low sampling (i.e., observation) frequency of the required T to G nucleotide mutation in context of the adjacent nucleotides throughout the SARS-CoV-2 genome. Our analysis revealed an average sampling frequency of 0.0023% for any TTG to TGG mutation, required for L455W (Table S3; Data S5), which is 17-fold lower than the most frequently sampled nucleotide change that gives rise to L455F (Table S3). Nevertheless, it is sufficiently high to anticipate recurrent sampling of L455W and subsequent growth and transmission given the favorable ACE2 binding affinity conferred by this mutation in the F456L background and immune evasion at a site under high selective pressure. Therefore, the observation that L455W has remained very rare points to reduced viral fitness not only in the F456-harboring S glycoprotein background (in which it decreased ACE2 affinity) but also in the F456L S background. A possible mechanism for the fitness defect of W455 may be that this large residue would not be accommodated in a fully closed SARS-CoV-2 S trimer without some degree of structural remodeling of adjacent residues (Figure S4E).

Although VIR-7229 potently neutralized recently circulating XBB-descendant strains harboring F456L (e.g. EG.5, FL.1.5.1, and HK.3; Figures 1A, 1B), this mutation reduced neutralization potency by two orders of magnitude in an N460-harboring (BA.5) S background (Figure S6A; Data S1). N460K (present in circulating strains since BQ.1.1) is the most relevant background for assessing future potential mAb escape, as it has become fixed in SARS-CoV-2 variants, consistent with evidence of improved fitness^55^ and with its high conservation across sarbecoviruses outside of clade 1b (Figures 4A; Data S1). During revision of this manuscript, we observed that F456L also dampens VIR-7229 pseudovirus neutralization potency in the JN.1 background (JN.1.16 sub-lineage; Figure 1, Figure 6G), which was unexpected given that the L455S/F456L combination is neutralized with high potency in the EG.5 background (EG.5-L455S IC50 33.7 ng/ml compared to JN.1-F456L IC50 435 ng/ml; both harboring the L455S/F456L combination; Figure 6G) despite only one conservative substitution in the VIR-7229 epitope between these variants (R403K in JN.1 sub-lineages). Moreover, the clade 1a sarbecovirus WIV1, which also contains the L455S/F456L combination, is potently neutralized by VIR-7229 (IC50 7.8 ng/ml in VeroE6 cells; Figure 6G). Given that the VIR-7229 Fab 1:1 binding affinity is similar for these three variant RBDs (Figure 6G), these findings suggest that other properties may influence neutralization potency, such as S protein dynamics and/or ACE2 binding affinity. It is notable that, in general, mutations L455S +/− F456L are associated with reduced ACE2 binding affinity (Figure 6H), which increases the probability of reversion in future SARS-CoV-2 circulating strains. It is further notable that the growth of F456L in JN.1-descendant lineages occurred in the context of a marked decline in overall levels of circulating SARS-CoV-2 virus, as measured by viral activity in U.S. wastewater at that time (Figure 6F).

Overall, VIR-7229 exhibits a very high barrier to escape, as illustrated by extraordinarily narrow DMS profiles, the difficulty to select for escape mutations, and the fitness defects associated with mutations that lead to complete escape, which are rarely (if at all) observed among circulating SARS-CoV-2 isolates. This high barrier to escape, combined with unparalleled breadth and neutralization potency, establish VIR-7229 as a promising mAb predicted to remain active despite SARS-CoV-2 evolution.

## Discussion

Identifying epitopes resilient to viral evolution remains fundamental to the development of durable anti-viral mAbs. One strategy has been the identification of epitopes with high phylogenetic conservation as this may be predictive of future conservation if due to a functional constraint. However, sequence conservation may frequently result from low immune pressure rather than functional constraint^56^ and these epitopes may be more vulnerable than they first appear. For example, the SD1 region (residues 323–331 and 532–591) has an average conservation of 99.6% in the GISAID database, but E554K present in BA.2.86/JN.1 variants results in full escape from SD1-targeting neutralizing mAbs,^10,11^ likely impacting at least one SD1-targeting mAb in clinical development.^57^ Likewise, whereas the stem helix (residues 1139–1160) is highly conserved (>99.9% in GISAID), there appears to be little functional pressure to maintain epitope residues targeted by anti-stem helix mAbs, as escape mutants are easily selected.^22^ Therefore, prioritizing mAbs with demonstrated ability to accommodate epitope diversity may be a better strategy for long-term resilience than relying solely on evolutionary epitope conservation.

Another approach for identifying epitopes resilient to viral evolution is to have an overlap with an area of functional importance for the virus, such as the RBM, with the expectation that antigenic changes will be restrained by fitness constraints. This approach was employed for most of the SARS-CoV-2 mAbs developed at the beginning of the COVID-19 pandemic, but fell short because only a small subset of RBM residues are actually constrained by receptor binding.^9,30–32^ Consequently, all RBM-targeting mAbs developed early in the pandemic lost their ability to neutralize circulating variants.

In this study we describe the identification and characterization of VIR-7229, which neutralizes all SARS-CoV-2 variants that have arisen thus far, and which has potential durability to viral evolution. The parent mAb of VIR-7229, designated S2V29, was isolated from an individual vaccinated with Wuhan-Hu-1 S, and subsequently infected with an Omicron variant, resulting in the recall of cross-reactive memory B cells.^13,52,58^ S2V29 is endowed with high potency and cross-reactivity to all sarbecovirus clades, properties that were further improved by ML-guided affinity maturation utilizing SARS-CoV-1 RBD (which differs from the SARS-CoV-2 RBD at positions 455 and 456, among other positions; Figure 4), yielding VIR-7229. VIR-7229 is one of very few mAbs described to date capable of neutralizing all SARS-CoV-2 variants which have emerged after four and a half years of antigenic evolution, and is the only RBM-directed mAb with pan-sarbecovirus cross-reactivity. VIR-7229 has a very high barrier to viral resistance: key epitope contacts are important for ACE2 binding and therefore functionally and evolutionary constrained, a form of receptor molecular mimicry. In addition, the high tolerance for epitope diversification is promoted by its high-affinity binding (Figure 1D) and its extensive contacts with the RBD backbone, which are unchanged upon RBD residue mutations (Figure 3E). Receptor molecular mimicry has also been attributed to the P4J15 and S2K146 mAbs, though their breadth and escape resistance are more limited.^33,59^

The unique binding mode of VIR-7229, extraordinary pan-sarbecovirus breadth, and high tolerance for epitope variation, suggest that VIR-7229 may prove resilient to SARS-CoV-2 evolution. VIR-7229 could also be considered as a component of a pandemic preparedness strategy due to its neutralization of divergent bat-infecting sarbecoviruses, including strains known to be able to evolve human ACE2 binding via single amino acid changes,^39,40^ in the event of a future spillover from a zoonotic reservoir.

### Limitations of the Study

The diversity of SARS-CoV-2 variants, and the speed with which new variants emerge, have made it challenging to characterize SARS-CoV-2 mAbs. We have performed our escape profiling experiments in multiple backgrounds, creating a comprehensive picture of the remarkably narrow VIR-7229 escape profile. However, due to experiment lead times, several experiments were not performed with the current circulating strains, e.g. we do not have DMS profile or resistance selection in the JN.1 background. Additionally, at the time of our final preparation of this manuscript (June 2024) it is a period of very low SARS-CoV-2 viral circulation. It is unknown what variant will drive the next wave of SARS-CoV-2; however, based on the unprecedented breadth of VIR-7229, as well as its molecular receptor mimicry, we anticipate that VIR-7229 will continue to neutralize future variants.

## STAR METHODS

### RESOURCE AVAILABILITY

#### Lead contact

Further information and requests for resources and reagents should be directed to and will be fulfilled by the lead contact, Tyler Starr (tyler.starr@biochem.utah.edu).

#### Materials availability

SARS-CoV-2 deep mutational scanning libraries are available from Addgene: https://www.addgene.org/pooled-library/bloom-sars-cov-2-rbd-ssm/. Antibody sequences are available from the structures deposited in the Protein Data Bank (PDB). Other materials generated in this study are available from the corresponding lab after completion of a materials transfer agreement.

#### Data and code availability

Structures are available from the PDB: 9AU1 (XBB.1.5 RBD – VIR-7229 – S309); 8S6M (BQ.1.1 RBD – S2V29 – S2H97); 9ATM (EG.5 RBD – VIR-7229 – S2H97); 9ASD (BA.2.86 S – VIR-7229; EMD-43813). Sequencing data from deep mutational scanning experiments are available from the NCBI Sequence Read Archive, BioProject PRJNA714677 BioSample SAMN41715061 (breadth assays) and BioProject PRJNA770094 BioSample SAMN41694243 (DMS escape selections). Complete code and intermediate and final data files for deep mutational scanning experiments are available from GitHub: https://github.com/tstarrlab/SARSr-CoV_mAb-breadth_S2V29 and https://github.com/tstarrlab/SARS-CoV-2-RBD_Omicron_MAP_S2V29. Other raw data underlying manuscript figures is available from Supplemental Data as outlined above.

Any additional information required to reanalyze the data reported in this paper is available from the lead contact upon request.

### EXPERIMENTAL MODEL AND STUDY PARTICIPANT DETAILS

#### Human participants

Blood mononuclear cells utilized for mAb discovery were obtained from SARS-CoV-2 infected individuals under study protocols approved by the local Institutional Review Board (Canton Ticino Ethics Committee, Switzerland). All donors provided written informed consent for the use of blood and blood components (such as PBMCs, sera or plasma).

#### Cell lines

Cell lines were obtained from ATCC (HEK293T, VeroE6), Thermo Fisher Scientific (Expi293F), Invitrogen (ExpiCHO) and Takara (Lenti-X 293T). Vero-TMPRSS2 (Vero-T) cells were generated in-house.^61^ Expi293 and ExpiCHO cells were maintained in Expi293 Expression Medium (Invitrogen) and ExpiCHO Expression Medium (Gibco), respectively. VeroE6 and Lenti-X cells were cultured in DMEM high glucose with GlutaMAX (Gibco) supplemented with 10% fetal bovine serum (FBS) (Integro) and 1% Penicillin-Streptomycin (Gibco). Vero-T cells were cultured in DMEM high glucose with GlutaMAX (Gibco) supplemented with 10% fetal bovine serum (FBS) (Integro), 1% Penicillin-Streptomycin (Gibco) and 8 ug/mL puromycin (Gibco). All cell lines used in this study, except Expi293 used for protein expression and HEK293T used for sarbecovirus neutralizations, were routinely tested for mycoplasma and found to be mycoplasma-free.

#### Animals

All animal experiments were performed according to the French legislation and in compliance with the European Communities Council Directives (2010/63/UE, French Law 2013–118, February 6, 2013) and according to the regulations of Institut Pasteur Animal Care Committees. The Animal Experimentation Ethics Committee (CETEA 89) of the Institut Pasteur approved this study (200023; APAFIS#25326–2020050617114340 v2) before experiments were initiated. Hamsters were housed by groups of 3–4 animals in isolators with ad libitum access to water and food. The animals were manipulated in class III safety cabinets in the Institut Pasteur animal facilities accredited by the French Ministry of Agriculture for performing experiments on live rodents. All animals were handled in strict accordance with good animal practice. Before any manipulation, animals underwent an acclimation period of one week. Male golden Syrian hamsters (Mesocricetus auratus; RjHan:AURA) of 5–6 weeks of age (average weight 60–80 grams) were purchased from Janvier Laboratories (Le Genest-Saint-Isle, France) and handled under specific pathogen-free conditions.

### METHOD DETAILS

#### Antibody isolation and recombinant production

S2V29 mAb was isolated from peripheral blood mononuclear cells (PBMC) of a SARS-CoV-2 convalescent and vaccinated individual (male, 44-year old, Caucasian) under study protocols approved by a local institutional review board (Canton Ticino Ethics Committee, Switzerland). The donor provided written informed consent for the use of blood and blood derivatives for research.

PBMC were isolated by Ficoll density gradient centrifugation and B cells were enriched by staining with CD19 PE-Cy7 (BD Bioscience, cat. 341113) and incubation with anti-PE microbeads (Miltenyi Biotec, cat. 130-048-801), followed by positive selection using LS columns (Miltenyi Biotec, cat. 130-042-401). Enriched B cells were stained with anti-IgM, anti-IgD, anti-CD14 and anti-IgA, all PE labelled, and prefusion SARS-CoV-2 S with a biotinylated AviTag conjugated to Streptavidin Alexa-Fluor 647 (Fisher scientific, cat. 10308062). SARS-CoV-2 S-specific IgG+ memory B cells were sorted by flow cytometry via gating for PE-4 negative and Alexa-Fluor 647 positive cells. Antigen-specific memory B cells were co-cultured with mesenchymal stromal cells (MSC) in the presence of a cocktail of stimuli that induces the proliferation and differentiation of B cells into antibody secreting cells. After 7 days of culture, B cell supernatants were screened for the presence of mAbs of interest.

S2V29 VH and VL sequences were obtained by RT-PCR and subcloned in IgG1 expression vectors; the amino-acid sequence of the original S2V29-VL isolated from B cells carried a germline-encoded cysteine residue which was mutated to serine to reduce the risk of forming improper disulfide bonds (the mAb carrying the Cys to Ser mutation is mAb variant S2V29-v1.2, referred to as S2V29 in this manuscript). The VH and VL amino-acid sequences of the comparator IgGs were available from previous work^15,21,33^ or retrieved from publications^27,42^ or patents^16^ and the DNA sequence was produced with codon optimization for expression in hamster cells, then subcloned into IgG1 expression plasmids. The antibodies were expressed as recombinant human IgG1 (G1m17 allotype for all, except G1m3 allotype for S2X259 and VYD222) carrying the half-life extending M428L/N434S (LS) mutation in the Fc region (except Omi-42 was produced with the M252Y/S254T/T256E [YTE] mutation in the Fc region and VYD222 was produced with LA in the Fc region). ExpiCHO cells were transiently transfected with heavy and light chain expression vectors as described previously.^15^ For in vivo experiments in Syrian hamsters, VIR-7229 and a control mAb (specific to Plasmodium falciparum sporozoite) were produced with a Syrian hamster IgG2 Fc.

For binding and ACE2 competition measurements, VIR-7229 Fab and SA55 Fab were obtained by fragmentation of the corresponding IgG using the FabLACTICA Fab kit (Genovis, Cat #: A2-AFK-025) according to manufacturer’s protocol. The Fab-containing fraction was concentrated and buffer-exchanged into filtered HBS buffer (10mM HEPES pH 7.5, 150mM NaCl) using an Amicon 10kDa cutoff concentrator (Millipore Sigma, Cat #: ACS501024). The IgG digestion reactions were analyzed by SDS-PAGE. Recombinant S309 Fab and S2X259 Fab used for the ACE2 competition experiment were expressed in HEK293 suspension cells, purified using CaptureSelect IgG-CH1 resin and buffer exchanged into PBS (ATUM Bio; Newark, CA). Recombinant S2K146 Fab used for the ACE2 competition experiment was expressed in ExpiCHO and purified using CaptureSelect CH1-XL MiniChrom columns (Thermo Fisher Scientific).

Recombinant Fabs for crystallography were produced by ATUM Bio (Newark, CA). Engineered Fabs have been previously reported to improve crystallization by rigidifying the Fab elbow hinge^62^ and by replacing the human kappa constant domain FG loop (HQGLSSP) with a shorter rabbit kappa loop (QGTTS).^63^ These designs were incorporated into VIR-7229 Fab and S309 Fab, resulting in VIR-7229^E^ Fab and S309^RK^ Fab, respectively.

#### Machine-learning-assisted affinity maturation

S2V29 was affinity matured using the following approach: (1) training libraries comprising 10^6^ to 10^7^ S2V29 variants were designed; (2) cell surface display and FACS were used to separate the training libraries by their relative affinities to SARS-CoV-2 BQ.1.1 and SARS-CoV-1 RBDs and next-generation sequencing (NGS) was used to determine the sequences of the different populations; (3) these first-round data were used to train a variety of AI/ML models (see below) to predict binding affinity of mAb variants towards the RBDs of SARS-CoV-1 and SARS-CoV-2 BQ.1.1; (4) the model predictions guided the design of an optimized 8 × 10^6^ library which was enriched through serial rounds of FACS for improved binders; (5) the second-round data provided further training for the AI/ML models, which then helped to select a set of ~50 candidate mAbs which were produced as purified protein; and (6) the ~50 candidate mAbs were evaluated in vitro for neutralization of a panel of SARS-CoV-2 pseudoviruses as well as SARS-CoV, and also for binding to a panel of sarbecovirus RBDs. The steps are similar to previously-described experimentally-driven ML approaches.^35–37^ Further details on each step are provided below.

##### First-round library design

No high-resolution structural information on the interaction between S2V29 and its epitope was available during the affinity maturation campaign. Therefore, two parallel approaches were taken for the design of the first-round library: (a) libraries with 1 × 10^7^ mAb sequence variants mutated all possible CDR positions to determine positions that when mutated yielded a range of effects on binding, and (b) additional training libraries were designed focusing on mutations to optimize thermodynamic stability of a homology-model and structure-based design using TRIAD^64^, a physics-based computational design suite that incorporates the Rosetta,^65^ Dreiding,^66^ and Phoenix force-fields^64^. mAb variants in the libraries harbored up to eight mutations each.

##### Evaluation of mAb libraries by cell surface display and FACS

The coding sequences of the S2V29 VH and VL domains were subcloned into a yeast vector for cell surface Fab display, with DNA encoding a V5 epitope tag fused in-frame to the CL domain. The libraries were constructed by PCR-amplifying the CDRs with DNA oligonucleotides containing degenerate codons and reassembling the vector *in vitro* with either Golden Gate Assembly,^67^ Gibson Assembly,^68^ or overlap extension PCR, as appropriate for each library design. Each library was transformed into *S. cerevisiae*.^69^ After expressing Fabs, cells were washed with PBS containing 0.1% BSA and incubated in the same buffer containing mouse anti-V5 antibody (SV5-Pk1, Bio-Rad Laboratories, Inc.) and either 1 μM of biotinylated SARS-CoV-1 RBD or 100 nM of biotinylated SARS-CoV-2 BQ.1.1 RBD. Cells were washed and stained with PE-streptavidin (Jackson ImmunoResearch, Inc.) and goat anti-mouse StarBright Blue 700 (Bio-Rad Laboratories, Inc.), and then subject to FACS (WOLF Cell Sorter, NanoCellect Biomedical, Inc.). Cells were binned according to levels of Fab display and antigen binding, and antibody coding sequences were sequenced using a MiniSeq (Illumina, Inc.) or a MinION (Oxford Nanopore Technologies, plc).

##### Training of AI/ML models and second-round screening

Data from the first-round library screening was used to train a variety of AI/ML models to predict binding affinity of mAb variants towards the RBDs of SARS-CoV-1 and SARS-CoV-2 BQ.1.1. Several types of algorithms were used, including logistic regression, neural networks, support-vector machines, and decision trees (implemented with one-hot encoding in Scikit-learn).^70^ When generating models, 20% of the data was withheld as a final test set, and hyperparameters were tuned using 5-fold cross-validation with the training set. The predictions of these models were manually examined while considering the relative importance of particular features, to identify potentially beneficial sets of mutations. These mutations were balanced against the complexity of library construction, leading to the design of an optimized 8 × 10^6^ combinatorial library, which contained antibody variants harboring up to 15 mutations each that were expected to bind RBDs from both SARS-CoV-2 and SARS-CoV with greater affinity than the parent mAb S2V29. Using serial rounds of FACS, as described above, the optimized library was enriched for variants that bind to the SARS-CoV-1 RBD, and the enriched populations were subsequently screened to ensure retention of binding to the SARS-CoV-2 BQ.1.1 RBD. Enriched populations from each round were deep-sequenced, and used to develop a second round of ML models distinguishing variants that bound in the most stringent conditions from less stringent conditions. The updated ML prediction scores and antibody variant abundance following FACS were used to select candidate antibodies for expression and purification from mammalian cells and downstream evaluation. Fifty-six clones were tested in a panel of in vitro neutralization and binding assays to determine the top mAb variants.

#### Recombinant RBDs, BA.2.86 S ectodomain, and ACE2 production

SARS-CoV-2 RBD proteins (residues 328–531 of S protein from GenBank NC_045512.2, modified as needed with mutations from other SARS-CoV-2 strains, with N-terminal signal peptide from mouse Ig heavy chain and C-terminal 8xHis-AviTag or Thrombin-8xHis-AviTag) and other sarbecovirus RBD proteins for SPR binding assays (except for Khosta-2 RBD, see below) were expressed in Expi293F cells at 37°C and 8% CO_2_. See Data S1 for full sequences. Transfections were performed using the ExpiFectamine 293 Transfection Kit (Gibco). Cell culture supernatants were collected four to five days after transfection and supplemented with 10x PBS to a final concentration of 2.5x PBS (342.5 mM NaCl, 6.75 mM KCl and 29.75 mM phosphates). RBD proteins were purified by IMAC using Cobalt resin and buffer exchanged into PBS by size exclusion chromatography using a Superdex 200 Increase 10/300 GL column (Cytiva). For BLI experiments, recombinant SARS-CoV-2 Wuhan-Hu-1 RBD was purified by cobalt affinity chromatography and biotinylated using a BirA biotin-protein ligase bulk reaction kit (Avidity) followed by size-exclusion chromatography using a Superdex 200 Increase 10/300 GL column (Cytiva) into PBS.

The Khosta-2 RBD contains an N-terminal mu-phosphatase signal peptide and includes residues N_316_RFPN_319_ and C_513_KQST_516_ and a C-terminal 8xHis tag followed by an AviTag (HHHHHHHHGGSSGLNDIFEAQKIEWHE). The Khosta-2 RBD was expressed in Expi293F cells (Thermo) at 37°C and 8% CO_2_. Cells were transfected with the corresponding plasmid using Expifectamine (Thermo) following the manufacturer’s protocol. Four to five days post-transfection, supernatant was clarified by centrifugation at 4,121g for 30 minutes, supplemented with 25 mM phosphate pH 8.0, and 300 mM NaCl. Supernatant was then bound to a 1 mL His trap HP or Ni Excel Resin (Cytiva) previously equilibrated in 25 mM phosphate pH 8.0, 300 mM NaCl. Affinity resins were washed with 25 mM phosphate pH 8.0, 300 mM NaCl, and 40mM imidazole prior to elution with 25 mM phosphate pH 8.0, 300 mM NaCl and 300 to 500 mM imidazole. The RBD was concentrated and purified further on a Superdex 200 Increase 10/300 size-exclusion column (Cytiva) equilibrated in 50 mM Tris pH 8.0 and 150 mM NaCl. Fractions containing monomeric and monodisperse RBDs were flash frozen and stored at −80°C.

XBB.1.5, BQ.1.1, and EG.5 RBD constructs used for crystallization included a C-terminal 8xHis-Avi, Thrombin-TwinStrep-8xHis, or Thrombin-8xHisTag, respectively. Proteins were expressed similarly as XBB.1.5, BQ.1.1, and EG.5 RBD constructs used for SPR binding assays, as described above, except with the addition of 10 μM kifunensine. Cell culture supernatant was collected four days after transfection and supplemented with 10x PBS to a final concentration of 2.5x PBS. Protein was purified using a HisTALON Superflow cartridge (Takara) followed by buffer exchange into PBS using a Superdex 200 Increase 10/300 GL column (Cytiva).

The SARS-CoV-2 BA.2.86 Hexapro S ectodomain construct harbors its native signal peptide, BA.2.86 mutations (T19I, R21T, L24-, P25-, P26-, A27S, S50L, H69-, V70-, V127F, G142D, Y144-, F157S, R158G, N211-, L212I, V213G, L216F, H245N, A264D, I332V, G339H, K356T, S371F, S373P, S375F, T376A, R403K, D405N, R408S, K417N, N440K, V445H, G446S, N450D, L452W, N460K, S477N, T478K, N481K, V483-, E484K, F486P, R493Q, Q498R, N501Y, Y505H, E554K, A570V, D614G, P621S, H655Y, I670G, N679K, P681R, N764K, D796Y, S939F, Q954H, N969K, P1143L), Hexapro mutations (F817P, A892P, A899, A942P, K986P, V987P),^71^ a mutated furin cleavage site (_682_RRARSV_687_ to _682_GSASSV_687_), and a C-terminal foldon followed by an AviTag and an 8xHis tag. The SARS-CoV-2 BA.86 S ectodomain was expressed in Expi293F cells (Thermo) incubated at 37°C and 8% CO_2_. Cells were transfected using Expifectamine293 (Thermo) following the manufacturer’s protocol. Four days post-transfection, Expi293F cell supernatant was clarified by centrifugation at 4,121g for 30 minutes, supplemented with 25 mM phosphate pH 8.0, 300 mM NaCl. The supernatant was then passed over an His-Trap Excel column (Cytiva) previously equilibrated in 25 mM phosphate pH 8.0, 300 mM NaCl and washed with 20–40 mL of buffer containing 25 mM phosphate pH 8.0, 300 mM NaCl, and 40mM Imidazole. S protein was eluted using 25 mM phosphate pH 8.0, 300 mM NaCl, and 300mM imidazole prior to being buffer exchanged to 50 mM Tris-HCl pH 8.0, 150 mM NaCl using a centrifugal filter device with a MWCO of 100 kDa. The S glycoprotein was subsequently run over a Superose 6 Increase 10/300 size-exclusion chromatography column (Cytiva) equilibrated in 50 mM Tris pH 8.0 and 150 mM NaCl and the fractions containing monodisperse prefusion trimers were flash frozen and stored at −80°C.

For SPR binding measurements, recombinant human ACE2 (residues 19–615 from Uniprot Q9BYF1 with a C-terminal thrombin cleavage site-TwinStrep-10xHis-GGG-tag, and N-terminal signal peptide) was expressed in Expi293F cells at 37°C and 8% CO_2_. Transfection was performed using the ExpiFectamine 293 Transfection Kit (Thermo Fisher Scientific). Cell culture supernatant was collected six days after transfection, adjusted to a final concentration of 80 mM Tris-HCl pH 8.0, 100 mM NaCl, and then incubated with BioLock solution (IBA GmbH). ACE2 was purified using a StrepTrap HP column (Cytiva) followed by isolation of monomeric ACE2 by size exclusion chromatography using a Superdex 200 Increase 10/300 GL column (Cytiva) pre-equilibrated in 20 mM Tris-HCl pH 7.5, 150 mM NaCl. Recombinant ACE2 used for BLI competition experiments (residues 19–615 from Uniprot Q9BYF1 with a C-terminal Avi-10xHis-GGG-tag, and N-terminal signal peptide) was expressed in Expi293F cells as described above and purified using a HisTrap excel column followed by buffer exchange using a Superdex 200 Increase 10/300 GL column (Cytiva) pre-equilibrated in PBS.

#### Production of VSV-based SARS-CoV-2 (and SARS-CoV-1 Urbani and WIV1) S pseudotyped virus

To generate SARS-CoV-2 and Clade 1a S pseudotyped vesicular stomatitis virus (VSV) for use in neutralization assays employing VeroE6 cells, Lenti-X 293T cells were seeded in 10-cm dishes. The next day, cells were transfected with the plasmid encoding for the SARS-CoV-2 spike variant (or SARS-CoV-1 Urbani) using TransIT-Lenti (Mirus Bio) according to the manufacturer’s instructions. One day post-transfection, cells were infected with VSV(G*ΔG-luciferase) (Kerafast) at an MOI of 3–10 infectious units/cell. Viral inoculum was washed off after one hour and cells were incubated for another day at 37°C. The cell supernatant containing S pseudotyped VSV was collected at day 2 post-transfection, centrifuged at 1,000 × g for 5 minutes to remove cellular debris, aliquoted, and frozen at −80 °C.

#### VSV-based sarbecovirus (clade 1b, clade 1a, and clade 2) S pseudotyped virus production

To generate sarbecovirus S pseudotyped VSV for use in neutralization assays performed in HEK-293T-hACE2 and HEK-293T-R.alc.ACE2, constructs for membrane-anchored S glycoproteins from SARS-CoV-1 Urbani, BA.2.86 (WPL86459.1), GX-Pangolin (QIA48623.1), Khosta-1 (QVN46559.1), Khosta-2 (QVN46569.1), SARS-CoV-1 Civet007 (AAU04646.1), RaTG13delta21 (QHR63300.2), WIV1 (AGZ48828.1), RsSHC014 (AGZ48806.1), PRD-0038 (QTJ30153.1), PRD-0038-dm (harboring mutations of the SARS-CoV-2 equivalent positions K493Y/T498W) (QTJ30153.1),^39,40^ and BtKY72 (APO40579.1) were codon optimized and synthesized by Genscript for mammalian cell expression, cloned in frame with a Kozak sequence to direct translation and harboring wild-type signal peptides. The last 21 residues were deleted,^72^ except for SARS-CoV-1 Urbani, Civet007, GX-Pangolin, and WIV1. Khosta-1, Khosta-2, RaTG13, RsSHC014, PRD-0038, PRD-0038-dm (K493Y/T498W) S genes were synthesized with a triple Flag tag while the rest of the genes were synthesized with no tag. All the S genes were cloned into the HDM vector^73^ except for WIV1 S and SARS-CoV-1 Urbani S which were cloned into pcDNA3.1(−) and for GX-Pangolin S which was cloned into phCMV1.

Sarbecovirus S pseudotyped VSV were generated as previously described.^21^ Briefly, HEK293T cells in DMEM supplemented with 10% FBS and 1% PenStrep and seeded in poly-D-lysine coated 10-cm dishes were transfected with a mixture of 24 μg of the corresponding plasmid encoding for: SARS-CoV-1 Urbani S, BA.2.86 S, WIV1 S, RaTG13 S, SARS-CoV-1 Civet007 S, Khosta-1, Khosta-2 S, GX-Pangolin S, RsSHC014 S, PRD-0038 S S, PRD-0038-dm S or BtKY72 S and 60 μl Lipofectamine 2000 (Life Technologies) in 3 ml of Opti-MEM, following manufacturer’s instructions. After 5 h at 37°C, DMEM supplemented with 20% FBS and 1% PenStrep was added. The next day, cells were washed three times with DMEM and were transduced with VSVΔG-luc.^74^ After 2 h, virus inoculum was removed and cells were washed five times with DMEM prior to the addition of DMEM supplemented with anti-VSV-G antibody [Il-mouse hybridoma supernatant diluted 1 to 25 (v/v), from CRL-2700, ATCC] to minimize parental background. After 18–24 h, supernatants containing pseudotyped VSV were harvested, centrifuged at 2,000 × g for 5 minutes to remove cellular debris, filtered with a 0.45 μm membrane, concentrated 10 times using a 30 kDa cut off membrane (Amicon), aliquoted, and frozen at −80°C until use.

#### Neutralization of SARS-CoV-2 (and SARS-CoV-1 Urbani and WIV1) pseudoviruses

For neutralization of VSV-based SARS-CoV-2 (and SARS-CoV-1 Urbani and WIV1) S pseudotyped viruses, Vero E6 cells were seeded into 96-well plates at 20,000 cells/well and cultured overnight at 37°C. The next day, 10-point 3-fold or 9-point 4-fold serial dilutions of mAbs were prepared in media. SARS-CoV-2 (or SARS-CoV-1 Urbani or WIV1) pseudotyped VSVs were diluted at 0.05 or 0.1 MOI in media and added 1:1 to each mAb dilution. Virus:mAb mixtures were incubated for 1 hour at 37 °C. Media was removed from the Vero E6 cells and 50 μL of virus:mAb mixtures were added to the cells. One hour post-infection, 100 μL medium was added to all wells. After 20–24 hours incubation at 37 °C, medium was removed and 50–100 μL of BioGlo or Steadylite plus reagent (diluted 2-fold in DPBS) was added to each well. The plates were incubated at room temperature for 10–15 minutes and luminescence was read on a plate reader. Two to three technical replicates were measured and at least two biological repeats were performed. For most neutralizations performed with the S2V29 mAb and for some VIR-7229 neutralizations against single-site point mutations (but not VIR-7229 neutralizations against SARS-CoV-2 strains), experiments were performed with the addition of 100 ng/ml anti-VSV-G Ab. All data were normalized based on internal control RLU values (untreated cells for 100% neutralization and infected cells with or without anti-VSV-G antibody for 0% neutralization) and plotted with GraphPad Prism (version 10.1.2) using a nonlinear regression 4-parameters model.

#### Neutralization of sarbecovirus pseudoviruses

For pseudotyped VSV sarbecovirus neutralizations, HEK293T cells were transiently transfected with plasmids encoding for full-length human ACE2 or R. alcyone ACE2 following a previously described protocol.^74^ Briefly, HEK293T cells at 90% confluency and seeded in poly-D-lysine coated 10-cm dishes were transfected with a mixture of 8 μg of the corresponding plasmid encoding the ACE2 ortholog and 30 μl of Lipofectamine 2000 (Life Technologies) prepared in Opti-MEM according to the manufacturer’s instructions. After 5 h at 37°C, cells were trypsinized, seeded into poly-D-lysine coated clear bottom white walled 96-well plates at 40,000 cells /well and cultured overnight at 37°C. For neutralizations, eleven 2-fold serial dilutions of SA55, S2K146, S2X259, Omi-42, S309, S2V29 or VIR-7229 IgGs were prepared in DMEM. 20 μl of the different sarbecovirus pseudotypes described above were added 1:1 (v/v) to each IgG and mixtures were incubated for 45–60 min at 37°C. After removing their media, transfected HEK293T cells were washed two times with DMEM and 40 μL of the mixture containing virus:IgG were added. Two hours later, 40 μL DMEM were added to the cells. After 17–20 h, 60 μL of One-Glo-EX substrate (Promega) were added to each well and incubated on a plate shaker in the dark. After 5–15 min incubation, plates were read on a Biotek Neo2 plate reader. (S309 neutralization of SARS-CoV-1 Urbani was performed with Vero-TMPRSS2 cells.) Measurements were made in duplicate with at least two biological replicates. Relative luciferase units were plotted and normalized in Prism (GraphPad): cells alone without pseudotyped virus were defined as 0% infection, and cells with virus only (no IgG) were defined as 100% infection.

#### Neutralization of authentic SARS-CoV-2 viruses

VeroE6 cells were seeded into flat bottom tissue culture 96-well plates at 20,000 cells/well and cultured overnight at 37°C. Twenty-four hours later, 9-point 1:4 serial dilutions of VIR-7229 were prepared in infection media (DMEM + 2% BSA) and each dilution was tested in 3–4 replicates per plate (top final assay concentration of 1.25 or 5 μg/mL). SARS-CoV-2 authentic virus stock was diluted in infection media for a final concentration of 200 plaque forming units per well (MOI 0.01). Antibody dilutions were added to virus and incubated for 30 minutes at 37°C. Media was removed from the cells, mAb-virus complexes were added, and cells were incubated at 37°C. At 18–30 hours post-infection (wild-type USA-WA1/2020 18–24h [depending on virus stock], Delta 30h, BA.1 24h, BA.2 30h, BA.5 18h, XBB.1.5 24h, XBB.1.6 30h, EG.5.1 24h, FL.1.5.1 30h, JN.1 18h), cells were fixed with 4% PFA for 30 minutes at RT, then washed 3 times with PBS to remove residual PFA. The cells were permeabilized with 100 μL of 0.25% Triton X-100 in PBS for 30 minutes at RT, followed by two washes with PBS. Cells were incubated with 50 μL of anti-SARS-CoV-2 nucleocapsid antibody (Sino Biologicals, 40143-R001) at 1:2000 for 1 hour at RT. Plates were washed three times with PBS and then incubated for 1 hour at RT with 50 μL/well of goat anti-rabbit IgG Alexa647 (Invitrogen, A-21245) secondary antibody at a final dilution of 1:1000 mixed with 2 μg/mL Hoechst dye. After washing 3 times with PBS, 200 μL of fresh PBS was added for imaging. Plates were imaged on a Cytation5 plate reader. Whole well images were acquired (12 images at 4X magnification per well) and nucleocapsid-positive cells were counted using the manufacturer’s software.

#### Affinity determination by surface plasmon resonance (SPR)

Measurements were performed using a Biacore 8K or Biacore T200 instrument. Experiments were performed at 25°C, with the samples held at 15°C in the instrument prior to injection. CM5 chips with covalently immobilized anti-AviTag polyclonal antibody (GenScript, Cat #: A00674–40) were used to capture His-AviTag-containing RBDs. Running buffer was 1x HBS-EP+ pH 7.4 (10 mM HEPES, 150 mM NaCl, 3 mM EDTA and 0.05% v/v Surfactant P20) (Cytiva, Cat #: BR100669). Experiments were performed with a 3-point or 4-point dilution series of VIR-7229 Fab starting at 50 nM (50, 12.5, 3.13 nM; 50, 12.5, 3.13, 0.78 nM; or 50, 10.64, 2.26 nM) or monomeric TwinStrep-His-tagged ACE2 starting at 300nM (300, 75, 18.75 nM). The regeneration solution was 75 mM phosphoric acid.

Experiments were run as single-cycle kinetics with at least 2 replicates for each RBD ligand. Data were double reference-subtracted and fit to a binding model using the Biacore Insight software. The 1:1 binding model was used to determine the kinetic parameters. K_D_, k_a_, and k_d_ are reported in Data S1 as the average of all replicates with the corresponding standard deviation. For VIR-7229 Fab binding to Shaanxi2011 RBD, a constant R_max_ calculated from the RBD capture level was applied to account for the low R_max_ from the default analysis. For VIR-7229 Fab binding to EG.5+L455W RBD, data were analyzed with a heterogenous ligand binding model with one of the two initial dissociation rate constant values set to 1E-6 (1/s) to account for the biphasic shape of the sensorgrams. The kinetics parameters evaluated for the binding phase with a faster dissociation rate were reported as the “apparent” kinetics values for the binding interaction

#### Competition of Fab fragments and ACE2 for binding to RBD by biolayer interferometry (BLI)

Protein reagents were diluted in Kinetics Buffer 10X (Sartorius, Cat #:18–1105). The experiment was performed on an Octet Red 96 instrument. Streptavidin biosensors (Sartorius, Cat #: 18–5019) were hydrated in water for 15 min before the experiment. Biotinylated His-Avi-tagged Wuhan (Wu-WT) RBD was immobilized on the sensors at 10 μg/mL for 10 s. RBD-immobilized sensors were then dipped into Kinetics Buffer 10X for 60s to establish a baseline before being dipped into a 100 nM Fab solution for 600 s (association phase 1) and subsequently into a mixture of 100 nM of the same Fab plus 300 nM ACE2 solution for 600 s (association phase 2).

#### Cell-surface mAb-mediated S_1_ shedding

ExpiCHO cells were transfected with a plasmid encoding the Wuhan-Hu-1 or Omicron XBB.1.5 spike protein using the ExpiFectamine CHO transfection reagent according to the manufacturer’s instructions, and were cultured in growth medium at 37°C on an orbital shaker platform for 48 hours. The day of the assay, cells were collected, pelleted at 400 ×*g* for 5 minutes at 4°C, and washed once with FACS buffer (1X PBS supplemented with 0.01% BSA). Cells were centrifuged at 400 ×*g* for 5 minutes at 4°C and resuspended in FACS buffer, counted, and plated at a density of 9×10^4^ cells/well in a 96-well round bottom plate. Cells were then stained with either VIR-7229, S2M28-LS (negative control), or S2K146-LS (positive control) antibody at a final concentration of 15 μg/mL and incubated at 37°C for 5, 30, 60, 120, or 180 minutes. Cells were then washed 2 times with ice-cold FACS buffer, pelleted and resuspended in FACS buffer, and stained with 1.5 μg/ml goat anti-human IgG AlexaFluor647 secondary antibody for 20 minutes at 4°C. Cells were washed two times with ice-cold FACS buffer, pelleted and resuspended in 50 μL ice-cold FACS buffer, and samples were immediately acquired on a ZE5 flow cytometer (Bio-Rad). Controls stained with secondary antibody only were included in all experiments.

Mean fluorescence intensity (MFI) of IgG positive cells was determined using FlowJo software (v10.1.0, Becton Dickinson). Cells were selected using forward scatter-height (FSC-H) and side scatter-height (SSC-H), and doublets were removed by bivariate plot forward scatter-area (FSC-A) vs FSC-H. The IgG positive cells were identified as positive for AlexaFluor647 fluorescence. Controls stained with only secondary antibody were included in all experiments to account for non-specific binding of the secondary antibody and to gate the IgG+ cell population. MFI data were exported from FlowJo and analyzed in Excel. For each time point, percent binding relative to baseline (5 minute time point) was calculated using the MFI value of the IgG positive cell population. Percent binding data were graphed using GraphPad Prism software (v10.2.3).

#### Determination of mAb-Dependent Activation of Human FcγRIIa and FcγRIIIa

Activation of human FcγRIIa (allele H131) and FcγRIIIa (high-affinity binding allele V158) was tested using validated, commercially available bioreporter assays. CHO cells stably expressing SARS-CoV-2 Wuhan-Hu-1 spike protein (CHO-CoV-2-Spike) were used as the target antigen. Nine-point serial dilutions of mAbs (5,000 ng/ml to 0.076 ng/ml) were incubated with 12,500 (for FcγRIIIa) or 10,000 (for FcγRIIa) CHO-CoV-2-Spike cells per well in a 96-well white, flat-bottom plate for 25 minutes at room temperature. Jurkat effector cells (Promega; Cat. Nr.: G7018 and G9995) stably expressing the indicated FcγR and an NFAT-induced luciferase gene were thawed, diluted in assay buffer, and added to the plate at an effector to target cell ratio of 6:1 for FcRγIIIa or 5:1 for FcγIIa. Control wells were also included that were used to measure antibody-independent activation (containing target cells and effector cells but no antibody) and background luminescence of the plate (wells containing assay buffer only). Plates were incubated for 20 hours at 37°C with 5% CO2. Activation of human FcγRs in this bioassay results in the NFAT-mediated expression of the luciferase reporter gene. Luminescence was measured with a Synergy 2 SL luminometer (Bio-Tek) after adding the Bio-Glo^™^ Luciferase Assay Reagent according to the manufacturer’s instructions.

#### Determination of NK-Cell Mediated Antibody-Dependent Cellular Cytotoxicity

NK cells were freshly isolated from whole EDTA blood using the MACSxpress NK isolation Kit (Miltenyi Biotec, 130-098-185) following the manufacturer’s instructions. Briefly, anticoagulated blood was mixed in a 50 mL tube with 15 mL of the NK isolation cocktail and incubated for 5 minutes at room temperature using a rotator at approximately 12 round per minute. The tube was then placed in the magnetic field of the MACSxpress Separator for 15 minutes. The magnetically labeled non-target cells adhere to the wall of the tube while the aggregated erythrocytes sediment at the bottom. The (unlabeled) target NK cells were then collected from the supernatant while the tube remained inside the MACSxpress Separator. NK cells were centrifuged, treated with distilled water to remove residual erythrocytes, centrifuged again and finally resuspended in AIM-V medium. Cells from blood donors were genotyped for the FcγRIIIa F/V158 allele (SNP ID rs396991) using TaqMan SNP Genotyping Assay kit (ThermoFisher). Primary human NK cells from donors expressing homozygous high affinity (V/V158) or heterozygous (F/V158) FcγRIIIa alleles were used in the ADCC assay.

Determination of ADCC activity was performed using the highly sensitive HiBiT target cell killing bioassay (Promega). SARS-CoV-2 Wuhan-Hu-1 S CHO-K1 cells (HaloTag-HiBiT) were thawed and seeded at a density of 3,000 cells per well in a white polypropylene 96 well round-bottom plate and incubated overnight at 37°C. The next day, serial dilutions of mAbs (serially diluted 5-fold in AIM-V Medium from 20,000 ng/ml to 0.26 ng/ml) were added to the plated cells, with each dilution tested in a single replicate per NK cell donor. Wells without antibody or containing Digitonin (Sigma-Aldrich; Cat. Nr.: D141) at 100 ug/ml were used as negative and positive controls, respectively. Target cell and antibody mixtures were then incubated with primary human NK cells as effectors at an effector-to-target ratio of 10:1 (30,000 cells/well) and the plate was incubated at 37°C for 4 hours. In this assay, ADCC activity was assessed by measuring the release of HaloTag-HiBiT protein from target cell lysis using the Nano-Glo HiBiT Extracellular Detection reagent (Promega) and luminescence as a readout, according to the manufacturer’s instructions. In brief, 70 μl/well of detection reagent was added and the plate was incubated in the dark for 10 minutes and luminescence was then measured with a Synergy 2 SL luminometer. Starting from the average RLU, specific lysis was calculated using Digitonin as 100% specific lysis and the wells without antibody as 0% specific lysis. The percent specific lysis was determined by applying the following formula: (measured RLU – no antibody negative control RLU) / (digitonin positive control RLU – no antibody negative control RLU) × 100. Data were plotted in GraphPad Prism software (v10.0).

#### Evaluation of sarbecovirus cross-reactivity via high-throughput yeast-display binding assays

The complete pipeline for measuring mAb breadth across the pan-sarbecovirus panel is described at: https://github.com/tstarrlab/SARSr-CoV_mAb-breadth_S2V29/blob/main/results/summary/summary.md

MAb binding via high-throughput FACS-seq was evaluated against a previously published pan-sarbecovirus panel of yeast-displayed RBDs^39,40^ in the AWY101 yeast strain^75^ that was supplemented with additional newly described sarbecovirus and SARS-CoV-2 variants (SARS-CoV-2 Omicron BA.1, BA.2, BA.5, BQ.1.1, and XBB.1.5; RhGB03^76^; RhGB02–230^77^; Rc-kw8, Rc-os20, and Rc-mk2^41^; and BtSY1-RtLS01 and BtSY2-RmCX02^78^).

The yeast-display RBD library was grown, induced for yeast-surface expression, and labeled with monoclonal antibody at 10,000, 400, 16, 0.64, 0.0256, and 0 ng/mL concentration for one hour at room temperature. Yeast were washed with PBS-BSA and labeled with secondary Myc-FITC antibody (Immunology Consultants CMYC-45F) and PE-conjugated goat anti-human-IgG (Jackson ImmunoResearch 109-115-098). Libraries were then partitioned, at each labeling concentration, into four bins of mAb binding on a BD FACSAria, collecting a minimum of 1 million RBD^+^ cells per sample concentration across the four bins. Cells were grown post-sort, plasmid purified, N16 barcode amplified, and sequenced on an Illumina NextSeq. Raw Illumina sequencing data is available from the NCBI Sequence Read Archive, BioProject PRJNA714677, BioSample SAMN41715061. Barcode reads were mapped to library barcodes, with raw counts found at: https://github.com/tstarrlab/SARSr-CoV_mAb-breadth_S2V29/blob/main/results/counts/variant_counts.csv.

For each library barcode, an EC50 binding strength was derived from its distribution of sequence reads across sort bins. First, the strength of mAb binding to each barcode at each mAb dilution was determined as the simple mean bin from cell counts across integer-weighted bins. Any barcode with less than 2 cell counts at any single sample concentration or less than an average of 5 cell counts across all sample concentrations was eliminated from analysis. An EC50 metric was then calculated from the fit of a sigmoidal curve between mean bin (mAb binding) and mAb labeling concentration. Per-barcode EC50 calculation and representative titration curve-fits can be found at: https://github.com/tstarrlab/SARSr-CoV_mAb-breadth_S2V29/blob/main/results/summary/compute_EC50.md, and per-barcode EC50 metrics are available at: https://github.com/tstarrlab/SARSr-CoV_mAb-breadth_S2V29/blob/main/results/bc_mAb_EC50/bc_mAb_EC50.csv.

We then computed the per-variant EC50 as the robust mean of replicate barcodes linked with the identical RBD variant, by taking the mean per-barcode EC50 after trimming tails of the top and bottom 5% of EC50 values among the replicate barcodes. The final variant derivation can be found at: https://github.com/tstarrlab/SARSr-CoV_mAb-breadth_S2V29/blob/main/results/summary/collapse_barcodes_lib61_SARSr-wts.md, and final per-variant mAb-binding values are available at: https://github.com/tstarrlab/SARSr-CoV_mAb-breadth_S2V29/blob/main/results/final_variant_scores/final_variant_scores_lib61.csv

#### Evaluation of escape mutants via yeast-display deep mutational scanning

Deep mutational scanning libraries for SARS-CoV-2 variants Wuhan-Hu-1, Omicron BA.2, Omicron BQ.1.1, and Omicron XBB.1.5 were described in prior publications, including library construction, library availability, and measurements of mutational impacts on RBD expression and ACE2-binding affinity.^53,60,79^ New deep mutational scanning libraries for SARS-CoV-2 variants EG.5 and BA.2.86 were constructed and assayed for mutational impacts on expression and ACE2-binding affinity per these prior methods, and are available from GitHub in advance of future publication: https://github.com/tstarrlab/SARS-CoV-2-RBD_DMS_Omicron-EG5-FLip-BA286. These libraries consist of virtually all single amino acid changes in each RBD background in a yeast-surface display platform.

Duplicate yeast-display deep mutational scanning libraries were induced for RBD expression, and 5 OD*mL of yeast were incubated in 1 mL for one hour at room temperature with a concentration of mAb corresponding to the EC90 of the mAb for the respective yeast-displayed wildtype RBD determined from pilot isogenic binding assays. In parallel, for FACS gate setting, 0.5 OD*mL of the respective wildtype parental constructs were incubated in 100 μL of antibody at the matched EC90 concentration or 1/10 the EC90 concentration. Cells were washed, incubated with 1:100 FITC-conjugated chicken anti-Myc antibody to label RBD expression and 1:200 PE-conjugated goat anti-human IgG to label bound antibody, and washed in preparation for FACS.

Antibody-escape cells in each library were selected via FACS on a BD FACSAria II or Cytek Aurora Cell Sorter. FACS selection gates were drawn to capture approximately 50% of yeast expressing the parental RBD control labeled at the 10x reduced antibody labeling concentration (see representative gating scheme in Figure S5A). For each sample, 4 million RBD+ cells were processed on the sorter with collection of cells in the antibody-escape bin, which were expanded overnight, plasmid purified, and barcodes sequenced on an Illumina NextSeq. In parallel, plasmid samples were purified from 30 OD*mL of pre-sorted library culture and sequenced to establish pre-selection barcode frequencies. Barcode reads are available on the NCBI SRA, BioProject PRJNA770094, BioSample SAMN41694243.

Demultiplexed Illumina barcode reads were matched to library barcodes and associated RBD mutant from previously assembled barcode-variant lookup tables using dms_variants (version 0.8.9), yielding a table of counts of each barcode in each pre- and post-sort population, available at: https://github.com/tstarrlab/SARS-CoV-2-RBD_Omicron_MAP_S2V29/tree/main/results/counts.

The escape fraction of each barcoded variant was computed from sequencing counts in the pre-sort and antibody-escape populations via the formula:

$$E_{v}=F\times \frac{\left(\frac{n_{v}^{post}}{N_{post}}\right)}{\left(\frac{n_{v}^{pre}}{N_{pre}}\right)}$$

where F is the total fraction of the library that escapes antibody binding (e.g. annotated numbers in Fig. S4A), $n_{v}$ is the counts of variant v in the pre- or post-sort samples with a pseudocount addition of 0.5, and N is the total sequencing count across all variants pre- or post-sort. These escape fractions represent the estimated fraction of cells expressing a particular variant that fall in the escape bin, which scales from 0 for a mutation that never causes sufficient loss of binding to drive cells into the antibody-escape bin, to 1 for a mutation that escapes binding >10-fold such that the variant falls into the antibody-escape bin defined by the control labeling gates. We applied computational filters to remove mutants with low pre-selection sequencing counts or highly deleterious mutations that escape antibody binding artefactually due to poor RBD surface expression, specifically mutants with orthogonally measured ACE2-binding impacts of <−3 (1000fold loss of ACE2 binding) or expression scores of <−1.25, <−0.955, <−1.229, and <−1.25 for Wuhan-Hu-1, BA.2, BQ.1.1, and XBB.1.5, respectively, accounting for the variation in baseline expression levels of different wildtype variants. Final per-mutant escape fractions were computed as the average across barcodes within replicates, with the correlation between replicate library selections shown in Fig. S5B. Final escape fraction measurements averaged across replicates are available at: https://github.com/tstarrlab/SARS-CoV-2-RBD_Omicron_MAP_S2V29/tree/main/results/supp_data.

#### Polyreactivity to Hep2 cells

Assessment of polyreactivity was performed using HEp-20–10 (Euroimmun 1522–2010), an FDA-approved immunofluorescence test for reliable antinuclear antibody screening. Test and control antibodies were diluted in PBS-Tween to a final concentration of 50 μg/mL and 25 μl of the antibody solution was applied on the test slide as described by the manufacturer’s instructions. After 1h incubation at room temperature the test slide was washed 5 min in PBS-tween. Alexa488 anti hu IgG Fc gamma specific (109-545-098) was prepared at 3 μg/ml in PBS-Tween and 20 μl of the secondary antibody solution was added to the slides. After 1h-incubation at room temperature the slide was washed again for 5 min with PBS-tween, excess washing solution was removed and 10 μl glycerol/biochip was added to mount coverslip. Immunofluorescence images were acquired using the Automated Imaging Microplate Reader Cytation5 (Biotek).

Two antibodies previously extensively characterized for their unspecific binding profiles were used as positive and negative controls. An anti influenza A haemagglutinin antibody (FI6) was used as a positive control, while as a negative control an antibody targeting respiratory syncytial virus (RSV) F protein (MPE8) was used; the anti-RSV MPE8 has previously been tested on a large panel of human tissues and was shown not to display any significant non-specific binding.

#### Human tissue cross-reactivity

Assessments of cross-reactivity of S2V29 and VIR-7229 to a panel of human tissues (non-GLP) were performed by Labcorp at the Labcorp Early Development Laboratories in North Yorkshire, England. The relevant study numbers were 8510456 and 8520675, respectively.

To facilitate immunohistochemical detection, S2V29, VIR-7229, and the MGH2 negative control mAb were conjugated by Labcorp with AF488 using the commercial Alexa Fluor^®^ 488 Protein Labelling Kit (ThermoFisher Scientific, A10235). The test system was preparations derived from histologically normal frozen human tissues, collected from one donor of each of these 39 tissues: Adrenal, Urinary Bladder, Blood Cells, Bone Marrow, Breast, Cerebellum (Brain), Cortex (Brain), Colon, Duodenum, Endothelium, Eye, Fallopian Tube, Gastric Antrum, Gastric Body, Heart, Ileum, Kidney, Liver, Lung, Lymph Node, Oesophagus, Ovary, Pancreas, Parotid, Peripheral Nerve, Pituitary, Placenta, Prostate, Skin, Spinal Cord, Spleen, Striated Muscle, Testis, Thymus, Thyroid, Tonsil, Ureter, Cervix (Uterus), and Endometrium (Uterus). Tissues, with the exception of blood cells, were cryo-sectioned. Blood cells were prepared as smears.

To generate positive and negative controls, CHO-K1 cells (ATCC^®^ CRL-9618) previously engineered to stably express SARS-CoV-2 Spike protein on the cell surface (CHO-nCoV-2S cells) and parental CHO-K1 cells (CHO-K1 cells) were cultured for 6 days in DMEM/Ham’s F-12 supplemented with 15 mM HEPES, stable Glutamine and 10% FBS. On day 6, cells were detached with Trypsin-EDTA, gently washed, and resuspended in PBS at a concentration of 10^6^ cells/ml. Positive control cell blocks were generated as a heterogenous sample of both positive-control and negative-control cells (to ensure signal is not saturated): CHO-nCoV-2S and CHO-K1 cells were mixed at a 7:3 ratio. For negative control cell blocks, only CHO-K1 cells were used. For each cell suspension, 3×1 ml were harvested by centrifugation, the supernatant was decanted, and the cells were resuspended in the remaining volume. Finally, the cell suspensions were added to Shadon Disposable Base Mold prefilled with 1ml of Killik embedding medium (Bio-Optica, 05–9801) and mixed homogenously by gently swirling with a 1ml pipet tip. The molds were rapidly frozen by placing them on top of an acetone/dry ice bath and were immediately transferred to dry ice. The frozen molds were wrapped and store at −80°C until use.

Method development was performed by Labcorp to generate a suitable immunohistochemical staining method for use in the control titration and tissue titration. Findings for each tissue were individually graded and identified by cell type or structure where possible. In addition to grading intensity (I) and frequency (F), a text comment for each finding recorded, where possible, the staining pattern observed as either membranous, cytoplasmic or nuclear, or combinations thereof. The numerical score for intensity (I) indicates the highest intensity of positive staining observed.

#### In vivo efficacy evaluation using a Syrian golden hamster model

Male Syrian golden hamsters (Mesocricetus auratus; RjHan:AURA) of 5–6 weeks of age (average weight 60–80 grams) were purchased from Janvier Laboratories (Le Genest-Saint-Isle, France) and handled under specific pathogen-free conditions. The hamsters were treated intraperitoneally with VIR-7229-GH-rIgG2a (5 mg/kg, 1.5 mg/kg, 0.5 mg/kg, 0.17 mg/kg, or 0.06 mg/kg) or with the isotype control mAb MPE8v3-GH-rIgG2a (1.5 mg/kg), n=6 animals per group. One day later, animals were anesthetized (intraperitoneal administration of ketamine [Imalgène 200 mg/kg] and xylazine [Rompun 2 mg/kg]) and inoculated intranasally with 6×10^4^ PFU/hamster of SARS-CoV-2 Omicron/XBB.1.5 (GISAID ID: EPI_ISL_16353849, kindly provided by O. Schwartz and colleagues), with 6×10^4^ PFU/hamster of SARS-CoV-2 Omicron/JN.1 (GISAID ID: EPI_ISL_18522058, provided by the National Reference Centre for Respiratory Viruses hosted by Institut Pasteur, Paris, France) or were mock-infected (JN.1 study only, but data provided a qualitative comparison for the body weight and clinical scores in the XBB.1.5 study). Infected and mock-infected hamsters were housed in separate isolators. Body weight and clinical score were recorded daily, except for Day 1 post-infection where only clinical score was recorded. The clinical score assignment was defined by the following criteria: 1 = ruffled fur; 2 = slow movements; 3= apathy; 4 = absence of exploratory activity. On Day 4 post-infection, the lungs were collected from all groups, weighed, and processed for viral load assays.

#### Quantification of viral RNA load (RT-qPCR) for evaluation of efficacy in hamsters

Frozen lungs fragments were weighed and homogenized with 1 mL of ice-cold DMEM (31966021, Gibco) supplemented with 1% penicillin/streptomycin (15140148, Thermo Fisher) in Lysing Matrix M 2 mL tubes (116923050-CF, MP Biomedicals) using the FastPrep-24^™^ system (MP Biomedicals) and the following scheme: homogenization at 4.0 m/s for 20 s, incubation at 4°C for 2 min, and new homogenization at 4.0 m/s for 20 s. The tubes were centrifuged at 10,000 × g for 1 min at 4°C. Afterwards, 125 μL of the tissue homogenate supernatant were mixed with 375 μL of Trizol LS (10296028, Invitrogen) and the total RNA was extracted using the Direct-zol RNA MiniPrep Kit (R2052, Zymo Research). The presence of SARS-CoV-2 RNA in these samples was evaluated by one-step qRT-PCR utilizing the the SuperScript III Platinum One-Step qRT-PCR Kit (Invitrogen 11732–020) in a final volume of 12.5 μL per reaction in 384-wells PCR plates using a thermocycler (QuantStudio 6 Flex, Applied Biosystems). Briefly, 2.5 μL of RNA template was added to 10 μL of a master mix containing 6.25 μL of 2X Reaction Mix, 0.2 μL of MgSO_4_ (50 mM), 0.5 μL of SuperScript III/Platinum *Taq* Mix (2 UI/μL), and 3.05 μL of nuclease-free water containing the nCoV_IP2 primers (nCoV_IP2–12669Fw: 5’-ATGAGCTTAGTCCTGTTG-3’; nCoV_IP2–12759Rv: 5’-CTCCCTTTGTTGTGTTGT-3’) at a final concentration of 400 nM, and the nCoV_IP2 probe (5’-FAM-AGATGTCTTGTGCTGCCGGTA-3’-TAMRA) at a final concentration of 200 nM. The amplification conditions were as follows: 55°C for 20 min, 95°C for 3 min, 50 cycles of 95°C for 15 s and 58°C for 30 s, and a last step of 40°C for 30 s. Viral load quantification (expressed as RNA copy number/g of tissue) was assessed by linear regression using a standard curve of six known quantities of RNA transcripts containing the RdRp sequence (ranging from 10^7^ to 10^2^ copies). The limit of detection is 1×10^2^ viral RNA copies/μL, which was converted to RNA copy number/g of tissue considering the weight of the homogenized lung fragments and the concentration of RNA in each sample.

#### Quantification of viral titer (TCID50) for evaluation of efficacy in hamsters

Frozen lung fragments were weighed and homogenized with 1 mL of ice-cold DMEM supplemented with 1% penicillin/streptomycin (15140148, Thermo Fisher) in Lysing Matrix M 2 mL tubes (116923050-CF, MP Biomedicals) using the FastPrep-24^™^ system (MP Biomedicals), and the following scheme: homogenization at 4.0 m/s for 20 s, incubation at 4°C for 2 min, and new homogenization at 4.0 m/s for 20 s. The tubes were centrifuged at 10,000 × g for 2 min at 4°C and the supernatants collected. Supernatants were serially diluted (1:10) in DMEM supplemented with 1% penicillin/streptomycin and 1 μg/mL of Trypsin-TPCK (4370285, Sigma-Aldrich) and then 100 μL of each dilution were added in a well of a 96 well-plate in six replicates. 100 μL containing 8×10^4^ VeroE6 cells were added in each well and the plates were incubated at 37°C and 5% CO_2_ for 72 hours. The plates were then washed in PBS. For XBB.1.5 virus quantification, plates were stained with crystal violet (11778193, BD) for 15 minutes, washed again in PBS, and plaques were counted. For JN.1 virus quantification, wells that contained lysed cells and/or with cytopathic effect (fused multinucleated cells, formation of syncytia) were counted using the 4x objective of an EVOS M5000 imaging system. Viral titers were obtained by classical TCID_50_ method calculated using the TCID_50_ calculator (v2.1 – 20-01–2017_MB. available at: https://www.klinikum.uni-heidelberg.de/fileadmin/inst_hygiene/molekulare_virologie/Downloads/TCID50_calculator_v2_17-01-20_MB.xlsx). The limit of detection is 3.16×10^1^ plaque-forming units (PFU)/mL. The limit of detection expressed as TCID_50_/100 mg of tissue is not uniform: for each study, the limit of detection in PFU/100 mg lung tissue was determined based on the assay limit of detection in PFU/mL and weight of the homogenized lung fragments for samples where no virus was detected.

#### Enzyme-linked immunosorbent assay (ELISA) to determine in vivo serum titers

Ninety-six half area well-plates (Greiner 650001) were coated overnight at 4°C with 50 μL of SARS-CoV-2 Wuhan-Hu-1 RBD protein (nCoV-RBD-D-STREPH) prepared at 2 μg/mL in PBS pH 7.2. Plates were then washed three times with PBS 0.05% Tween 20 (PBS-T) and blocked with casein 1% in PBS during 2 hours. The plates were washed three times in PBS-T and 50 μL of diluted serum samples (1:10–1:20) were applied to each well, in duplicate. Standard curves were made with serial dilutions (1:3) of VIR-7229. The plates were incubated for 1 hour, washed six times with PBS-T and incubated again with an AP-labeled anti-hamster IgG (0.5 μg/mL) for 1 hour in the dark. Plates were then washed six times with PBS-T and 4-nitrophenyl phosphate substrate (pNPP, Sigma-Aldrich, 71768) was added. After 45 min incubation, absorbance at 405 nm was measured by a plate reader (Victor Nivo, Perkin Elmer).

#### Crystallization, data collection, structure determination and analysis

For both VIR-7229 Fab:RBD and S2V29 Fab:RBD crystallography, additional, non-competing Fabs (S309 or S2H97) were added during Fab:RBD complexation to support crystal formation. XBB.1.5 RBD was deglycosylated with EndoH (25,000 U/mg RBD) and mixed with a 1.1-fold molar excess of VIR-7229^E^ Fab and S309^RK^ Fab. BQ.1.1 and EG.5 RBDs were deglycosylated with EndoH (25,000 U/mg RBD) and the C-terminal purification tag was cleaved with thrombin (20 U/mg RBD). Deglycosylated and tagless BQ.1.1 and EG.5 RBDs were mixed with a 1.1-fold molar excess of S2H97 and either S2V29 or VIR-7229 Fabs, respectively. The complexes were purified on a Superdex 200 10/300 GL column pre-equilibrated with 20 mM Tris-HCl pH 8.0, 50 mM NaCl for the BQ.1.1 complex or 25 mM Tris-HCl pH 7.5, 50 mM NaCl for the XBB.1.5 and EG.5 complexes. Crystals of all three complexes were obtained by the sitting-drop vapor diffusion method at 20°C.

For XBB.1.5 RBD-VIR-7229^E^- S309^RK^, a total of 200 nL complex at 8 mg/ml was mixed with 200 nL mother liquor solution from the Morpheus protein crystallization screen^80^ containing 0.1 M carboxylic acids (0.02 M sodium formate, 0.02 M ammonium acetate, 0.02 M sodium citrate tribasic dihydrate, 0.02 M potassium sodium tartrate tetrahydrate, 0.02 M sodium oxamate), 0.1 M buffer system 2 pH 7.5 (sodium HEPES, MOPS), and 30% precipitant mix 2 (20% v/v ethylene glycol, 10% w/v PEG 8000). Crystals were flash frozen in liquid nitrogen using the mother liquor solution as a cryoprotectant.

For EG.5 RBD-VIR-7229-S2H97, a total of 200 nL complex at 8 mg/ml was mixed with 200 nL mother liquor solution containing 0.1 M TRIS pH 8, 22% w/v PEG-MME 2000, and 20 mM NiCl_2_. Crystals were flash frozen in liquid nitrogen using the mother liquor solution supplemented with 20% ethylene glycol as a cryoprotectant.

For BQ.1.1-S2V29-S2H97, a total of 200 nL complex at 7.7 mg/mL was mixed with 200 nL mother liquor containing 0.1 M Tris pH 8.5, 20% PEG-MME 2000, and 10 mM NiCl_2_. Crystals were flash frozen in liquid nitrogen using the mother liquor solution supplemented with 20% ethylene glycol as a cryoprotectant.

For XBB.1.5 and EG.5 complexes, data were collected at beamline 14–1 at the Stanford Synchrotron Radiation Lightsource facility in Stanford, CA. Data were processed with the XDS software package^81^ for final datasets of 2.41 Å in space group P2_1_2_1_2_1_ and 1.90 Å in space group P2_1_2_1_2_1_ for XBB1.5 RBD-VIR-7229^E^-S309^RK^ and EG.5 RBD-VIR-7229-S2H97, respectively. For BQ.1.1-S2V29-S2H97, data were collected at beamline 8.2.1 at the Advanced Light Source in Berkeley, CA, and processed similarly to a final resolution of 1.67 Å in space P2_1_2_1_2_1_.

The complex structures were solved by molecular replacement using Phaser^82^ from starting models consisting of RBD-S309 (PDB: 7R6W) or RBD-S2H97 (PDB: 7M7W) and homology models for the respective Fabs generated using the Molecular Operating Environment (MOE) software package (Chemical Computing Group, https://www.chemcomp.com). Subsequent rounds of model building and refinement were performed using Coot,^83^ Refmac5,^84^ and Phenix.^85^ Validation was performed using Molprobity.^86^

The VIR-7229 epitope was defined as RBD residues within 5 Å of any VIR-7229 residue, determined from the unprotonated structures.

#### CryoEM sample preparation, data collection and data processing

For one dataset, BA.2.86 Hexapro S was incubated at 1 mg/ml with a 1.5 molar excess of VIR-7229 Fab during 30–45 seconds at room temperature. For the other two datasets, BA.2.86 Hexapro S precomplexed with a 1.5 molar excess of S309 Fab was incubated for 5–10 min with a 1.5 Fab molar excess of VIR-7229 Fab for 30–45 seconds. Three microlitres of the complexes were loaded onto freshly glow discharged R 2/2 UltrAuFoil (Electron Microscopy Sciences), M4-Au300–2.0/1.0 holey NiTi grids (Single Particle LLC) or C-Flat 2/2–4Cu-50 (Electron Microscopy Sciences) covered with a thin layer of manually added carbon before plunge-freezing using a vitrobot MarkIV (ThermoFisher Scientific) with a blot force of 0 and 6–6.5 s blot time or blot force of −1 and 4.5 s blot time for thin carbon grids, at 100% humidity and 21 °C. Data were acquired on a FEI Titan Krios transmission electron microscope operated at 300 kV and equipped with a Gatan K3 direct detector and Gatan Quantum GIF energy filter, operated in zero-loss mode with a slit width of 20 eV. Automated data collection was carried out using Leginon^87^ at a nominal magnification of 105,000× with a pixel size of 0.835 Å. The dose rate was adjusted to ~10 electrons per pixel per second, and each movie was fractionated in 100 frames of 40 ms per frame. A total of three datasets were collected to obtain the final structure with a defocus ranging between 0.8 and 2 μm, yielding a total of 29,989 micrographs. Data collected using sample vitrified on gold grids and NiTi grids comprised untilted data along with data collected with the stage tilted at 30° and 45° to circumvent particle preferential orientation^88^ whereas data collected from the sample vitrified on C-Flat grids covered with thin carbon comprised untilted data only. For each dataset, movie frame alignment, estimation of the microscope contrast-transfer function parameters, particle picking, and extraction were carried out using Warp.^89^ Particles were extracted with a box size of 400 pixels with a pixel size of 1.67 Å. Two rounds of reference-free 2D classification were performed in cryoSPARC^90^ to select well-defined particle images. After 2D classification, particles from three datasets were combined and an initial model was generated, using ab-initio reconstruction in cryoSPARC,^90^ and used as reference for heterogenous 3D refinement. Particles belonging to classes with the best resolved S density were selected. To improve particle picking further, the Topaz picker^91^ was trained on Warp-picked particle sets belonging to the selected classes after heterogeneous 3D refinement. The particles picked using Topaz were extracted and subjected to two rounds of 2D-classification followed by heterogenous 3D refinement in cryoSPARC. The two different particle sets picked from Warp and Topaz were merged and duplicate particle picks were removed in cryoSPARC using a minimum distance cutoff of 90 Å. After two rounds of heterogeneous refinements, the particles belonging to the class with the best resolved RBD:VIR-7229 Fab density were selected and used to carry out a non-uniform refinement (NUR).^92^ Particles from the NUR were transferred from cryoSPARC to Relion using the pyem program package (https://github.com/asarnow/pyem)^93^ and subjected to the Bayesian polishing procedure^94^ in Relion^95,96^ during which particles were re-extracted with a box size of 512 pixels and a pixel size of 1.0 Å. After polishing, particles were subjected to 2D-classification followed by a heterogeneous refinement in cryoSPARC to select particles belonging to the class with the best-resolved RBD:VIR-7229 Fab density. NUR with per-particle defocus refinement yielded a final reconstruction of BA.2.86 S in complex with VIR-7229 Fab at 3.1 Å resolution comprising 314,440 particles (the S309 density was largely averaged out). To further improve the density at the RBD:VIR-7229 Fab interface, local refinement was performed using cryoSPARC with a soft mask comprising the RBD and the VIR-7229 variable domains yielding a reconstruction at 3.3 Å resolution enabling model building. Reported resolutions are based on the gold-standard Fourier shell correlation (FSC) of 0.143 criterion.^97,98^ Local resolution estimation was carried out using cryoSPARC.

#### Model building and refinement

RBD and VIR-7229 Fab complex models were built and refined by iterating between manual rebuilding in Coot^99^ and refinement in Rosetta^100,101^. Validation was done using Phenix and Molprobity.^85,86^ Figures were generated using UCSF ChimeraX.^102^

#### Molecular dynamics analysis of Fab:RBD and ACE2:RBD

The coordinates of VIR-7229:RBD (EG.5) and VIR-7229:RBD (XBB.1.5) were obtained from the present work, and ACE2:RBD (XBB.1.5) from PDB ID 8FXB (ACE2:RBD [XBB.1])^103^ with F486P mutagenesis and rotamer optimization (to change XBB.1 to XBB.1.5) performed in silico in MOE using ProteinBuilder (MOE 2022.02; https://www.chemcomp.com). Glycan coordinates were taken from previous work.^32^ These models were prepared using QuickPrep (MOE 2022.02; https://www.chemcomp.com).

The three complexes were parameterized for molecular dynamics (MD) as previously described^79^ using AMBER^104^ with the ff14SB protein force field,^105^ GLYCAM_06j-1 glycan force field,^106^ TIP3P water force field,^107^ and Joung and Cheatham ions force field.^108^

For the VIR-7229:RBD (EG.5) and VIR-7229:RBD (XBB.1.5) complexes, the equilibration protocol followed that used in previous studies.^109,110^ By seeding with different initial velocities, five independent 0.8 μs AMBER MD simulations (4.0 μs) were executed for each of the two complexes.

For ACE2:RBD (XBB.1.5), five independent trajectories (each with production simulation time 0.9 μs) were generated for a total of 4.5 μs. Equilibration and production MD were run with OpenMM 8.^111^ Equilibration was performed according to a multistage protocol as previously described^110^ with the exception that the following atoms were left unrestrained in all stages of the equilibration protocol: the ACE, NME caps and missing loops in 8FXB. Energy minimization stages were performed using the OpenMM 8 LocalEnergyMinimizer with an energy tolerance of 10 kJ/mol. The molecular dynamics stages used the OpenMM 8 LangevinMiddleIntegrator.^112–114^ Hydrogen atom masses were set to 4 amu by transferring mass from connected heavy atoms, bonds to hydrogen were constrained, and center of mass motion was not removed. Pressure was controlled by a molecular-scaling Monte Carlo barostat with a pressure of 1 atmosphere, a temperature of 300 K, and an update interval of 50 steps. Non-bonded interactions were treated with the Particle Mesh Ewald method^115^ using a real-space cutoff of 1.0 nm and an Ewald error tolerance of 0.00025, with grid spacing selected automatically. Long range anisotropic dispersion corrections were applied to steric interactions.^116^ A virtual bond was added between the first atoms of each protein chain to ensure that the chains are imaged together. Default parameters were used unless noted otherwise. The code for running equilibration and molecular dynamics is available at: https://github.com/choderalab/rbd-ace2-xbb15-simulations.

After excluding the first 2.5 ns of MD, trajectories were post-processed followed by scripted protein:protein contact analysis in MOE (CCG MOE 2022.02) as previously described.^109^ A contact is defined for residue:residue (or residue:glycan) distances within 5 Å; inter-residue contacts were evaluated over all MD frames (sampled every 10 ns). Fraction occupancy for each RBD:mAb or RBD:ACE2 residue:residue (or residue:glycan) pair was calculated as the percentage of MD frames where a contact was observed. Contact analysis for the static structures were performed on the models after QuickPrep (MOE 2022.02; https://www.chemcomp.com).

#### Selection of SARS-CoV-2 mAb escape mutants by rVSV serial passaging

##### Propagation of replicating VSV-SARS-CoV-2 S chimeras (rVSV)

Wuhan-Hu-1 rVSV (GFP) was produced as described earlier.^117^ Omicron rVSV-spike constructs were designed in-house and purchased from VectorBuilder (en.vectorbuilder.com) and propagated in Vero-TMPRSS2 cells. For stock production, 100mm dishes (Falcon Cat. 353003) of Vero-TMPRSS2 were infected at MOI = 0.03 in infection medium (DMEM Gibco Cat. #11995–040, 1% Pen/strep Gibco Cat. #15140–122, 2% FCS VWR Cat. #97068–085, 20mM HEPES pH 7.7 Gibco Cat. 15630080). Plates were incubated at 34°C, 5% CO_2_ for 1 hour, after which inoculum was removed and fresh media was added (same formulation). Plates were incubated at 34°C, 5% CO_2_ for 72 hours. CPE was assessed visually, and virus containing media was removed, clarified and stored at −80°C for later use.

##### rVSV titration

Vero-TMPRSS2 cells were plated in a 12-well format (CellTreat Cat. 229111). A five-point curve of 10-fold rVSV-spike dilutions were prepared (starting at 1:100 dilution) in infection medium. Diluted virus was incubated with the cells for 1 hour, after which inoculum was removed and plates washed once with PBS (Gibco Cat. 10010023). An overlay of 1% methylcellulose (Sigma Aldrich Cat. M7027–250G) was added to each well, and plates were incubated at 34°C for 24 hours. Overlays were removed and plates fixed in 4% PFA for 30 minutes, and washed 3 times with PBS. Cells were permeabilized with 0.25% Triton X-100 (Sigma Aldrich Cat. X100–100ML) and stained with mouse anti-VSV-N primary antibody (clone 10G4, Kerafast Cat. EB0009). Cells were washed 3 times with PBS and stained with an anti-mouse secondary antibody conjugated to either horseradish peroxidase (HRP) or AlexaFluor647 at 1:1000 in 200 μL for 30 minutes at RT. For cells stained with HRP-conjugated secondary antibody, focus forming units (FFU) were visualized with TrueBlue reagent and foci were manually counted to calculate virus titers. For cells stained with the AlexaFluor647-conjugated secondary antibody, plates were imaged using a Cytation5 plate reader. Whole well images were acquired (12 images at 4X magnification per well) and the number of VSV N+ foci were manually counted to calculate virus titers.

##### rVSV Serial Passaging

To produce an even monolayer in a 12-well format, 2.5E5 Vero-TMPRSS2 cells were plated in 2mL of complete DMEM (10% FCS). After 24 hours, rVSV-spike aliquots were thawed and diluted to infect cells at MOI = 2 after neutralization. Antibody dilutions (7-point, 4-fold, final maximum concentration = 20 μg/mL) were prepared in 2 mL deep-well plates (Nunc Cat. 260251) and diluted rVSV-spike was added. Antibody and virus mixture was incubated at 37°C for 1 hour. After neutralization, virus was added to cells and incubated at 37°C for 1 hour, with gentle rocking every 15 minutes. Inoculum was removed, wells were washed once with PBS, and fresh media was added that contained matched concentrations of antibody. Plates were incubated at 37°C for 72 hours, and percent cytopathic effect (CPE) was assessed via inverted light microscope. Cell culture supernatants were harvested from cells treated with the highest concentration of antibody at which infected cells exhibited >20% CPE. Supernatants from the selected antibody or no-antibody well were collected, clarified and stored at −80°C for later use.

For subsequent passages, Vero-TMPRSS2 were plated as before. Passage 1 virus was diluted 1:5 in media containing antibody (diluted as before) and neutralized as above. Infection, selection, and collection were performed identically for each passage. Passaging was ceased after complete escape (CPE >20% at 20 μg/mL) or after 7–10 passages had elapsed. All passaging experiments were performed in duplicate.

##### rVSV Resistance Mutant Sequencing

Using Trizol (Invitrogen Cat. 15596026) phase extraction followed by column cleanup (Macherey-Nagel Cat. 740983.50), RNA was extracted from rVSV-spike samples. RNA was reverse transcribed using Protoscript II reverse transcriptase (NEB Cat. M0368L) and cDNA for the SARS-CoV-2 spike gene was amplified in two fragments using the following primers (IDT) and KAPA HiFi HotStart ReadyMix (Roche Cat. 07958935001): ATTGCCACTAGTCTCTAGTC & CAAGAACAACAGCCCTTGAG, CTTTACAAGGGAACGATTGAGC & ATCGGAAGAGAATTGAATTTCC. PCR products were cleaned up using NucleoSpin Gel and PCR Clean-up (Macherey-Nagel Cat. 740609.250) and purity visualized via gel electrophoresis (Invitrogen Cat. A42100). Samples were submitted to MCLab (www.mclab.com) for Sanger sequencing, or Primordium (primordiumlabs.com) for Nanopore sequencing. Results were analyzed with Snapgene software (www.snapgene.com).

#### Plaque-based selection of SARS-CoV-2 mAb escape mutants by rVSV

##### Production of VSV-SARS-CoV-2 S chimeras

Recovery of recombinant VSV was performed as described.^118^ Briefly, BSRT7/5 cells^119^ were inoculated with vaccinia virus vTF7–3^120^ and subsequently transfected with T7-expression plasmids encoding VSV N, P, L, and G, and an antigenomic copy of the viral genome. Cell culture supernatants were collected at 72 h, clarified by centrifugation (5 min at 1,000 × g), and filtered through a 0.22 mm filter. Virus was plaque-purified on Vero CCL81 cells in the presence of 25 mg/mL of cytosine arabinoside (Sigma-Aldrich), and plaques in agarose plugs were amplified on Vero CCL81 cells. Viral stocks were amplified on MA104 cells at an MOI of 0.01 in Medium 199 containing 2% FBS and 20 mM HEPES pH 7.7 at 34 °C. Viral supernatants were harvested upon extensive cytopathic effect and clarified of cell debris by centrifugation at 1,000 × g for 5 min. Aliquots were maintained at −80 °C.

##### Selection of monoclonal antibody resistant mutants (MARMs)

Replicating VSV-SARS-CoV-2 S chimeras were used to select for MARMs as previously described.^117,121^ In brief, MARMs were recovered by plaque isolation on Vero E6 cells (ATCC, CRL-1586) with the indicated monoclonal antibody in the overlay. The concentration of monoclonal antibody in the overlay was determined by neutralization assays at a MOI of 100. Escape clones were plaque-purified on Vero cells (ATCC, CCL-81) in the presence of monoclonal antibody, and plaques in agarose plugs were amplified on MA104 cells (a gift from H. B. Greenberg (Stanford School of Medicine)) with the monoclonal antibody present in the medium. Viral stocks were amplified on MA104 cells at an MOI of 0.01 in Medium 199 containing 2% FBS and 20 mM HEPES pH 7.7 (Millipore Sigma) at 34 °C. Viral supernatants were collected upon extensive cytopathic effect and clarified of cell debris by centrifugation at 1,000g for 5 min. Aliquots were maintained at −80 °C. Viral RNA was extracted from VSV-SARS-CoV-2 mutant viruses using RNeasy Mini kit (Qiagen), and S was amplified using OneStep RT–PCR Kit (Qiagen). The mutations were identified by Sanger sequencing (Genewiz). Their resistance was verified by subsequent virus infection in the presence or absence of antibody. In brief, Vero cells were seeded into 12-well plates for overnight. The virus was serially diluted using DMEM and cells were infected at 37 °C for 1 h. Cells were cultured with an agarose overlay in the presence or absence of monoclonal antibody at 34 °C for 2 days. Plates were scanned on a biomolecular imager and expression of eGFP is shown at 48 h after infection.

#### Cell-cell fusion between VeroE6-TMPRSS2 and SARS-CoV-2 expressing cells

##### Split GFP cell-cell fusion assay

To evaluate the effect of point mutations in the XBB.1.5 and EG.5 S backgrounds in fusion, we used the split GFP cell-cell fusion assay. Briefly, BHK21 cells transfected with the different S point mutants and VeroE6-TMPRSS2 stably expressing GFP1–10 and GFP 11, respectively, were utilized to quantify cell-cell fusion over 18 hours. BHK21-GFP1–10 cells were cultured in 6-well cell-culture grade plates and incubated overnight in DMEM containing 10% FBS and 8 μg/ml puromycin. Next day, BHK21-GFP1–10 cells were transfected with 4ug of DNA encoding one S construct, Opti-MEM and lipofectamine2000 (Invitrogen Cat# 11668027) following manufacture instructions and VeroE6-TMPRSS2 GFP11 cells in DMEM containing 10% FBS, 8 μg/ml puromycin and 4 μg/ml blasticidin were seeded in a black, glass bottom 96-well plate at ~36,000 cells per well and incubated overnight. After ~20 hr incubation, transfected BHK21-GFP1–10 cells were washed three times with FluoroBrite DMEM (Gibco Cat# A1896701) and incubated with 500uL of Gibco cell dissociation buffer for 10 minutes at 37 °C with agitation to facilitate resuspension of cells. Cells were then passed through cell-strainer capped tubes (Falcon Cat# 08–771-23) to eliminate aggregates and diluted using FluoroBrite DMEM to have ~60,000 cells per mL. VeroE6-TMPRSS2-GFP11 cells were carefully washed with FluoroBrite DMEM three times and 150 uL of transfected BHK21-GFP1–10 cells were added on top and co-cultured for a period of ~18 hours in the Cytation7 plate imager. Data were analyzed with GraphPad PRISM 10 to quantify GFP+ areas.

##### Flow cytometry for normalization of expression

Flow cytometry was utilized to quantify S expression levels on the cell surface of BHK21-GFP1–10 cells. BHK21-GFP1–10 cells under puromycin selection were cultured in 6 well cell-culture grade plates and incubated overnight. After ~20hrs, the BHK21-GFP1–10 cells were washed with unenriched DMEM and 2.5 mL of fresh DMEM with 10% FBS was added to cells prior to transfection step. To match conditions with the cell-cell fusion assay, cells were transfected with 4ug of S plasmid and lipofectamine2000 in Opti-MEM and incubated for ~38 hours. The BHK21-GFP1–10 cells were then washed with Flow Stain buffer and 500 uL of Gibco cell dissociation buffer was added to remove cells from bottom of the 6 well plates. Cells were resuspended with Flow Stain buffer (eBioscience Cat# 00–4222-26), filtered through a cell-strainer capped tube, diluted to ~1×10^7 cells per mL. and added to a pyramid bottom 96-well plate. Cells in the plate were centrifuged and the supernatant discarded. Cells were resuspended in Flow Stain Buffer containing 5 μg/mL of S2L20 mAb^122^ and incubated at 4 °C for ~25 minutes. Cells were washed 3 times with 100 μl of flow stain buffer before adding 0.5 μg of Anti-IgG Fc-PE Secondary Antibody per well (Thermo Fisher Scientific Cat#12–4998-82). After ~25 minutes at 4 °C, cells were washed 3X with Flow Stain Buffer. To fix the cells, 100uL of 2% paraformaldehyde was added to each well and incubated at 4 °C for 15 minutes. Cells were then washed 2 times with Flow Stain Buffer prior to being resuspended in 50 μL of fresh Flow Stain Buffer. Cells were transferred into tubes with 450 μL of Flow Stain Buffer, kept on ice, shielded from direct light prior to being counted on BD Biosciences’ FACSymphony. All results were analyzed on BD FlowJo 10.8.2. For normalization of expression and cell-cell fusion, total percentage of fusion after 18 hours was divided by mean fluorescence intensity (MFI).

#### Prevalence analysis in GISAID database

The viral sequences and the corresponding metadata were obtained from GISAID EpiCoV project (https://www.gisaid.org/). Analysis was performed on sequences submitted to GISAID up to May 8, 2024, unless otherwise specified. S protein sequences were either obtained from GISAID download page or, for the most recently submitted sequences, from the genomic sequences with exonerate 2.4.0–haf93ef1_3 (https://quay.io/repository/biocontainers/exonerate?tab=tags ) using protein to DNA alignment with parameters -m protein2dna –refine full –minintron 999999 –percent 20 and using accession NC_045512.2 as a reference. Multiple sequence alignment of all spike proteins was performed with mafft 7.508—hec16e2b_0 (https://quay.io/repository/biocontainers/mafft?tab=tags&tag=7.508--hec16e2b_0) with parameters --mapout --auto --op 4.5 --reorder --keeplength --addfragments using the same reference as above. The --mapout parameter was used to retrieve insertions. S sequences that were <80% (1019/1273) of the canonical protein length were discarded. To identify each mutation prevalence, missingness (or ambiguous amino acids) was taken into account in both nominator and denominator. Per week prevalence of each mutation was then calculated to get the temporal trend.

#### Bioinformatic analysis of intra-individual SARS-CoV-2 genomic variability

We used low frequency viral variants occurring intra-individual as a proxy to estimate the replication error rate of the SARS-CoV-2 RNA polymerase, i.e. how frequently is a variant sampled in the absence of pressure in the SARS-CoV-2 genome. This strategy has the caveat that it could also capture polymerase errors introduced during the library preparation and sequencing errors. The former should however be orders of magnitude less common than the viral polymerase error rate, and therefore should introduce limited noise, while the latter can be minimized with stringent quality controls filtering. We analyzed raw data (fastq) rather than consensus sequences in order to identify intra-individual variation. 1,763 SARS-CoV-2 samples (paired fastq files) collected from June 2022 to Dec 2023 were downloaded from the SRA database using fastq-dump of sratoolkit (version 3.0.7) [parameters: *–split-files*]. Only samples sequenced with Illumina were selected for this analysis, in order to limit the technological error rate. Variants were called against the consensus of the respective sample using an in-house pipeline that leverages trimmomatic v.0.39,^123^ bwa-mem v.0.7.17,^124^ lofreq v.2.1.5,^125^ and bcftools v.1.10.2.^126^ As mutation rates have been shown to be context dependent, i.e. are influenced by the nucleotide(s) adjacent to the variant,^127^ we computed trimer variation rates. The frequency of each trimer variant (N=192, or 4^3*3, i.e each possible trimer varying to 3 alternate trimers based on the middle nucleotide) was calculated for each sample, as follows: The numerator is the number of times a trimer variant (e.g. TTG > TGG) event is seen in a sample, and the denominator is the number of times the respective trimer (e.g. TTG) is seen in the viral genome consensus sequence of the corresponding sample. The minimum number of read coverage is set at 50. In order to minimize the potential technological error rate interference, a variant event was considered for each mutation with allelic frequency >1% that passed all of the following criteria: (i) at least 2 reads carrying the mutation, (ii) a Phred quality score of ≥27 (corresponding to an error rate ~0.02%) at the mutation position, (iii) an average read quality score of ≥27 for the reads carrying the mutation, (iv) a read quality score of ≥30 for ≥50% of the reads carrying the mutation and (v) no positional bias in the read (e.g. the mutation always being located at the same position in the read). The frequency obtained for each trimer variant is then aggregated across individuals (extracting the mean and standard error). The threshold of 1% was empirically chosen to minimize the possibility of sequencing errors (the higher the threshold, the less likely we would capture technical errors), while also minimizing the capture of variants under pressure (the higher the threshold, the more likely the variant would be under pressure and detectable at higher proportion in the sample).

### QUANTIFICATION AND STATISTICAL ANALYSIS

Description of the analysis of neutralization assays can be found in Method Details sections, “Neutralization of sarbecovirus pseudoviruses” and “Neutralization of authentic SARS-CoV-2 viruses.” Final IC50 values are the geometric mean of per-replicate measurements, with exact values of *n* given in Data S1.

Description of the analysis of SPR binding assays can be found in Method Details section “Affinity determination by surface plasmon resonance (SPR),” analyzed via Biacore Insight software. Final K_D_ values were computed as the mean and standard deviation across replicates, with exact values of *n* given in Data S1.

Description of the analysis of yeast-display pan-sarbecovirus binding assays can be found in Method Details section “Evaluation of sarbecovirus cross-reactivity via high-throughput yeast-display binding assays.” The final EC50 reported is the robust mean (eliminating top and bottom 5% of values) across internally replicated barcodes linked to each RBD library variant. Full quantitative analysis pipeline is available from GitHub: https://github.com/tstarrlab/SARSr-CoV_mAb-breadth_S2V29.

Statistical analysis of *in vivo* hamster protection studies is described in the Figure 2 legend, with exact *n* defined in Method Detail section, “In vivo efficacy evaluation using a Syrian hamster model.” Significance was established via comparison of median values via ANOVA non-parametric Kruskal-Wallis test followed by Dunn’s multiple comparison test.

Description of the analysis of yeast-display deep mutational scanning escape mapping can be found in Method Details section “Evaluation of escape mutants via yeast-display deep mutational scanning. The final escape fraction is the mean of experimental duplicates. Full quantitative analysis pipeline is available from GitHub: https://github.com/tstarrlab/SARS-CoV-2-RBD_Omicron_MAP_S2V29.

## Supplementary Material

1 Data S1. Binding, neutralization, and sequences related to Figures 1, 5, 6, S1, and S6

2 Data S2. Polyreactivity and tissue cross-reactivity related to Figure 1

3 Data S3. In vivo efficacy related to Figure 2

4 Data S4. Molecular dynamics simulation data related to Figure 5

5 Data S5. Resistance selection, bioinformatic analysis, and pseudovirus infectivity related to Figures 6 and S6

6 **Figure S1. Potency and breadth of comparator mAbs. Related to** Figure 1.
(A) Sequence variation among SARS-CoV-2 sequences in GISAID (blue, left) or sarbecoviruses (orange, right) mapped to the SARS-CoV-2 Wuhan-Hu-1 RBD structure (PDB: 6M0J).
(B) Pseudovirus neutralization plotted versus mAb concentration for VIR-7229 and parent mAb S2V29, for Wuhan-Hu-1 and SARS-CoV-1, utilizing VeroE6 cells.
(C-I) Neutralization of SARS-CoV-2 variant or sarbecovirus pseudoviruses mediated by S2V29 parent mAb and six comparator mAbs. Bar colors and horizontal lines as in Figure 1A; orange horizontal line for S309 indicates Vero-TMPRSS2 cells. S309, parent mAb of sotrovimab,^15^ and SA55^42^ were isolated from SARS-CoV-1 survivors, the latter after receipt of a vaccine based on SARS-CoV-2 Wuhan; S2K146^33^ and S2X259^21^ were isolated from SARS-CoV-2 patients after a pre-Omicron infection; Omi-42,^27^ like S2V29, was isolated from vaccinees following a breakthrough Omicron infection; VYD222 was affinity-matured from the ADI-55688 mAb.^16,17,19^ Asterisk indicates maximum neutralization plateaus at ~90% due to entry mediated by VSV-G (i.e. pseudovirus preparations with reduced titer). Orange data points are previously published: S2X259^21^ and sotrovimab.^13^ SARS-CoV-2 strains which completely escape Omi-42 all share the F456L mutation. See also Data S1.
(J) VYD222 Fab fragment binding affinity to recombinant RBDs measured by SPR. Bar color denotes sarbecovirus clade, as in Figure 1C. SARS-CoV-1 RBD is the Urbani strain. See also Data S1.

7 **Figure S2. VIR-7229 mechanisms of action. Related to** Figures 1 and 2.
(A) VIR-7229 Fab fragment competes with monomeric ACE2 for binding to Wuhan-Hu-1 RBD, as measured by bio-layer interferometry (BLI). All comparator mAbs also compete with ACE2, with the exception of S309.
(B) VIR-7229 efficiently promotes S_1_ shedding from Wuhan-Hu-1 and XBB.1.5 SARS-CoV-2 S transiently expressed on the surface of Expi-CHO cells, similar to positive control mAb S2K146, whereas anti-NTD negative control mAb S2M28 does not.
(C-D) Activation of human FcγRIIa (C) and FcγRIIIa (D) was evaluated using a bioreporter assay. Target cells were CHO stably expressing SARS-CoV-2 Wuhan-Hu-1 S and effector cells were Jurkat expressing the indicated FcγR and engineered with a NFAT-mediated luciferase reporter to reflect activation of human FcγRs. Data points show means ± SD of duplicates.
(E) Antibody-dependent cell cytotoxicity (ADCC; NK-cell mediated) was evaluated using freshly isolated cells from two previously genotyped donors (FcγRIIIa): heterozygous (F/V158; left) or homozygous high-affinity (V/V158; right). Target cells had surface expression of SARS-CoV-2 Wuhan-Hu-1 S and intracellular expression of HiBiT; ADCC was measured using NanoLuc HiBiT Extracellular Detection Reagent.

8 **Figure S3. Cryo-EM data processing and validation of VIR-7229-bound BA.2.86 S. Related to** Figure 3.
(A, B) Representative electron micrographs (A) and class averages (B) of BA.2.86 S in complex with VIR-7229 Fab. Scale bars, 100 nm (A).
(C) Gold-standard Fourier shell correlation curves for the S trimer bound to two VIR-7229 Fabs (black line) and the locally refined reconstruction of an RBD and VIR-7229 variable domains (grey line).
(D, E) Local resolution map for the S trimer bound to two VIR-7229 Fabs (D) and the locally refined reconstruction of an RBD and VIR-7229 variable domains (E).
(F) Cryo-EM data processing flowchart.

9 **Figure S4. Structural analysis of VIR-7229:RBD and ACE2:RBD. Related to** Figures 3 and 5.
(A) Superposition of VIR-7229-bound and human-ACE2-bound (PDB: 6M0J) SARS-CoV-2 RBD structures; steric clash between VIR-7229 and ACE2 is indicated with a red asterisk.
(B) Ribbon diagram of VIR-7229 Fab-bound XBB.1.5 RBD indicating a conformational change of residues 473 to 489 relative to apo XBB.1.5 RBD (PDB:8JYK) and ACE2-bound XBB.1 RBD (PDB: 8FXB; S309 Fab also bound).
(C) Summary of MD simulations of XBB.1.5 RBD or EG.5 RBD bound to VIR-7229 Fab (dynamic) as compared to analysis of X-ray structures (static). Boxes indicate the sum of fraction occupancies of VIR-7229 contacts to each RBD residue, as in Figure 5D. Contacts in the X-ray structure are treated as 100% occupancy. Contacts in the MD simulation beyond the static epitope are shown if the sum of fraction occupancies is ≥0.1. Slash indicates no contact formed. See also Data S4.
(D) To illustrate the similarity of VIR-7229 and Omi-42 epitopes, VIR-7229 epitope is shown in orange on XBB.1.5 RBD structure and Omi-42 binding footprint is depicted as a blue outline.
(E) In silico modeling of L455W in a fully closed SARS-CoV-2 S structure (PDB 7K43; with S2M11 Fab bound) indicating expected clashes with a neighboring protomer. All energetically favored rotamers are sterically incompatible with the closed structure due to clashes with either the same or the neighboring protomer; one of the three most prevalent rotameric configurations was selected for visualization purposes. The two SARS-CoV-2 S protomers are shown in cyan and pink with the modeled L455W side chain highlighted as a red semi-transparent surface and steric clash indicated with a red asterisk.
(F) Summary of MD simulation of XBB.1.5 RBD bound to ACE2 (dynamic) as compared to analysis of X-ray structure (static). Boxes indicate the sum of fraction occupancies of ACE2 contacts to each RBD residue; full glycans were modeled on the RBD:ACE2 structure, MD contacts may be glycan mediated, as indicated in the third row (darker gray indicates a larger percentage glycan-mediated). Contacts in the MD simulation beyond the static epitope are shown if the sum of fraction occupancies is ≥0.1. Slash indicates no contact formed. See also Data S4.
(G) Zoomed-in view of the interface between two closed RBDs within a S trimer emphasizing the inter-protomer hydrogen bond formed between SARS-CoV-2 D420 and Y369 (PDB 6ZGE), clade 1a SARS-CoV-1 D407 and Y356 (PDB 7ZH1), or clade 3 PRD-0038 D411 and Y359 (PDB 8U29).

10 **Figure S5. Deep mutational scanning profiling of VIR-7229, S2V29, and S2K146 escape mutations. Related to** Figure 5.
(A) Representative FACS gates used to identify mutations that escape antibody binding. An antibody-escape gate was drawn that captures approximately 50% of the cells in the respective wildtype control labeled at 0.1x the library selection antibody concentration. The “escape fraction” represents the fraction of cells of a mutant genotype that fall into this antibody-escape FACS gate.
(B) For each experiment with VIR-7229, the correlation in the per-mutation escape fraction between duplicate library selections.
(C) Full deep mutational scanning escape profiles of parental mAb S2V29 (left) and VIR-7229 (right), compared to Figure 5A that illustrates VIR-7229 epitope profiles. For each experiment, lineplots (left) show the total escape at each site in the RBD, with sites of strong escape annotated with pink indicators. Logoplots (right) illustrate mutation-level escape fraction at sites of strong escape, with mutations colored according to mutational impact on ACE2-binding affinity. Note, mutations to T/S at sites 407 and 419 introduce N-linked glycosylation motifs due to the presence of N405 and N417 in the Omicron (but not Wuhan-Hu-1) variants.
(D) Deep mutational scanning escape profiling of the comparator mAb S2K146 (details as in (C)) illustrating a broadening of the functional epitope over evolutionary time, likely due to erosion of S2K146 affinity across variant evolution.

11 **Figure S6. Validation and fitness of VIR-7229 escape mutations. Related to** Figures 5 and 6.
(A) VIR-7229-mediated neutralization of SARS-CoV-2 pseudoviruses carrying mutations that were observed as DMS binding escapes in at least one strain background, plus F456L. Mutations were tested in different strain backgrounds, as indicated by bar color. Mutations are annotated by: (a) total counts in the GISAID database as of May 8, 2024, (b) fold-change reduction in ACE2 affinity in the XBB.1.5 background evaluated by DMS^60^ (available from https://tstarrlab.github.io/SARS-CoV-2-RBD_DMS_Omicron-XBB-BQ/RBD-heatmaps/), (c) impact on pseudovirus infectivity (assessed by evaluating the viral titer in comparison to the titer of respective unmutated backbone quantified in parallel; - indicates titers within 5-fold, ↓ indicates a titer more than 5-fold reduced in at least one backbone, ↓↓ indicates a titer more than 5-fold reduced in at least two backbones; see also Data S5), (d) minimum number of nucleotide changes required for the mutation to occur, and (e) the RBD background where the mutation was observed as a binding escape in the DMS experiment. Asterisk indicates that the reduced infectivity observed for A475N is only observed in the Wuhan-Hu-1 background. ND: Not determined; na: not applicable. See also Data S1.
(B) VIR-7229-mediated neutralization of SARS-CoV-2 pseudoviruses carrying mutations that were observed during the EG.5 rVSV resistance selection experiment. Strain background is indicated by bar color. For L455W, JN.1 is also the BA.2.86 background (JN.1 = BA.2.86+L455S). Mutations are annotated as in panel A, as well as by counts in the +F456L background in the GISAID database as of May 8, 2024. See also Data S1.
(C) VIR-7229-mediated neutralization of SARS-CoV-2 pseudoviruses carrying a set of epitope mutations below 0.005% frequency in GISAID but accessible by a single nucleotide change from wild-type sequences. Mutations were tested in either the BQ.1.1 or XBB.1.5 background; some mutations were tested in both backgrounds. See also Data S1.

12 Document S1. Figures S1–S6 and Tables S1–S3

## Figures and Tables

**Key resources table:** 

REAGENT or RESOURCE	SOURCE	IDENTIFIER
Antibodies		
FITC-conjugated chicken anti-Myc	Immunology Consultants Laboratory, Inc.	Cat# CMYC-45F
PE-conjugated goat anti-human IgG	Jackson ImmunoResearch	109–115–098
Goat anti-mouse StarBright Blue 700	Bio-Rad Laboratories	12004159
Mouse anti-V5 tag	Bio-Rad Laboratories	MCA1360GA
Anti-AviTag polyclonal antibody	GenScript	A00674–40
S2V29 (S2V29-v1.2) IgG1m17-LS	This paper	N/A
VIR-7229 IgG1m17-LS	This paper	N/A
S2X259 IgG1m3-LS	Tortorici, et al. 2021^21^	N/A
SA55 IgG1m17-LS	Cao, et al. 2022^42^	N/A
VYD222 IgG1m3-LA	Walker, et al. 2024^16^	N/A
S309 IgG1m17-LS	Pinto, et al. 2020^15^	N/A
S2K146 IgG1m17-LS	Park, et al. 2022^33^	N/A
Omi-42 IgG1m17-YTE	Nutalai, et al. 2022^27^	N/A
VIR-7229 with Syrian hamster IgG2 Fc	This paper	N/A
Bacterial and virus strains		
SARS-CoV-2 (Wild-type); hCoV-19/USA-WA1/2020	BEI Resources	NR-52281
SARS-CoV-2 Delta (B.1.617.2); hCoV-19/USA/PHC658/2021	BEI Resources	NR-55611
SARS-CoV-2 BA.1; hCoV-19/USA/MDHP20874/2021	BEI Resources	NR-56461
SARS-CoV-2 BA.2.3; hCoV-19/USA/MDHP24556/2022	BEI Resources	NRS-56511
SARS-CoV-2 BA.5; hCoV-19/USA/COR-22–063113/2022	BEI Resources	NRS-58616
SARS-CoV-2 XBB.1.5; hCoV-19/USA/MD-HP40900/2022	BEI Resources	NR-59104
SARS-CoV-2 XBB.1.16; hCoV-19/USA/CA-Stanford-139_S23/2023	BEI Resources	NR-59442
SARS-CoV-2 EG.5.1; hCoV-19/USA/MD-HP47946/2023	BEI Resources	NR-59576
SARS-CoV-2 FL.1.5.1; hCoV-19/USA/CA-AK001/2023	BEI Resources	NR-59686
SARS-CoV-2 JN.1; hCoV-19/USA/NY/PV96109/2023	BEI Resources	NR-59694
rVSV-spike-Wuhan-Hu-1; 21 amino acids deleted from spike C-terminus	Case, et al. 2020^72^	NCBI YP_009724390.1
rVSV-spike-XBB.1.5; C-terminal 19 amino acids deleted	VectorBuilder	N/A
rVSV-spike-EG.5; C-terminal 19 amino acids deleted	VectorBuilder	N/A
rVSV-spike-XBB.1.5.70; C-terminal 19 amino acids deleted	VectorBuilder	N/A
Biological samples		
ExpiFectamine 293 Transfection Kit	Gibco	A14526
ExpiFectamine CHO Transfection Kit	ThermoFisher Scientific	15627878
BirA biotin-protein ligase bulk reaction kit	Avidity	N/A
Chemicals, peptides, and recombinant proteins		
Kifunensine	Cayman Chemical	NC9744221
Endo H	New England Biolabs	P0702L
PE-streptavidin	Jackson ImmunoResearch	016–110–084
Critical commercial assays		
ADCC Reporter Bioassay	Promega	G7018 (V158)
FcγRIIa-H ADCP Bioassay	Promega	G9995 (FcγRIIa)
Deposited data		
XBB.1.5 RBD - VIR 7229 - S309	This paper	PDB ID 9AU1
BQ.1.1 RBD - S2V29 - S2H97	This paper	PDB ID 8S6M
EG.5 RBD - VIR 7229 - S2H97	This paper	PDB ID 9ATM
BA.2.86 S:VIR-7229 (Local refinement)	This paper	PDB ID 9ASD, EMD-43813
BA.2.86 S:VIR-7229 (Global refinement)	This paper	PDB ID 9AU2, EM-43842
Illumina sequencing: barcode counts for pan-sarbecovirus yeast-display binding assay	This paper	NCBI SRA, BioProject PRJNA714677, BioSample SAMN41715061
Illumina sequencing: barcode counts for DMS mAb-escape yeast-display assay	This paper	NCBI SRA, BioProject PRJNA770094, BioSample SAMN41694243
Experimental models: Cell lines		
HEK293T cells	ATCC	Cat# CRL-11268
Expi293F cells	ThermoFisher Scientific	A14527
ExpiCHO cells	ThermoFisher Scientific	A29127
Lenti-X 293T cells	Takara	632180
Vero E6 cells	ATCC	CRL-1586
Vero-TMPRSS2 cells	Lempp, et al. 2021 ^61^	N/A
Experimental models: Organisms/strains		
*Saccharomyces cerevisiae* strain AWY101	Wentz and Shusta 2007^75^	N/A
*Saccharomyces cerevisiae* strain EBY100	ATCC	MYA-4941
Male golden Syrian hamsters	Janvier Laboratories	Mesocricetus auratus; RjHan:AURA
Recombinant DNA		
Yeast-display deep mutational scanning plasmid library, Wuhan-Hu-1	Starr et al. 2022^79^	Addgene Cat# 1000000182
Yeast-display deep mutational scanning plasmid library, BA.2	Starr et al. 2022^53^	Addgene Cat# 1000000188
Yeast-display deep mutational scanning plasmid library, BQ.1.1	Taylor and Starr 2023^60^	Addgene Cat# 1000000231
Yeast-display deep mutational scanning plasmid library, XBB.1.5	Taylor and Starr 2023^60^	Addgene Cat# 1000000232
Yeast-display deep mutational scanning plasmid library, EG.5	This paper	Addgene Cat# pending
Yeast-display deep mutational scanning plasmid library, BA.2.86	This paper	Addgene Cat# pending
Plasmids encoding amino acid positions 328–531 of SARS-CoV-2 Wuhan spike (NCBI reference YP_009724390.1) and Omicron variant spike proteins; N-terminal signal peptide; C-terminal 8xHis-AviTag or thrombin-8xHis-AviTag	This paper	N/A
Plasmid encoding SARS-CoV-2 BA.2.86 (EPI_ISL_18097315, amino acids 1–1204) Hexapro spike ectodomain; C-terminal foldon-AviTag-8xHis	This paper	N/A
Plasmid encoding residues 19–615 of human ACE2 (Uniprot Q9BYF1); N-terminal signal peptide; C-terminal thrombin cleavage site-TwinStrep-10xHis-GGG-tag	This paper	N/A
		
Software and algorithms		
Custom computational pipeline for yeast-display pan-sarbecovirus binding assay	This paper	https://github.com/tstarrlab/SARSr-CoV_mAb-breadth_S2V29/blob/main/results/summary/summary.md
Custom computational pipeline for yeast-display mAb-escape DMS assay	This paper	
Biacore Insight Software	Cytiva	29310602
Cryosparc v4.4.0	Punjani et al., 2017^90^	https://cryosparc.com
Relion v3.0	Zivanov et al., 2018^95^	https://www3.mrc-lmb.cam.ac.uk/relion
Coot	Emsley et al., 2010^83^	https://www2.mrc-lmb.cam.ac.uk/personal/pemsley/coot/
Phenix	Liebschner et al., 2019^85^	https://phenix-online.org/download/
XDS	Kabsch, 2010^81^	https://xds.mr.mpg.de/
ChimeraX	Pettersen et al., 2004^102^	https://www.cgl.ucsf.edu/chimerax/
Prism 10	GraphPad Software	https://www.graphpad.com/features
Scikit-learn	Pedregosa et al., 2011^70^	scikit-learn: machinelearning in Python—scikit-learn 1.5.0documentation
Python version 3.10	Python software foundation	www.python.org

## Highlights

- Potent anti-SARS-CoV-2 mAb VIR-7229 is derived from human memory B cells
- VIR-7229 uniquely competes with ACE2 and has pan-sarbecovirus breadth
- VIR-7229 is resilient to epitope variation and has a high barrier to viral escape
- VIR-7229 is a strong candidate to become a next-generation medicine for COVID-19
