# Supplementary figures and images for: A potent pan-sarbecovirus neutralizing antibody resilient to epitope diversification
Source: Cell. Author manuscript; Available in PMC 2024 Dec 14. (PMC11645210; doi:10.1016/j.cell.2024.09.026)

A

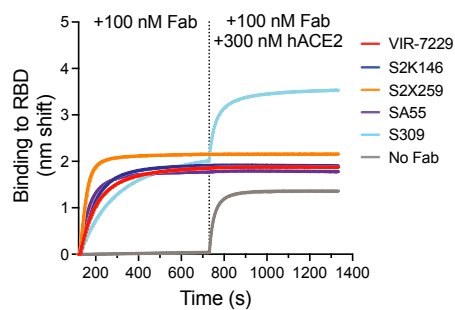

B

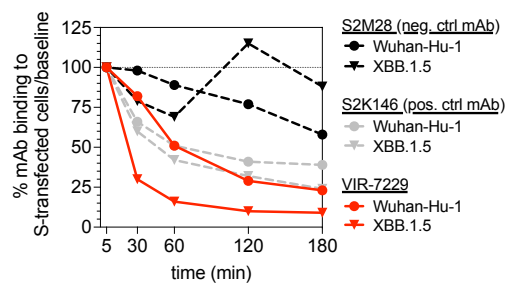

C

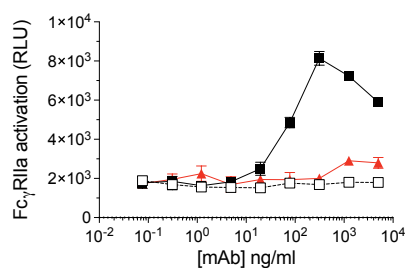

D

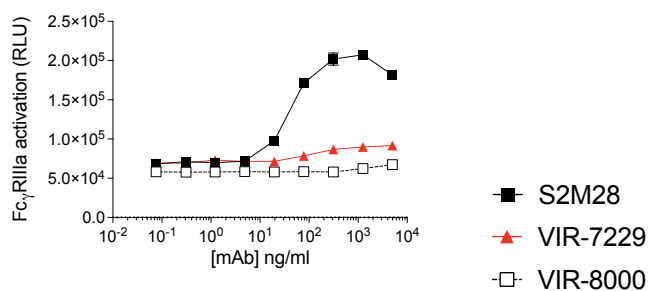

E

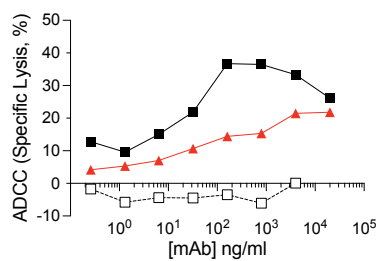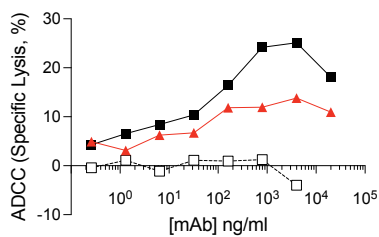

Supplement: 7 — Figure S2. VIR-7229 mechanisms of action. Related to Figures 1 and 2. (A) VIR-7229 Fab fragment competes with monomeric ACE2 for binding to Wuhan-Hu-1 RBD, as measured by bio-layer interferometry (BLI). All comparator mAbs also compete with ACE2, with the exception of S309. (B) VIR-7229 efficiently promotes S1 shedding from Wuhan-Hu-1 and XBB.1.5 SARS-CoV-2 S transiently expressed on the surface of Expi-CHO cells, similar to positive control mAb S2K146, whereas anti-NTD negative control mAb S2M28 does not. (C-D) Activation of human FcγRIIa (C) and FcγRIIIa (D) was evaluated using a bioreporter assay. Target cells were CHO stably expressing SARS-CoV-2 Wuhan-Hu-1 S and effector cells were Jurkat expressing the indicated FcγR and engineered with a NFAT-mediated luciferase reporter to reflect activation of human FcγRs. Data points show means ± SD of duplicates. (E) Antibody-dependent cell cytotoxicity (ADCC; NK-cell mediated) was evaluated using freshly isolated cells from two previously genotyped donors (FcγRIIIa): heterozygous (F/V158; left) or homozygous high-affinity (V/V158; right). Target cells had surface expression of SARS-CoV-2 Wuhan-Hu-1 S and intracellular expression of HiBiT; ADCC was measured using NanoLuc HiBiT Extracellular Detection Reagent. [file NIHMS2027680-supplement-7.pdf]

A

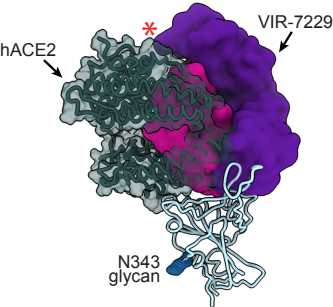

B

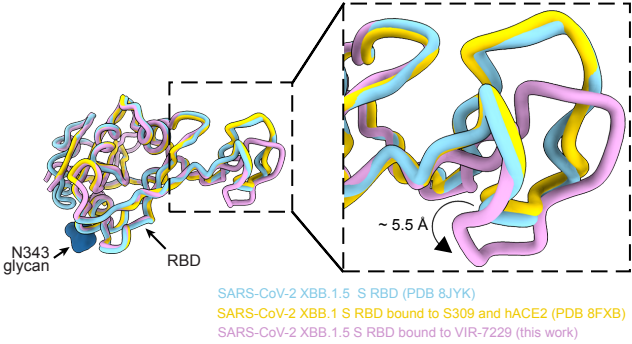

C

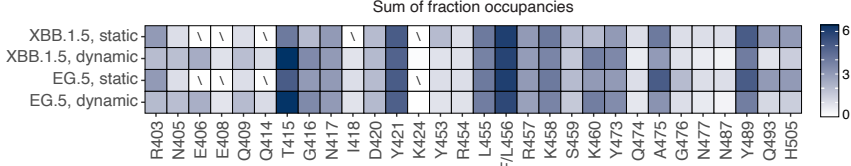

D

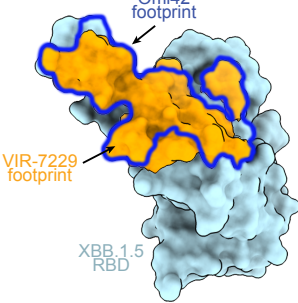

E

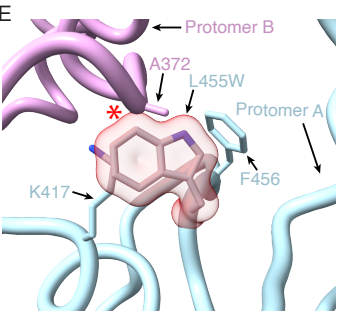

F

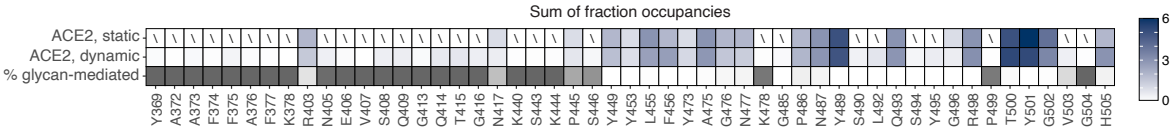

G

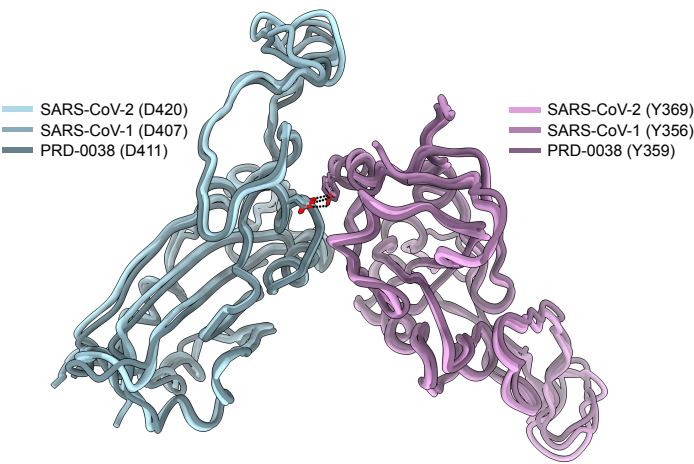

Supplement: 9 — Figure S4. Structural analysis of VIR-7229:RBD and ACE2:RBD. Related to Figures 3 and 5. (A) Superposition of VIR-7229-bound and human-ACE2-bound (PDB: 6M0J) SARS-CoV-2 RBD structures; steric clash between VIR-7229 and ACE2 is indicated with a red asterisk. (B) Ribbon diagram of VIR-7229 Fab-bound XBB.1.5 RBD indicating a conformational change of residues 473 to 489 relative to apo XBB.1.5 RBD (PDB:8JYK) and ACE2-bound XBB.1 RBD (PDB: 8FXB; S309 Fab also bound). (C) Summary of MD simulations of XBB.1.5 RBD or EG.5 RBD bound to VIR-7229 Fab (dynamic) as compared to analysis of X-ray structures (static). Boxes indicate the sum of fraction occupancies of VIR-7229 contacts to each RBD residue, as in Figure 5D. Contacts in the X-ray structure are treated as 100% occupancy. Contacts in the MD simulation beyond the static epitope are shown if the sum of fraction occupancies is ≥0.1. Slash indicates no contact formed. See also Data S4. (D) To illustrate the similarity of VIR-7229 and Omi-42 epitopes, VIR-7229 epitope is shown in orange on XBB.1.5 RBD structure and Omi-42 binding footprint is depicted as a blue outline. (E) In silico modeling of L455W in a fully closed SARS-CoV-2 S structure (PDB 7K43; with S2M11 Fab bound) indicating expected clashes with a neighboring protomer. All energetically favored rotamers are sterically incompatible with the closed structure due to clashes with either the same or the neighboring protomer; one of the three most prevalent rotameric configurations was selected for visualization purposes. The two SARS-CoV-2 S protomers are shown in cyan and pink with the modeled L455W side chain highlighted as a red semi-transparent surface and steric clash indicated with a red asterisk. (F) Summary of MD simulation of XBB.1.5 RBD bound to ACE2 (dynamic) as compared to analysis of X-ray structure (static). Boxes indicate the sum of fraction occupancies of ACE2 contacts to each RBD residue; full glycans were modeled on the RBD:ACE2 structure, MD contacts [file NIHMS2027680-supplement-9.pdf]

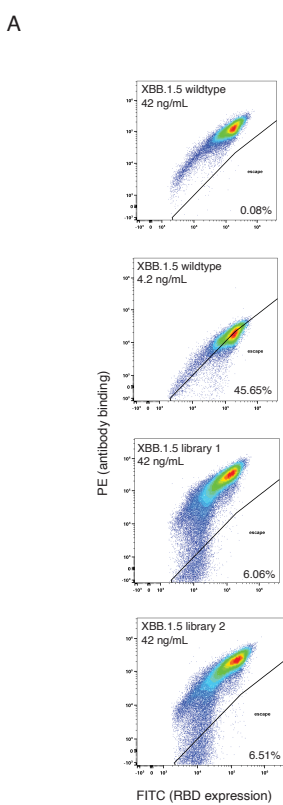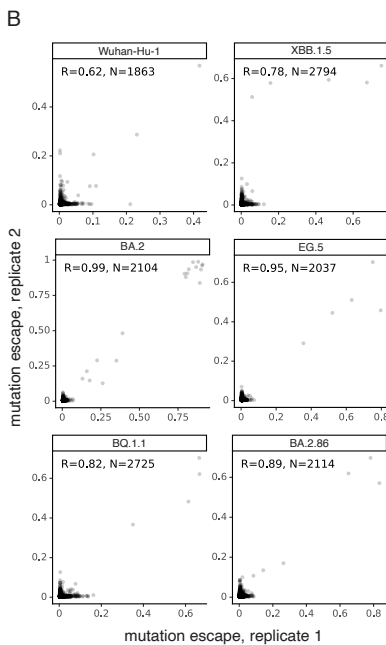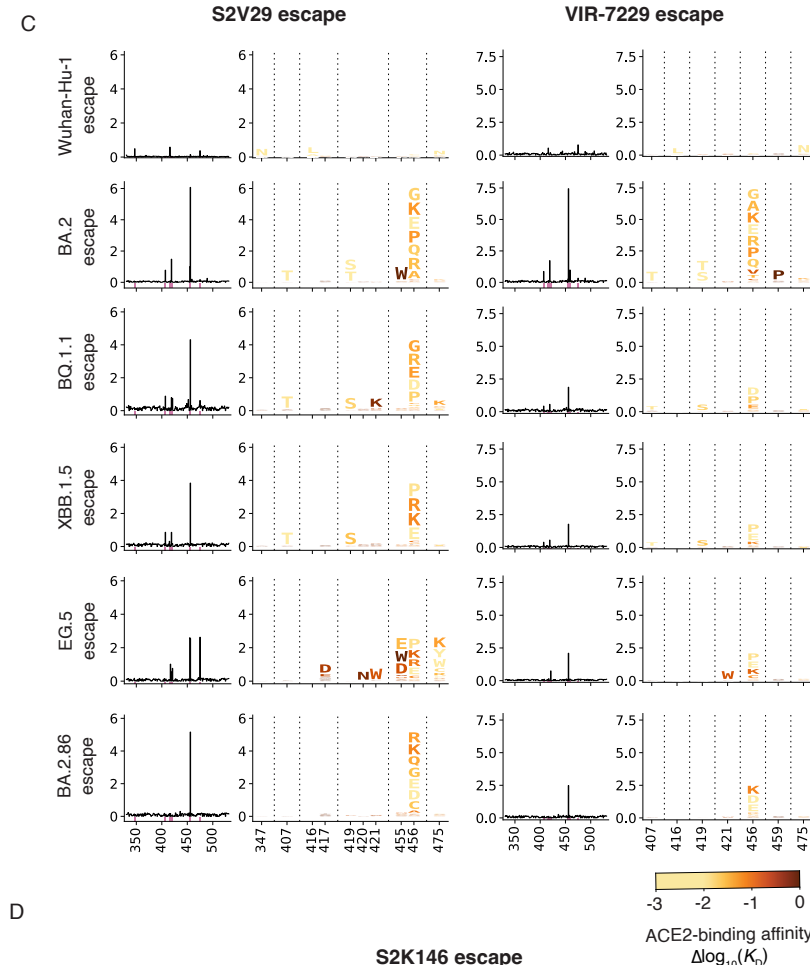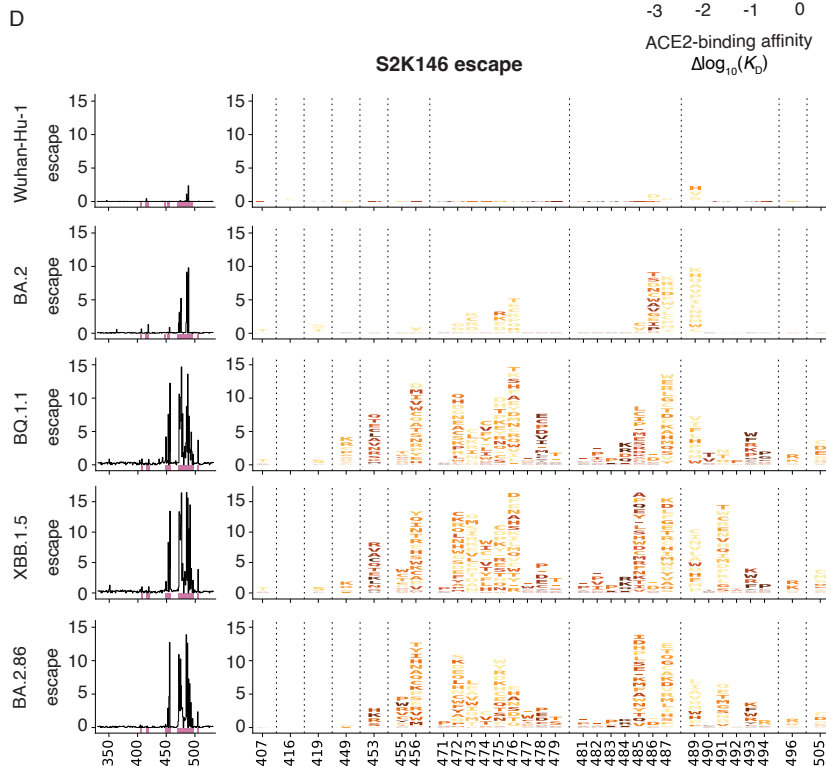

Supplement: 10 — Figure S5. Deep mutational scanning profiling of VIR-7229, S2V29, and S2K146 escape mutations. Related to Figure 5. (A) Representative FACS gates used to identify mutations that escape antibody binding. An antibody-escape gate was drawn that captures approximately 50% of the cells in the respective wildtype control labeled at 0.1x the library selection antibody concentration. The “escape fraction” represents the fraction of cells of a mutant genotype that fall into this antibody-escape FACS gate. (B) For each experiment with VIR-7229, the correlation in the per-mutation escape fraction between duplicate library selections. (C) Full deep mutational scanning escape profiles of parental mAb S2V29 (left) and VIR-7229 (right), compared to Figure 5A that illustrates VIR-7229 epitope profiles. For each experiment, lineplots (left) show the total escape at each site in the RBD, with sites of strong escape annotated with pink indicators. Logoplots (right) illustrate mutation-level escape fraction at sites of strong escape, with mutations colored according to mutational impact on ACE2-binding affinity. Note, mutations to T/S at sites 407 and 419 introduce N-linked glycosylation motifs due to the presence of N405 and N417 in the Omicron (but not Wuhan-Hu-1) variants. (D) Deep mutational scanning escape profiling of the comparator mAb S2K146 (details as in (C)) illustrating a broadening of the functional epitope over evolutionary time, likely due to erosion of S2K146 affinity across variant evolution. [file NIHMS2027680-supplement-10.pdf]

A

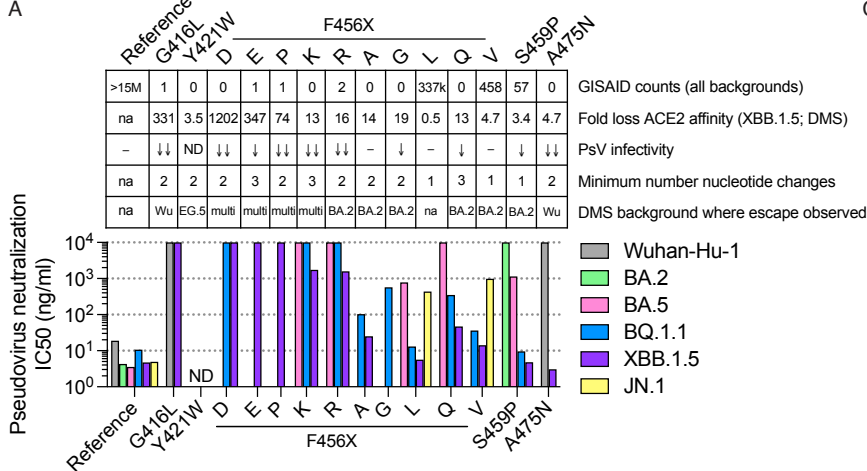

B

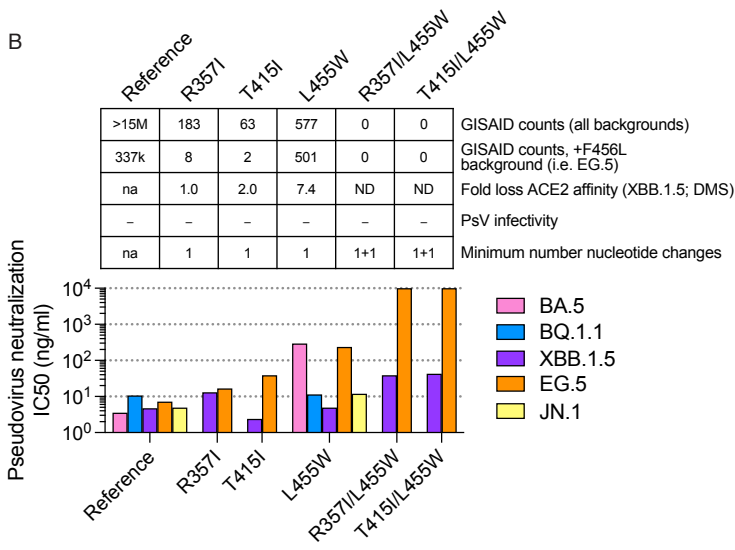

C

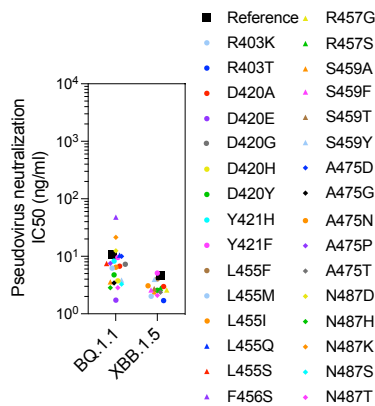

Supplement: 11 — Figure S6. Validation and fitness of VIR-7229 escape mutations. Related to Figures 5 and 6. (A) VIR-7229-mediated neutralization of SARS-CoV-2 pseudoviruses carrying mutations that were observed as DMS binding escapes in at least one strain background, plus F456L. Mutations were tested in different strain backgrounds, as indicated by bar color. Mutations are annotated by: (a) total counts in the GISAID database as of May 8, 2024, (b) fold-change reduction in ACE2 affinity in the XBB.1.5 background evaluated by DMS60 (available from https://tstarrlab.github.io/SARS-CoV-2-RBD_DMS_Omicron-XBB-BQ/RBD-heatmaps/), (c) impact on pseudovirus infectivity (assessed by evaluating the viral titer in comparison to the titer of respective unmutated backbone quantified in parallel; - indicates titers within 5-fold, ↓ indicates a titer more than 5-fold reduced in at least one backbone, ↓↓ indicates a titer more than 5-fold reduced in at least two backbones; see also Data S5), (d) minimum number of nucleotide changes required for the mutation to occur, and (e) the RBD background where the mutation was observed as a binding escape in the DMS experiment. Asterisk indicates that the reduced infectivity observed for A475N is only observed in the Wuhan-Hu-1 background. ND: Not determined; na: not applicable. See also Data S1. (B) VIR-7229-mediated neutralization of SARS-CoV-2 pseudoviruses carrying mutations that were observed during the EG.5 rVSV resistance selection experiment. Strain background is indicated by bar color. For L455W, JN.1 is also the BA.2.86 background (JN.1 = BA.2.86+L455S). Mutations are annotated as in panel A, as well as by counts in the +F456L background in the GISAID database as of May 8, 2024. See also Data S1. (C) VIR-7229-mediated neutralization of SARS-CoV-2 pseudoviruses carrying a set of epitope mutations below 0.005% frequency in GISAID but accessible by a single nucleotide change from wild-type sequences. Mutations were tested in either the BQ.1.1 or XBB.1 [file NIHMS2027680-supplement-11.pdf]
