# Supplementary material for: A potent pan-sarbecovirus neutralizing antibody resilient to epitope diversification
Source: Cell. Author manuscript; Available in PMC 2024 Dec 14. (PMC11645210; doi:10.1016/j.cell.2024.09.026)
Supplement: 8 — Figure S3. Cryo-EM data processing and validation of VIR-7229-bound BA.2.86 S. Related to Figure 3. (A, B) Representative electron micrographs (A) and class averages (B) of BA.2.86 S in complex with VIR-7229 Fab. Scale bars, 100 nm (A). (C) Gold-standard Fourier shell correlation curves for the S trimer bound to two VIR-7229 Fabs (black line) and the locally refined reconstruction of an RBD and VIR-7229 variable domains (grey line). (D, E) Local resolution map for the S trimer bound to two VIR-7229 Fabs (D) and the locally refined reconstruction of an RBD and VIR-7229 variable domains (E). (F) Cryo-EM data processing flowchart. [file NIHMS2027680-supplement-8.pdf]

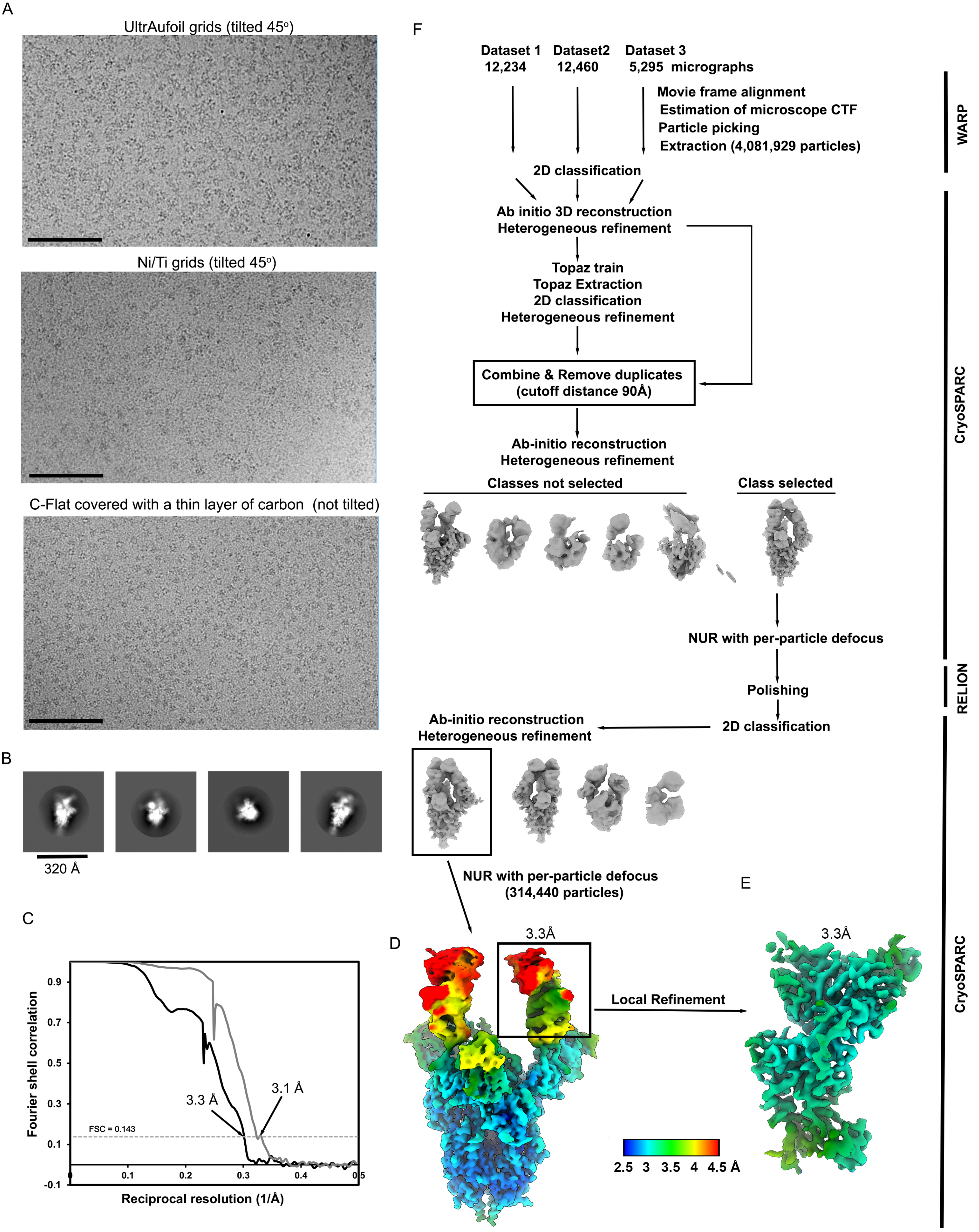

A
UltrAufoil grids (tilted 45°)
Ni/Ti grids (tilted 45°)
C-Flat covered with a thin layer of carbon (not tilted)
B
320 Å
C
Fourier shell correlation
Reciprocal resolution (1/Å)
FSC = 0.143
3.3 Å
3.1 Å
0.9
0.7
0.5
0.3
0.1
-0.1
0.1
0.2
0.3
0.4
0.5
D
3.3Å
Local Refinement
2.5 3 3.5 4 4.5 Å
E
3.3Å
F
Dataset 1 12,234
Dataset2 12,460
Dataset 3 5,295 micrographs
Movie frame alignment
Estimation of microscope CTF
Particle picking
Extraction (4,081,929 particles)
WARP
2D classification
Ab initio 3D reconstruction
Heterogeneous refinement
Topaz train
Topaz Extraction
2D classification
Heterogeneous refinement
Combine & Remove duplicates
(cutoff distance 90Å)
Ab-initio reconstruction
Heterogeneous refinement
Classes not selected
Class selected
CryoSPARC
NUR with per-particle defocus
Polishing
RELION
2D classification
Ab-initio reconstruction
Heterogeneous refinement
NUR with per-particle defocus
(314,440 particles)
CryoSPARC
