# Supplementary material for: A potent pan-sarbecovirus neutralizing antibody resilient to epitope diversification
Source: Cell. Author manuscript; Available in PMC 2024 Dec 14. (PMC11645210; doi:10.1016/j.cell.2024.09.026)
Supplement: 6 — Figure S1. Potency and breadth of comparator mAbs. Related to Figure 1. (A) Sequence variation among SARS-CoV-2 sequences in GISAID (blue, left) or sarbecoviruses (orange, right) mapped to the SARS-CoV-2 Wuhan-Hu-1 RBD structure (PDB: 6M0J). (B) Pseudovirus neutralization plotted versus mAb concentration for VIR-7229 and parent mAb S2V29, for Wuhan-Hu-1 and SARS-CoV-1, utilizing VeroE6 cells. (C-I) Neutralization of SARS-CoV-2 variant or sarbecovirus pseudoviruses mediated by S2V29 parent mAb and six comparator mAbs. Bar colors and horizontal lines as in Figure 1A; orange horizontal line for S309 indicates Vero-TMPRSS2 cells. S309, parent mAb of sotrovimab,15 and SA5542 were isolated from SARS-CoV-1 survivors, the latter after receipt of a vaccine based on SARS-CoV-2 Wuhan; S2K14633 and S2X25921 were isolated from SARS-CoV-2 patients after a pre-Omicron infection; Omi-42,27 like S2V29, was isolated from vaccinees following a breakthrough Omicron infection; VYD222 was affinity-matured from the ADI-55688 mAb.16,17,19 Asterisk indicates maximum neutralization plateaus at ~90% due to entry mediated by VSV-G (i.e. pseudovirus preparations with reduced titer). Orange data points are previously published: S2X25921 and sotrovimab.13 SARS-CoV-2 strains which completely escape Omi-42 all share the F456L mutation. See also Data S1. (J) VYD222 Fab fragment binding affinity to recombinant RBDs measured by SPR. Bar color denotes sarbecovirus clade, as in Figure 1C. SARS-CoV-1 RBD is the Urbani strain. See also Data S1. [file NIHMS2027680-supplement-6.pdf]

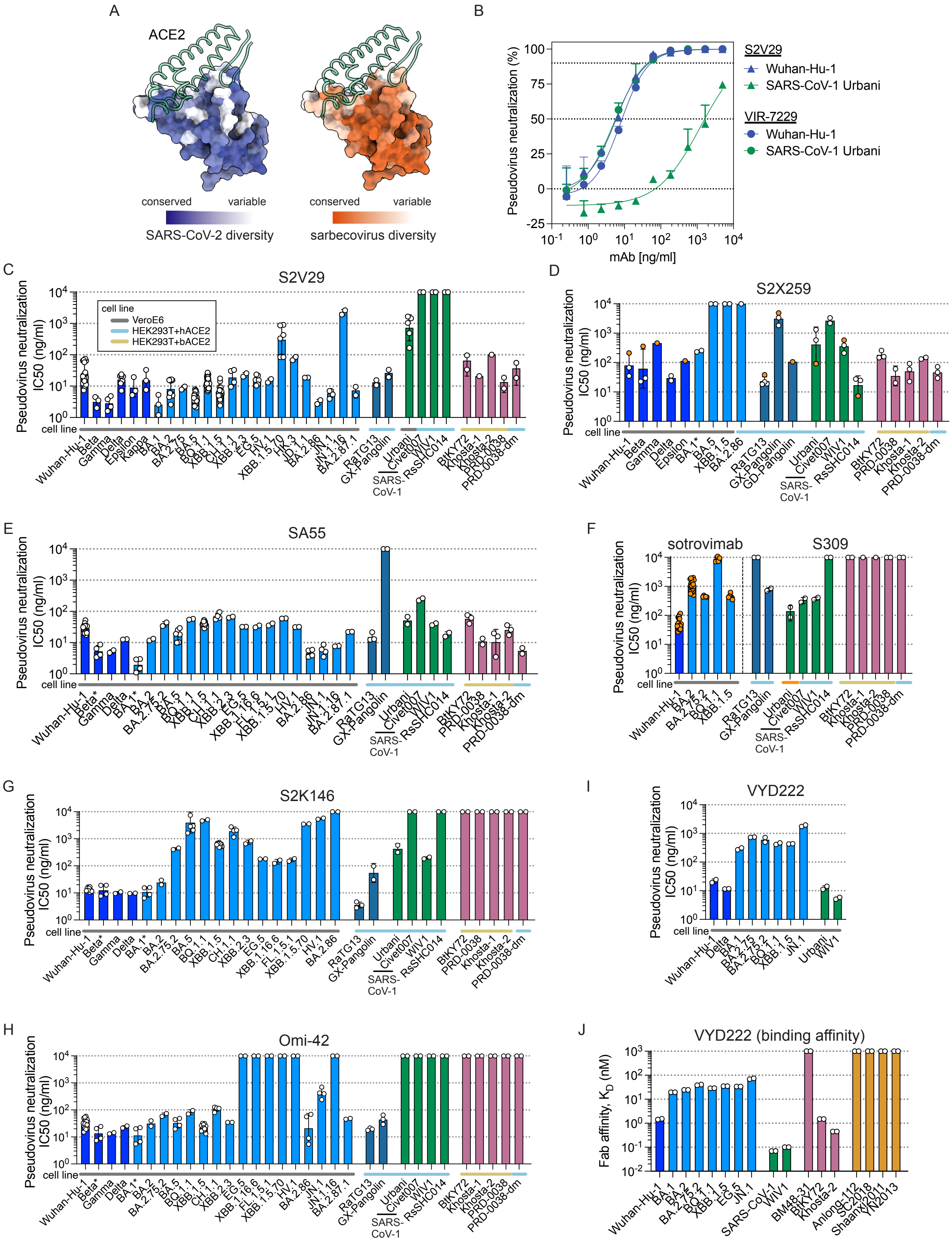

A
ACE2
conserved
variable
SARS-CoV-2 diversity
conserved
variable
sarbecovirus diversity
B
Pseudovirus neutralization (%)
mAb [ng/ml]
S2V29
Wuhan-Hu-1
SARS-CoV-1 Urbani
VIR-7229
Wuhan-Hu-1
SARS-CoV-1 Urbani
C
S2V29
Pseudovirus neutralization IC50 (ng/ml)
cell line
VeroE6
HEK293T+hACE2
HEK293T+bACE2
Wuhan-Hu-1
Beta
Gamma
Delta
Epsilon
Kappa
BA.1
BA.2
BA.2.75
BA.5
BQ.1.1
XBB.1.5
CH.1.1
XBB.2.3
EG.5
HV.1
XBB.1.5.70
HK.3
JD.1.1
BA.2.86
JN.1.16
BA.2.87.1
RaTG13
GX-Pangolin
Urbani
Civet007
WIV1
RsSHC014
SARS-CoV-1
BtKY72
Khosta-1
Khosta-2
PRD-0038
PRD-0038-dm
D
S2X259
Pseudovirus neutralization IC50 (ng/ml)
cell line
Wuhan-Hu-1
Beta
Gamma
Delta
Epsilon
BA.1*
BA.5
XBB.1.5
BA.2.86
RaTG13
GX-Pangolin
GD-Pangolin
Urbani
Civet007
WIV1
RsSHC014
SARS-CoV-1
BtKY72
PRD-0038
Khosta-1
Khosta-2
PRD-0038-dm
E
SA55
Pseudovirus neutralization IC50 (ng/ml)
cell line
Wuhan-Hu-1
Beta*
Gamma
Delta
BA.1*
BA.2
BA.2.75.2
BA.5
BQ.1.1
XBB.1.5
CH.1.1
XBB.2.3
EG.5
XBB.1.16.6
FL.1.5.1
XBB.1.5.70
HV.1
BA.2.86
JN.1.16
BA.2.87.1
RaTG13
GX-Pangolin
Urbani
Civet007
WIV1
RsSHC014
SARS-CoV-1
BtKY72
PRD-0038
Khosta-1
Khosta-2
PRD-0038-dm
F
sotrovimab
S309
Pseudovirus neutralization IC50 (ng/ml)
cell line
Wuhan-Hu-1
BA.2
BA.2.75.2
BQ.1.1
XBB.1.5
RaTG13
GX-Pangolin
Urbani
Civet007
WIV1
RsSHC014
SARS-CoV-1
BtKY72
Khosta-1
Khosta-2
PRD-0038
PRD-0038-dm
G
S2K146
Pseudovirus neutralization IC50 (ng/ml)
cell line
Wuhan-Hu-1
Beta*
Gamma
Delta
BA.1*
BA.2
BA.2.75.2
BA.5
BQ.1.1
XBB.1.5
CH.1.1
XBB.2.3
EG.5
XBB.1.16.6
FL.1.5.1
XBB.1.5.70
HV.1
BA.2.86
RaTG13
GX-Pangolin
Urbani
Civet007
WIV1
RsSHC014
SARS-CoV-1
BtKY72
PRD-0038
Khosta-1
Khosta-2
PRD-0038-dm
I
VYD222
Pseudovirus neutralization IC50 (ng/ml)
cell line
Wuhan-Hu-1
Delta
BA.1
BA.2.75
BA.2.75.2
BQ.1.1
XBB.1.5
JN.1
Urbani
WIV1
H
Omi-42
Pseudovirus neutralization IC50 (ng/ml)
cell line
Wuhan-Hu-1
Beta*
Gamma
Delta
BA.1*
BA.2
BA.2.75.2
BA.5
BQ.1.1
XBB.1.5
CH.1.1
XBB.2.3
EG.5
XBB.1.16.6
FL.1.5.1
XBB.1.5.70
HV.1
BA.2.86
JN.1
JN.1.16
BA.2.87.1
RaTG13
GX-Pangolin
Urbani
Civet007
WIV1
RsSHC014
SARS-CoV-1
BtKY72
Khosta-1
Khosta-2
PRD-0038
PRD-0038-dm
J
VYD222 (binding affinity)
Fab affinity, K_D (nM)
Wuhan-Hu-1
BA.1
BA.2
BA.2.75.2
BQ.1.1
XBB.1.5
EG.5
JN.1
SARS-CoV-1
WIV1
BM48-31
BtKY72
Khosta-2
Anlong-112
SC2018
Shaanxi2011
YN2013
