# Supplementary material for: A potent pan-sarbecovirus neutralizing antibody resilient to epitope diversification
Source: Cell. Author manuscript; Available in PMC 2024 Dec 14. (PMC11645210; doi:10.1016/j.cell.2024.09.026)
Supplement: 12 — Document S1. Figures S1–S6 and Tables S1–S3 [file NIHMS2027680-supplement-12.pdf]

Table S1: CryoEM data collection and refinement statistics, related to Figures 3 and S3.

	BA.2.86 S:VIR-7229<br>PDB 9AU2<br>EMD-43842	BA.2.86 S:VIR-7229<br>Local refinement RBD:VH/VL<br>PDB 9ASD<br>EMD-43813
**Data collection and processing**		
Magnification	105,000	105,000
Voltage (kV)	300	300
Electron exposure (e–/Å^2)	54	54
Defocus range (μm)	0.8-2.0	0.8-2.0
Pixel size (Å)	0.835	0.835
Symmetry imposed	C1	C1
Initial particle images (no.)	4,081,929	4,081,929
Final particle images (no.)	314,440	314,440
Map resolution (Å)	3.1	3.3
FSC threshold	0.143	0.143
**Validation**		
MolProbity score	1.39	1.16
Clashscore	3.71	1.82
Poor rotamers (%)	1.16	0.45
Ramachandran plot		
Favored (%)	96.93	96.58
Allowed (%)	2.98	3.42
Disallowed (%)	0.09	0

Table S2: Crystallographic data collection and refinement statistics, related to Figures 3 and S4.

	EG.5 RBD/<br>VIR-7229/S2H97<br>PDB 9ATM	XBB.1.5 RBD/<br>VIR-7229/S309<br>PDB 9AU1	BQ.1.1 RBD/<br>S2V29/S2H97<br>PDB 8S6M
Wavelength	1.195 Å	1.195 Å	1.00 Å
Resolution range	46.78 - 1.9 (1.92 - 1.9)	44.98 - 2.41 (2.45 - 2.41)	47.35 - 1.67 (1.69 - 1.67)
Space group	P 21 21 21	P 21 21 21	P 21 21 21
Unit cell	48.728 149.698 167.078 90 90 90	73.726 112.525 170.314 90 90 90	148.175 48.099 166.526 90 90 90
Total reflections	1277227 (33897)	734265 (36747)	941473 (16037)
Unique reflections	97295 (3168)	55433 (2722)	138930 (4582)
Multiplicity	13.1 (10.7)	13.3 (13.5)	6.8 (3.5)
Completeness (%)	99.91 (99.69)	99.88 (99.82)	99.96 (100.00)
Mean I/sigma I	12.8 (0.6)	13.2 (0.5)	15.5 (0.7)
Wilson B-factor	36.44	60.65	29.32
Reflections used in refinement	97295 (3017)	55433 (2599)	138930 (4349)
Reflections used for R-free	4870 (151)	2757 (123)	6987 (233)
R-work	0.1939 (0.4012)	0.2268 (0.4255)	0.1839 (0.3597)
R-free	0.2273 (0.4276)	0.2574 (0.4369)	0.2027 (0.3562)
CC(work)			
CC(free)			
Number of non-hydrogen atoms	8689	8190	8673
macromolecules	7977	8078	7844
ligands	36	42	59
solvent	676	70	770
Protein residues	1070	1070	1067
RMS(bonds)	0.007	0.008	0.006
RMS(angles)	0.87	0.96	0.87
Ramachandran favored (%)	98.19	95.72	98.08
Ramachandran allowed (%)	1.71	3.9	1.83
Ramachandran outliers (%)	0.10	0.38	0.1
Rotamer outliers (%)	0.47	2.36	0.73
Clashscore	2.18	3.89	1.9
Average B-factor	53.96	76.42	45.76
macromolecules	54.16	76.40	45.78
ligands	70.71	87.15	68.20
solvent	50.70	71.79	43.77

Table S3: Bioinformatic analysis of intra-individual SARS-CoV-2 genomic variability, related to Figures 6 and S6.

Nucleotide trimer change	Amino acid change	Genome-wide trimer mean mutation rate [%]	Standard error	Prevalence [%] of variant trimer at residue 455 in all backgrounds (GISAID)[1]	Prevalence [%] of variant trimer at residue 455 in F456L background (GISAID)[1]
**TG**T > TTT	L455F	0.0394	0.0086	0.40	18.51
**TG**C > TTC	L455F	0.0271	0.0053	0.25	12.65
**TTG** > TCG	L455S	0.0153	0.0014	0.31	0.04
**TG**T > TCT	L455F	0.0119	0.0014	0.02	1.19
**TG**C > TCC	L455F	0.0076	0.0015	4.1e-05	2.1e-03
A**TT** > AGT	L455V	0.0034	6.0e-04	3.4e-04	6.9e-04
**TTG** > TGG	L455W	0.0023	4.0e-04	3.7e-03	0.16
A**TT** > AAT	L455M	0.0021	4.0e-04	1.4e-04	0

The table depicts the in silico estimated mutation rate for all the nucleotide changes (in the context of their surrounding nucleotides) that can lead to an amino acid change at residue 455. Mean mutation rate and standard error are estimated from whole viral genomes of N=1763 samples (see details in Methods). Note, if the nucleotide change does not occur in the second nucleotide of the 455 amino acid codon, the trimer is not representative of the codon (e.g. for L455F, the variant occurs on the 3rd nucleotide of the 455 codon, the trimer therefore represents the last 2 nucleotides of the 455 codon and the first nucleotide of the 456 codon; for L455V and L455M, the variant occurs on the 1st nucleotide of the 455 codon, the trimers therefore represent the last nucleotide of the 454 codon and the first two nucleotides of the 455 codon). Nucleotides that are part of the wild-type Leucine codon are in bold. See also **Data S5**.

[1]GISAID prevalence is for sequences deposited up to January 17, 2024. As these results are based on the consensus sequences (as opposed to intra-individual variability), the result reflects viral fitness, immune pressure, transmission rate, as well as collection and submission biases (e.g. geographical).
